# A Systems-Based Key Innovation-Driven Approach Infers Co-option of Jaw Developmental Programs During Cancer Progression

**DOI:** 10.3389/fcell.2021.682619

**Published:** 2021-06-02

**Authors:** Stephan Marquardt, Athanasia Pavlopoulou, Işıl Takan, Prabir Dhar, Brigitte M. Pützer, Stella Logotheti

**Affiliations:** ^1^Institute of Experimental Gene Therapy and Cancer Research, Rostock University Medical Center, Rostock, Germany; ^2^İzmir Biomedicine and Genome Center, İzmir, Turkey; ^3^İzmir International Biomedicine and Genome Institute, Dokuz Eylül University, İzmir, Turkey; ^4^Department Life, Light & Matter, University of Rostock, Rostock, Germany

**Keywords:** cancer evolution, metastasis, jaw development, epithelial-mesenchymal transition, gnathostomes, cyclostomes, tumor evolutionary trajectories, prediction model

## Abstract

Cancer acquires metastatic potential and evolves via co-opting gene regulatory networks (GRN) of embryonic development and tissue homeostasis. Such GRNs are encoded in the genome and frequently conserved among species. Considering that all metazoa have evolved from a common ancestor via major macroevolutionary events which shaped those GRNs and increased morphogenetic complexity, we sought to examine whether there are any key innovations that may be consistently and deterministically linked with metastatic potential across the metazoa clades. To address tumor evolution relative to organismal evolution, we revisited and retrospectively juxtaposed seminal laboratory and field cancer studies across taxa that lie on the evolutionary lineage from cnidaria to humans. We subsequently applied bioinformatics to integrate species-specific cancer phenotypes, multiomics data from up to 42 human cancer types, developmental phenotypes of knockout mice, and molecular phylogenetics. We found that the phenotypic manifestations of metastasis appear to coincide with agnatha-to-gnathostome transition. Genes indispensable for jaw development, a key innovation of gnathostomes, undergo mutations or methylation alterations, are aberrantly transcribed during tumor progression and are causatively associated with invasion and metastasis. There is a preference for deregulation of gnathostome-specific versus pre-gnathostome genes occupying hubs of the jaw development network. According to these data, we propose our systems-based model as an *in silico* tool the prediction of likely tumor evolutionary trajectories and therapeutic targets for metastasis prevention, on the rationale that the same genes which are essential for key innovations that catalyzed vertebrate evolution, such as jaws, are also important for tumor evolution.

## Introduction

Despite advances in cancer management, manifestation of lesions with metastatic potential signals the terminal stage of disease. The term “metastatic potential” may include any combination of cancer phenotypes that enable metastatic dissemination including motility, immune evasion, and ability to survive in circulation and proliferate at distant sites (Birkbak and McGranahan, 2020). Cancer progression is governed by mechanisms distinct from those of initiation (Logotheti et al., 2020). While at early stages cancer cells accumulate driver mutations, at advanced stages, they do not acquire additional, metastasis-specific mutations (Rodrigues et al., 2018), but rather hijack programs of tissue-homeostasis and normal embryonic development and reactivate them in an unusual place, at the wrong time (Logotheti et al., 2020). Metastasis is promoted by aberrant gene regulation, and metastatic transcriptional programs arise from *de novo* combinatorial activation of multiple distinct and developmentally distant transcriptional modules (Rodrigues et al., 2018). We and others have shown that cancer progression is facilitated by ectopic activation of genes that have tissue-restricted profiles (Rousseaux et al., 2013; Richter et al., 2019), or are involved in placenta (Costanzo et al., 2018) and embryonic development (Billaud and Santoro, 2011) including, but not limited to, neuronal development and function (Logotheti et al., 2020). For instance, we have recently provided compelling evidence that genes involved in neuronal development and neurological function are reactivated in tumors and predict poor patient outcomes across various cancers. Tumors co-opt genes essential for the development of distinct anatomical parts of the nervous system, with a frequent preference for cerebral cortex and the neural crest-derived enteric nerves function (Logotheti et al., 2020). In this respect, co-option, the evolutionary process through which a biological function within a specific context may be alternatively used in another context to support a novel function, emerges as a recurrent and prevailing pattern during tumor progression (Billaud and Santoro, 2011).

The gene regulatory networks (GRN) of embryonic development and tissue homeostasis are encoded in the genomes of animal species and define their attributes and morphogenetic complexity (Levine and Davidson, 2005). Considering that tumors can progress to metastatic stages by co-opting such gene programs, it is reasonable to conjure that the metastatic potential largely depends on the gene reservoir of the species on which tumors grow. For example, lesions growing on animals as simple as cnidarians will plausibly usurp the GRNs controlling their corresponding attributes, while mammal tumors have access to GRNs underlying more sophisticated body plans. From a phylogenetic point of view, all metazoa have evolved from a common ancestor via major macroevolutionary events, which advanced the animal body plans, and GRNs which are associated with these events are conserved across species (Levine and Davidson, 2005). Given that these GRNs can be inevitably at the disposal of cancerous tumors, we wondered whether the same key innovations through which the species evolved may have been, in parallel, exploited by the primary tumor, in order to evolve toward metastatic stages. The term “key innovation,” as used herein, refers to any novel phenotypic trait that facilitates adaptive radiation and evolutionary success of a taxonomic group (Hunter, 1998), as well as the respective genes and/or GRNs that support establishment of this trait. The epithelial-mesenchymal transition (EMT), that is the hierarchical GRN which controls neural crest, a vertebrate-specific multipotent embryonic cell population which generates several body anatomical structures (Martik and Bronner, 2017) is a representative example of co-option of key innovations toward enhancing metastatic potential. Indeed, the same EMT factors mediating differentiation and migration of neural crest are also ectopically reactivated during tumor progression (Kerosuo and Bronner-Fraser, 2012; Fürst et al., 2019).

All metazoa can develop tumors (Domazet-Lošo et al., 2014), but major differences in their prevalence and metastatic potential are observed across phyla. The “big-bang” of tumor formation is traced to Cnidaria and correlates with the emergence of multicellularity. All cancer-associated genes are conserved in Cnidaria, and *Hydra* tumor cells have an invasive capacity (Domazet-Lošo et al., 2014). However, the aforementioned phylogenetic origin of tumor formation does not coincide with the phenotypic manifestations of aggressiveness, since Cnidaria neither form true metastases nor die of cancer (Domazet-Lošo et al., 2014). Thus, it remains enigmatic how species with lethal cancers have non-metastatic common ancestors, as well as if there are any key innovations that may be linked with increased prevalence of metastasis across the metazoa clades. We hypothesized that if certain key innovations increase organismal fitness of a given species population, they will likely undergo positive selection, despite the risk of being co-opted by the tumors later on in the life of the individuals of the respective population. To explore whether organisms that inherited key innovations from a common ancestor consistently manifest metastatic potential, in contrast to the ones which lack them, we applied phylogeny, that is, the evolutionary history of species in relation to oncogeny (Dawe, 1969). Herein, we revisited and retrospectively juxtaposed cancer reports across taxa on the same evolutionary lineage with mammals, from cnidarians to humans, and then integrated cancer phenotypes of these species with high-throughput data from up to 42 human cancer types, data on developmental phenotypes of knockout mice, and phylogenetic comparative methods. This multidisciplinary meta-analysis allowed us to infer that phenotypic manifestation of metastasis coincides with agnatha-to-gnathostome transition. Genes essential for jaw development, which is a key innovation of gnathostomes, are deregulated in tumor cells and are causatively associated with tumor progression.

## Materials and Methods

### Identification of Jaw-Indispensable Genes

To identify those genes that are indispensable for the development of cartilaginous jaws (JIGs), we screened the Mouse Genome Informatics (MGI) database (Bult et al., 2019) for genes the knockout of which leads to mouse phenotypes with jaw-related defects, as recently described (Logotheti et al., 2020), using the terms “cartilage,” “jaws,” “mandible/mandibular,” “maxilla,” and “micrognathia.” Their human orthologs were identified and the official HUGO gene nomenclature committee (HGNC) (Gray et al., 2015) gene symbols were used (Supplementary Table 2).

### Orthologs Search

The HGNC symbols of the 305 jaw-indispensable genes (JIG; Supplementary Table 2) were used initially to retrieve the corresponding, well-annotated, gnathostome (*Homo sapiens*, *Mus musculus*, *Gallus gallus*, *Xenopus laevis*, *Danio rerio*, and *Callorhinchus milli*) protein sequences from the publicly available non-redundant sequence database NCBI RefSeq (O’Leary et al., 2016). The canonical or longest known transcripts per protein were selected. For obtaining orthologous sequences from the agnatha genera under study (Petromyzon, Branchiostoma, Ciona, Strongylocentrotus, and Hydra), the retrieved gnathostome sequences were used as probes to iteratively search the agnathan genomes available in NCBI RefSeq and GenBank (Sayers et al., 2019), by applying reciprocal BLASTp and BLASTn (Altschul et al., 1990) with default parameters; an in-house Python script was employed (available on request). The protein domain organization of the novel sequences was explored through SMART v.8.0 (Letunic and Bork, 2018).

To identify the “true orthologs” of each JIG/protein, phylogenetic trees (a total of 305) of the homologous, gnathostome and agnathan, protein sequences were constructed. To this end, the amino acid sequences of the homologous proteins were aligned using Clustal Omega, version 1.2.4 (Sievers and Higgins, 2014a,b) and the resulting multiple sequence alignment was provided as input to the software package MEGA version 10.1 (Kumar et al., 2018) in order to perform phylogenetic analyses, by employing a neighbor-joining (NJ) and a maximum likelihood (ML) method. The expected number of amino acid substitutions per position was estimated with the JTT model (Jones et al., 1992). The robustness of the inferred phylogenetic trees was evaluated by bootstrapping (100 pseudo-replicates). Only those agnathan sequences that clustered with the known gnathostome sequences under study were considered as “true orthologs.” A characteristic example is shown in Supplementary Figure 1.

### Functional Interaction Networks and Gene Set Enrichment Analysis

The associations among the jaw-indispensable human genes/protein products were investigated in the STRING v11.0 database (Szklarczyk et al., 2019), by selecting a high confidence interaction score (≥0.9). Moreover, Cytoscape v3.8.0 (Shannon et al., 2003), was employed for network processing, visualization and statistical analysis. For the Gene Set Enrichment Analysis, the GSEA-P 2.0 software (Broad Institute, Cambridge, MA, United States) (Subramanian et al., 2005) was used. Enriched hallmark and Gene Ontology terms were plotted against the negative log10 of their individual FDR value (<0.05).

### Meta-Analyses of Mutation and Methylation Data From Human Tumors

We juxtaposed the datasets of PanCancer and GENCODEv32 using Ensembl gene IDs and filtered for protein coding genes with a transcript support level TSL < 3, to generate a comprehensive dataset of 19,617 transcripts. Recently identified cancer driver genes and frequently mutated genes were also used (Bamford et al., 2004; Kandoth et al., 2013; Bailey et al., 2018). As control gene lists, we generated 100 lists of 305 random genes by sampling the 19,313 transcripts (without JIGs) without replacement (available on request). The Cancer Gene Census (CGC) list of 723 genes (including two non-coding) was downloaded from the COSMIC (Catalogue of Somatic Mutations in Cancer) website (Bamford et al., 2004) and juxtaposed with JIGs or random lists. For evaluating stochastic events, we calculated the percentage of events by chance (*x*% = 100 × *k*/19,313 coding genes without JIGs, *k* = 721 CGC, 299 cancer drivers, 127 frequently mutated) and performed Chi-squared test (stochastic events) or *z*-test (random gene lists). The number of patients affected by gene mutations and the number of mutations per gene were retrieved from the GDC data portal (Jensen et al., 2017) after uploading the respective gene list. DiseaseMeth database (Xiong et al., 2017) was used to detect the differentially methylated JIGs in several cancer types. All results were downloaded and data for the different cancer types were pooled.

### Transcriptome Analysis in CCLE, TCGA, and GEO Databases

Cancer Cell Line Encyclopedia (CCLE) and The Cancer Genome Atlas (TCGA) transcriptomic data were analyzed as recently described (Logotheti et al., 2020). For identifying differentially regulated transcripts in metastatic versus primary lesions or normal tissue, transcriptome data from the Gene Expression Omnibus (GEO) database (Clough and Barrett, 2016) (study numbers: GSE21510, GSE2509, GSE25976, GSE43837, GSE468, GSE6919, GSE7929, GSE7930, GSE8401) were analyzed by GEO2R. Cox regression analysis was performed by R software using the *coxph* function in the *survival* package.

### Statistical Analysis

Unless otherwise stated, statistics were performed by Student’s *t*-test; *p* values less than 0.05 were considered as significant (*p < 0.05, **p < 0.01, ***p < 0.001). All statistical tests were two-sided.

## Results

### Key Innovations in Relation With Tumor Characteristics Across Phylogenetic Taxa: Available Resources and Considerations

As a framework for comparing tumors among metazoa, we used the one proposed by Dawe, one of the pioneers to address oncogeny in relation to phylogeny. The framework is the phylogenetic tree *per se* and the comparisons are made by ascending the bifurcating tree, in a direction from the last common ancestor to the more recent taxon, by recording changes which occurred proximal to the divergent branches at each node (Dawe, 1969). In line with this, we selected that taxon in each bifurcation of the phylogenetic tree in which an indispensable hallmark macroevolutionary trait occurred first, and was conserved throughout the descendant lineage, up to mammals and humans (Hickman et al., 2001). Porifera was the baseline taxonomic group of our study panel, since sponges do not develop apparent tumors (Domazet-Lošo et al., 2014). In the case of Protochordates, due to their nodal position between invertebrates and vertebrates (Delsuc et al., 2006), both children taxa (Cephalochordates and Urochordates) were included in the analysis.

**FIGURE 1:**
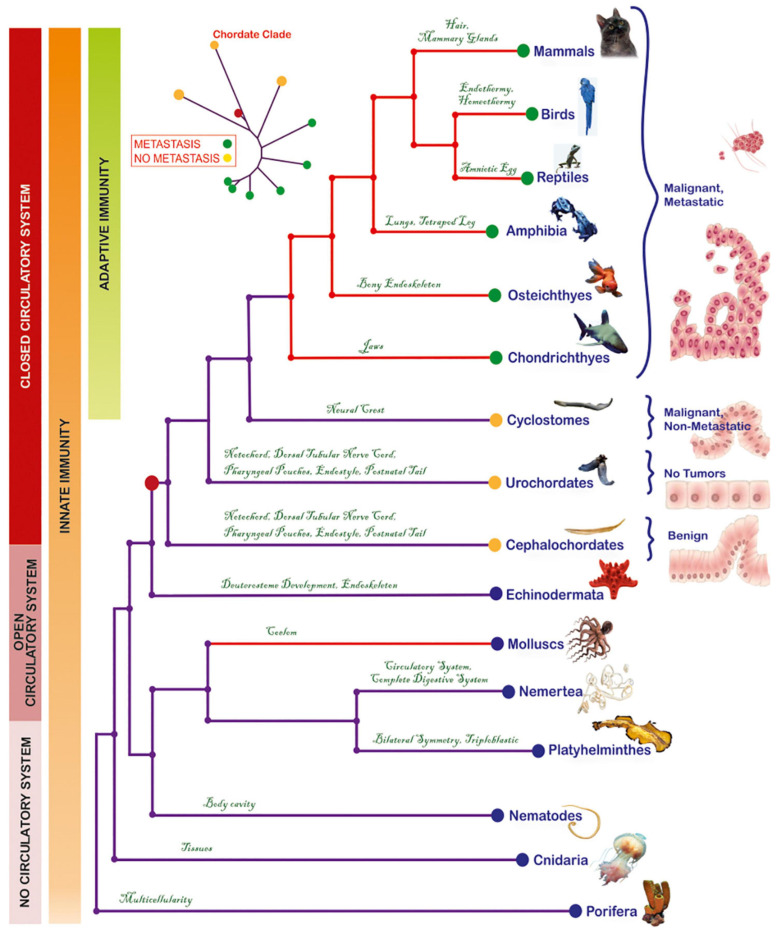
Phylogenetic tree depicting the evolutionary relationships of metazoa and the emergence of metastasis in the cyclostome-to-chondrichthyes transition. Each phylogenetic group has manifested, for the first time, a key innovation that was conserved throughout the lineage. Red-colored clades indicate groups with lethal cancer types. Based on information derived from histological analyses, tumors were classified into benign (corresponding to initiation), malignant/non-metastatic (corresponding to promotion) or malignant, metastatic (corresponding to progression). Invasive and metastatic tumors have been reported from Chondrichthyes to mammals. The mollusks present contagious invasive cancer, while the urochordates are cancer-resistant. The red dot on the tree represents the common chordate ancestor, which gave rise to both, metastatic (green dots) and non-metastatic species (yellow dots). Model of the progression from a normal cell to metastatic cancer was modified from a previous illustration (Iacobuzio-Donahue et al., 2012). The origins of a closed circulatory system and of acquired immunity are also presented.

We extensively searched publicly available databases, for peer-reviewed cancer reports on representatives of these taxa. A degree of inherent heterogeneity due to diverse experimental methods across these reports should be assumed. Notwithstanding, these records are, to date, the best available source of information on tumor characteristics of these taxa. They remain fundamental, especially given that in some species, experimental carcinogenesis protocols cannot be applied, due to animal ethics restrictions and/or because they do not represent conventional laboratory animal models. To eliminate potential bias by comparing pathology reports on isolated cases, we particularly emphasized on studies which (a) followed-up large populations of animals over ample periods of time, and (b) provided a clear number of subjects, histological characterizations, and wherever possible, experimental validations. Another comprehensive source of information is the “Registry of Tumors in Lower Animals” (RTLA), i.e., an official repository of validated tumor reports from a large number of invertebrates and cold blooded vertebrates (Harshbarger, 1969), and the “Overall five year progress report for the registry of tumors in lower animals from September 30, 2002 through September 30, 2007” (personal communication, Dr. P.J. Daschner). We also considered parameters that are particularly important for metastasis, mainly (a) the circulatory system, which offers cancer cells a means for energy supply and migration to secondary sites (Hanahan and Weinberg, 2011) and (b) the immune system, which reflects the innate ability of an organism to detect and eliminate malignant cells and represents a major evolutionary pressure in the tumor microenvironment (Angelova et al., 2018). All chordates have a closed circulatory system (Hickman et al., 2001). Adaptive immunity first occurred in cyclostomes (Cooper and Alder, 2006), whereas all their ancestors possess only innate immunity (Langlet and Bierne, 1982; Cooper and Alder, 2006; Maciel and Oviedo, 2018). Overall, tumors across the selected taxa, in association with aggressiveness, immunity and the circulatory system can be overviewed in Figure 1 and Supplementary Table 1.

### Tumor Landscape in Pre-vertebrates

According to available reports (Supplementary Table 1), Cnidaria are the most ancient organisms known to develop naturally occurring tumors. Although tumors in Hydra polyps reduce capacity for egg production and rate of population growth, they are non-lethal for the affected individuals (Domazet-Lošo et al., 2014). Platyhelminthes develop spontaneous, non-lethal tumors (Harshbarger, 1969; Tascedda and Ottaviani, 2014), while their exposure to carcinogen type 1A cadmium leads to benign tumors and impairment of their regenerative ability, especially in combination with inactivation of tumor-suppressor genes (Van Roten et al., 2018). No tumors have been reported in Nemertea. Nematodes develop germline cell-derived tumors (Kirienko et al., 2010). Cancer in Molluscs is manifested as a leukemia-like, disseminated neoplasia (DN), and as germinal cell-derived gonadal neoplasia (Barber, 2004; Carballal et al., 2015). Importantly, DN in *Mya arenaria* populating the coast of North America, is a horizontally transmissible form of cancer, whereby the cancer clone, which likely arose in a single individual, is spread to host clams, and bears a genotype distinct from the host genotypes (Metzger et al., 2015). Nevertheless, such deadly tumors are restricted to bivalvia, and, to date, have not been described in other pre-vertebrate taxa on the same evolutionary lineage. Indeed, Echinoderms are resistant to chemical-induced oncogenesis (Wellings, 1969), and either lack spontaneous tumor lesions (Wellings, 1969) or develop non-invasive/non-lethal, pigmented lesions (Fontaine, 1969). Similarly, the protochordates either appear to be cancer-free (Urochordates) (Dawe, 1969; Wellings, 1969) or form benign tumors (Cephalochordates) (Wellings, 1969). Overall, with the exception of Molluscs, tumors in pre-vertebrates are not associated with lethal outcomes (Figure 1).

### Emergence of Metastasis Coincides With Agnatha-Gnathostomes Split Within the Vertebrate Clade

**FIGURE 2:**
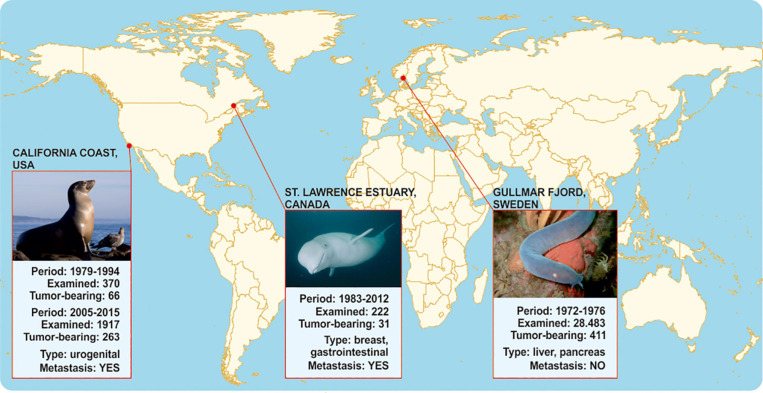
Comparative epidemiology data on metastases in agnathan versus gnathostome representatives that inhabit comparable carcinogen-contaminated aquatic environments. No metastases have been reported in hagfishes inside and outside Gullmar fjord. On the contrary, toothed belugas in Quebec as well as California sea lions that inhabit environments polluted with similar organochlorides are susceptible to metastatic tumors.

Cyclostomes, the only living jawless vertebrates (agnatha) (Gess et al., 2006), comprise a monophyletic group (Heimberg et al., 2010), including Petromyzontia (lampreys) and Myxinoidei (hagfishes). Until 70’s, only one case of cyclostome cancer had been reported in RTLA. Following this singleton report, Falkmer and colleagues addressed cyclostome tumor pathology in an extensive, thorough and well-controlled manner (Falkmer et al., 1976, 1978; Falkmer, 1998) and, up to this day, this seminal work remains the most comprehensive source of information for carcinogenesis on this enigmatic, though basal, vertebrate superclass. In particular, Falkmer screened, for tumor incidence, two large populations of lampreys caught in Ume/Ricklean rivers, and hagfishes caught inside and outside the Gullmar fjord. In a population of 6,000 lampreys, only one individual (0.017%) presented highly differentiated primary hepatocellular carcinoma. In contrast, tumors were detected in the hagfish population inside the fjord, and this percentage was significantly enhanced versus the tumor-bearing individuals in the open-sea control group (Figure 2). Of the 27,300 hagfishes captured within a 5-year period (1972–1976), up to 5.8% exhibited liver neoplasms (adenomas and carcinomas). This percentage was significantly higher compared to the tumor-bearing individuals (2.8%) in the control, open-sea population of 1,183 hagfishes caught outside the fjord. Although the affected hagfishes developed high- or low-differentiation tumors, they showed no macroscopic signs of metastasis (Falkmer et al., 1976, 1978; Falkmer, 1998). This increased cancer incidence was attributed to a combination of polychlorinated biphenyls (PCBs) and dichlorodiphenyltrichloromethylmethane (DDT), two anthropogenic organochloride contaminants which entered the fjord via washout. Due to parasitism of hagfishes on PCB/DDT-contaminated fishes, the organochlorides bioaccumulated in their liver or pancreatic islets, eventually triggering oncogenesis at these sites (Falkmer et al., 1978). The fact that despite their exposure to confirmed carcinogens (Lauby-Secretan et al., 2013; Loomis et al., 2015; Abu-Helil and van der Weyden, 2019), a percentage developed malignant tumors, but none of the 28,483 study individuals developed metastasis, leads to the suggestion that these animals may be metastasis-refractory or metastasis-incapable. Nevertheless, metastatic capability is evident in all descendant lineages. In particular, in Chondrichthyes, at least 50 cases of spontaneous cancer, including invasive (Wellings, 1969) and metastatic (Schlumberger and Lucke, 1948; Ostrander et al., 2004) tumors, have been recorded; in Osteichthyes, the tumor incidence increases and metastatic cases become more frequent (Schlumberger and Lucke, 1948; Wellings, 1969; Couch and Harshbarger, 1985; Groff, 2004); in amphibia (Stacy and Parker, 2004), reptiles (Abu-Helil and van der Weyden, 2019), birds (Abu-Helil and van der Weyden, 2019), and mammals (Albuquerque et al., 2018), there is an increased prevalence of invasive and metastatic cancers (Albuquerque et al., 2018). Collectively, a retrospective overview of tumor reports suggests the consistent occurrence of metastases in chondrichthyes and their descendants, providing hints that the establishment of metastatic potential coincides with the agnatha-to-gnathostome transition within the vertebrate clade (Figure 1).

The complicated life-cycle of cyclostomes, as well as ethical considerations regarding their research (Shimeld and Donoghue, 2012), challenge the application of experimental carcinogenesis protocols on adult individuals to simulate Falkmer’s field study on a laboratory scale (Shimeld and Donoghue, 2012). Hence, as a surrogate test for corroborating associations between agnatha-to-gnathostome transition and metastatic potential, we sought to juxtapose Falkmer’s nodal tumor pathology reports on adult cyclostomes to those of gnathostome representatives living in comparable PCB/DDT-contaminated environments (Figure 2). In particular, Beluga whales and California sea lions inhabit aquatic environments with persistent organic pollutants similar to those reported for the Gullmar fjord, and show unusually high cancer prevalence among marine mammals (Deming et al., 2018), which has been associated with exposure to carcinogens (Ylitalo et al., 2005; Randhawa et al., 2015; Lair et al., 2016). As shown in Figure 2, in an isolated population of about 900 toothed belugas (*Delphinapterus leucas*) living in the heavily industrialized St Lawrence Estuary of Quebec, Canada, cancer was reported as one of the most frequent causes of death (14%, 31/222 animals) in 222 carcasses found stranded or drifting, from 1983 to 2012. Tumors were often metastatic and fatal, and associated with the gastrointestinal tract (adenocarcinoma of the gastrointestinal mucosa, salivary gland and cholangiocellular carcinoma) and mammary glands. Exposure to carcinogens has been associated to increased cancer incidence (Lair et al., 2016), since living and dead beluga tissues were heavily contaminated by agricultural and industrial contaminants, including PCB/DDTs and their metabolites (Martineau et al., 2002). Similarly, between 1979 and 2015, necropsies of 2,287 sea lions beached-off central California coast in United States revealed high cancer incidence, where the predominant neoplasms were poorly differentiated urogenital carcinomas, with frequent local invasions and widespread metastases (Lipscomb et al., 2000; Deming et al., 2018). Metastasis was diagnosed in 18% (66/370) of necropsied animals from 1979 to 1994. From 2005 to 2015, 14% (263/1917) of cases had cancers, the vast majority of which were metastatic (Lipscomb et al., 2000; Deming et al., 2018), localized in the urogenital tissues and associated with organochloride bioaccumulation (Ylitalo et al., 2005; Randhawa et al., 2015). Hence, in similar carcinogenic environments, gnathostome species appear susceptible to aggressive cancers in contrast to the metastasis-refractory Gullmar fjord hagfishes. Similarly, organochloride pollutants have been correlated with risk of metastasis in human breast cancer patients (Koual et al., 2019). The aforementioned observations suggest that gnathostomes might be more prone to metastasis than agnatha upon exposure to carcinogenic pollutants. If this is indeed the case, then macroevolutionary gains of gnathostomes, such as the cartilaginous jaws, emerge as a key innovation possibly associated with metastasis.

### JIGs Undergo Frequent Mutations and DNA Methylation Alterations in Human Cancers

**FIGURE 3:**
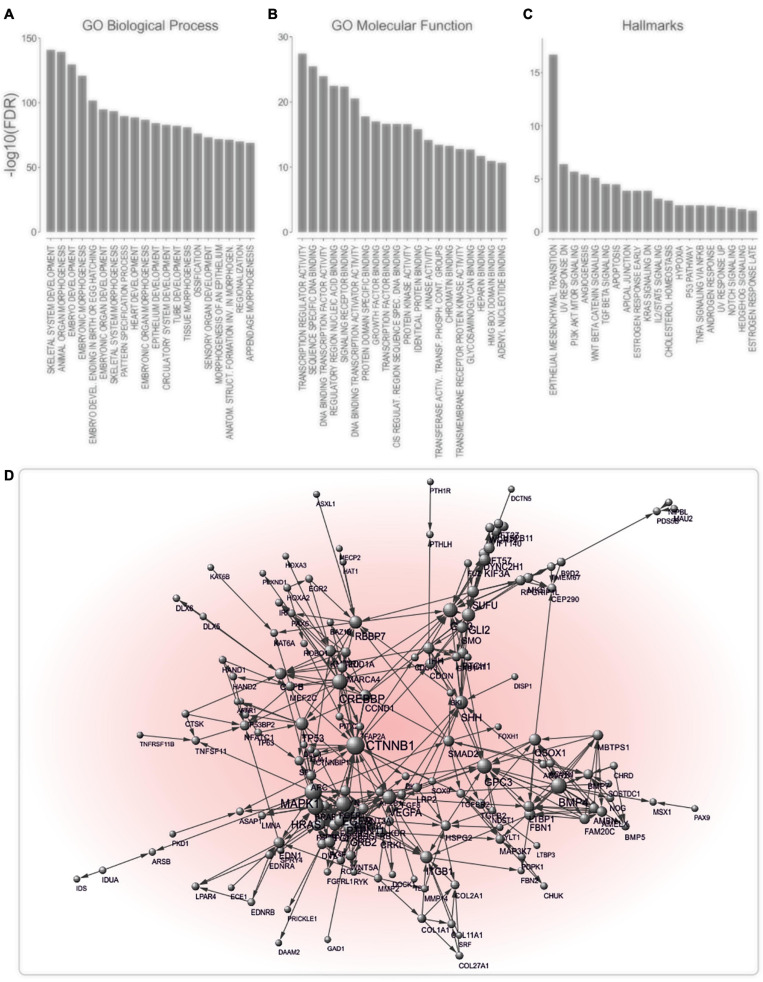
JIGs exhibit a high degree of interconnectedness. **(A–C)** Analysis of enriched GO biological processes: **(A)** GO molecular functions, **(B)** and hallmark processes, **(C)** indicates a role of JIGs in the development and EMT, mainly via affecting transcriptional regulation. **(D)** Interaction network of JIGs (interaction confidence score ≥ 0.9) shows that 173 of 305 genes/proteins are highly interconnected. The nodes represent genes/proteins and the connecting edges functional associations. Node sizes reflect node degree.

Agnatha-to-gnathostomes transition was promoted by the evolution of a cartilaginous skull, along with the establishment of jaws. These novelties facilitated the emergence of a complex brain and senses, that, together with the pharyngeal cartilage, allowed gnathostomes to shift to active predation, intermittent feeding and behavioral diversification (Kaucka and Adameyko, 2019). They highlight vertebrates’ evolutionary success and, thus, are conserved from chondrichthyes to human (Kaucka and Adameyko, 2019). Based on the observation that the phenotypic manifestations of metastasis coincide with the evolutionary time point of occurrence of gnathostome key innovations, we used computational methods to unravel links between jaw formation and metastatic potential. We postulated that if our hypothesis is valid, then genes supporting formation of cartilaginous jaws would tend to be deregulated during cancer progression. To identify genes essential for the development of cartilaginous jaws, we screened the Mouse Genome Informatics (MGI) database for knockout-mice phenotypes that encompass jaw-related defects. In this way, 305 JIG were identified, all of which exhibit highly conserved human orthologs (Supplementary Table 2). The term “JIG,” as used herein, refers to any gene which, if impaired, leads to abnormal jaw embryonic development. Notably, as indicated by the calculated ratios of jaws phenotypes to all affected phenotypes (Supplementary Table 2), JIGs are not functionally restricted only to jaw development, however mutation in even one of them leads to jaw malformations. GSEA revealed JIGS’ involvement in skeletal system and cartilage development, appendage morphogenesis, and pattern specification (Figure 3A); molecular functions such as DNA binding, transactivation activity and signaling receptor binding (Figure 3B); and hallmark processes like Wnt-beta, TGF-beta and NOTCH signaling, and EMT (Figure 3C). STRING analysis indicated that 173 of these factors form a highly interconnected network (Figure 3D). Altogether, these data suggest that JIGs interact either physically or functionally within the context of jaw formation, to create a network, to which we, hereafter, refer to as jaw-developmental network (JDN).

**FIGURE 4:**
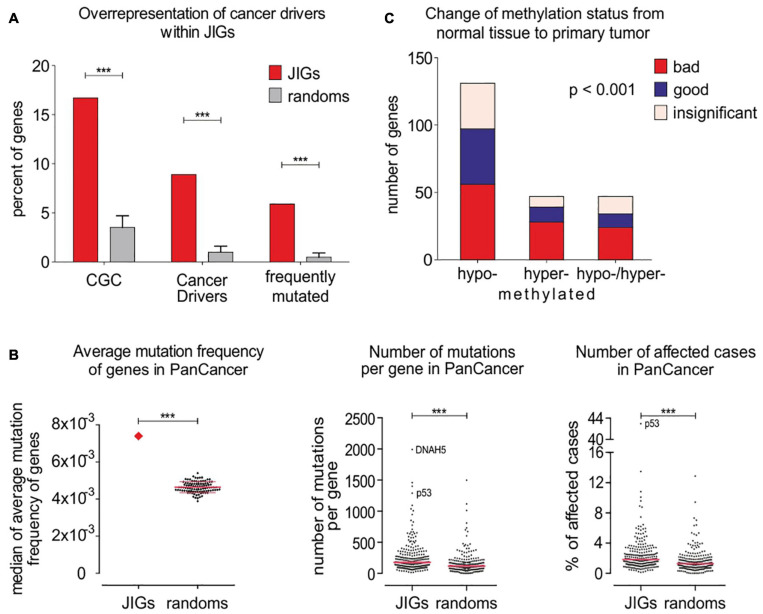
JIGs undergo frequent gene mutations and epigenetic alterations across cancer types. **(A)** JIG enrichments in known and frequently mutated cancer drivers as indicated versus controls were 16.7% vs. 3.5 ± 1.2% (*p* < 0.001), **(left)**; 8.9% vs. 1.0 ± 0.6% (*p* < 0.001), **(middle)**; and 5.9% vs. 0.5 ± 0.4% (*p* < 0.001), **(right)**.**(B)** The average mutation frequency of JIGs is higher in PanCan cohort versus genes of the random lists **(left)**. This entails a higher number of mutations per individual JIG **(center)** and a higher number of patients exhibiting mutations in JIGs **(right)**. Pink lines: medians. **(C)** Almost 74% (225/305) of JIGs undergo aberrant DNA methylation in 42 cancer types versus normal control tissues, whereby 131 are significantly hypomethylated, 47 are hypermethylated, and 47 are either hypomethylated or hypermethylated in a cancer type-dependent manner. Cox regression analysis on PanCan TCGA cohort data showed that more hypo- than hyper- methylated JIGs are correlated with either poor (“bad,” red color) or favorable (“good,” blue color) patient outcomes. Bars represent SEM.

Next, we examined whether JIGs undergo frequent genetic and/or epigenetic alterations in tumors. First, we juxtaposed three distinct lists of identified cancer gene drivers and mutations (Bamford et al., 2004; Kandoth et al., 2013; Bailey et al., 2018) with the list of JIGs to determine the number of JIGs that are mutated across human cancer types. To ensure that the association is non-random, we compared to 100 control lists each encompassing an equal number of 305 random, unrelated genes (available on request). A significant enrichment of CGC factors was observed among the JIGs 16.7%, or 51 genes) relative to random gene lists of the same size (3.5 ± 1.2%) and relative to the expected value (3.7%, or 721 of 19,313 coding genes from GENCODEv32, *p* < 0.001) (Figure 4A, left). The same tendency was observed when JIGs were juxtaposed to 299 driver genes and mutations identified from a comprehensive PanCancer and PanSoftware analysis spanning 9,423 tumor exomes by employing 26 computational tools (Bailey et al., 2018). JIGs represent 8.9% (27 genes) of the identified cancer drivers versus 1.0 ± 0.6% of random genes (*p* < 0.001) (Figure 4A, middle). Similar results were obtained when the same analysis was performed versus a group of 127 significantly mutated genes which have been identified as oncogenesis drivers across 12 major cancer types, whereby most tumors bear two to six of these mutations (Kandoth et al., 2013). A significant enrichment of JIGs for 18 of these genes (5.9% compared to expected 0.6%, which is 127 in 19,313 coding genes) was observed, as opposed to the random genes (0.5 ± 0.4%, *p* < 0.001) (Figure 4A, right). Overall, compared to the control lists, JIGs are highly enriched in cancer driver genes and mutations (Figure 4A and Supplementary Table 3). Then, using gene mutation data from PanCan cohort, we calculated the mutation frequency for all JIGs across human cancer types, and found a significant increase compared to control lists (Figure 4B). To further assess whether JIGs tend to be epigenetically altered in cancer, we meta-analyzed data of the DiseaseMeth 2.0 database (Figure 4C), and found frequent alterations of DNA methylation in JIGs, while hypomethylation was the most prevalent type of aberration in tumors versus normal tissue controls (131 JIGs hypomethylated, 47 hypermethylated, 47 hypo-/hyper-methylated, χ^2^ = 62.72, *p* < 0.001). Collectively, JIGs appear to undergo mutations and/or perturbations of DNA methylation patterns in cancer.

### JIGs Are Transcriptionally Deregulated in Aggressive Stages and Predict Cancer Patient Outcomes

**FIGURE 5:**
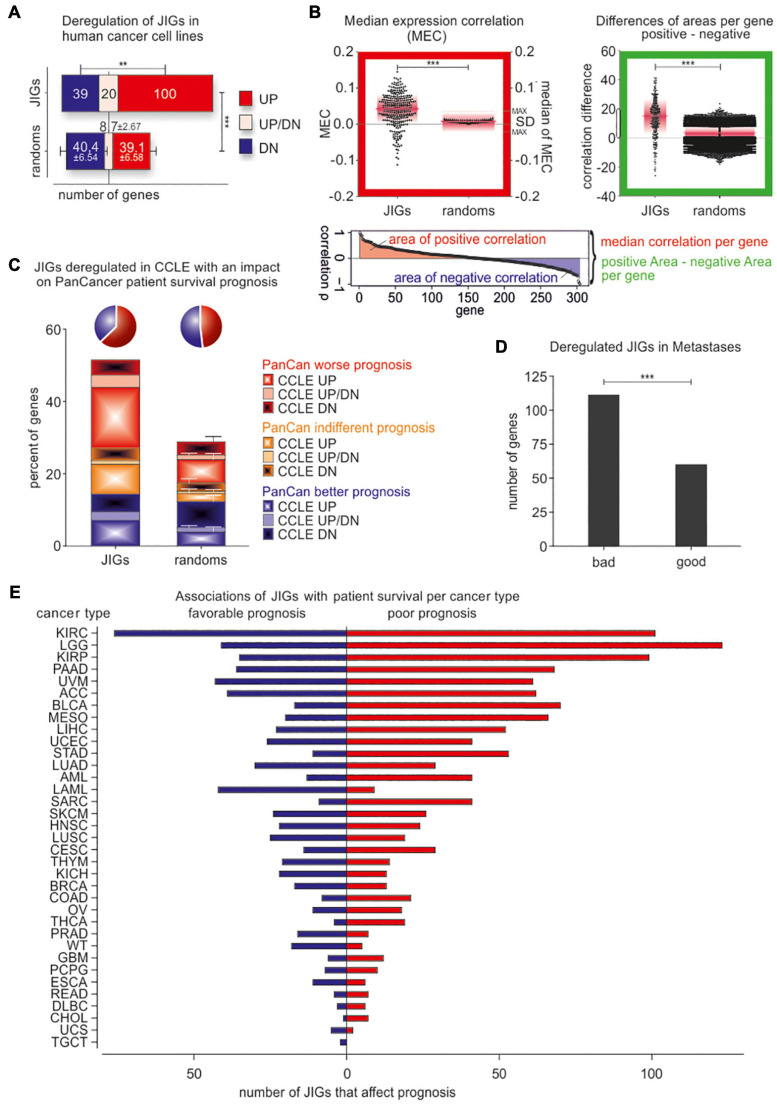
JIGs are transcriptionally deregulated in invasive/metastatic stages and predict clinical outcomes in a cancer type-dependent manner. **(A)** JIG transcripts are deregulated in the highly invasive versus the less-invasive CCLE cell lines, and the total percentage is 1.8-fold higher than the corresponding percentage of control lists. Red: upregulated, blue: downregulated, pink: cancer type-dependent deregulation. Error bars represent mean with SD. **(B)** *Red-boxed diagram:* median expression correlation (MEC) in PanCan and SD of MEC (pink boxes) are higher among JIGs compared to random lists (shown are the 100 medians of MEC with maximal SD). *Green-boxed diagram:* the differences of positive versus negative area of correlation per gene show (a) a significantly larger span and (b) over all a higher positive value compared to 100 random gene lists. Pink lines represent the median, pink boxes represent SD. **(C)** Cox regression analysis of JIGs on the survival of the TCGA PanCan cohort. 227 JIGs correlated with patient outcomes, of which 141 predicted worse and 86 better outcomes. Among 159 deregulated JIGs in highly-aggressive states across several cancer types of CCLE, 118 JIGs (74.2%) influence patient prognosis. Of those, 74 (62.7%) (pie chart, blue) had an adverse effect, 51 were exclusively upregulated in aggressive states of CCLE (CCLE UP, plain red), while additional 10 JIGs were upregulated in some as well as downregulated in other cancer types (CCLE UP/DN, plain pink). Only 44 JIGs differentially expressed in CCLE (dashed blue, plain blue, and dashed pink) had a beneficial effect on survival prognosis (pie chart, green). **(D)** Transcriptional deregulation of JIGs in metastases versus primary tumor or normal tissue, and correlation with JIGs that affect patient prognosis in PanCan. JIG deregulations in 6 metastatic cancer types are more correlated with poor outcomes (1.85:1). **(E)** Survival analysis for JIGs across different TCGA cancers. The prognostic potential of JIGs depends on the cancer type. In the majority of cancer types, more JIGs are associated with poor prognosis and less with favorable prognosis. No cancer tissue was found unaffected by JIGs.

To further explore potential links between jaw development and cancer progression, we checked invasive cancer cell lines and patient tumors for changes in JIG transcriptional activity. In this regard, based on a recently described approach (Logotheti et al., 2020), we classified all cell lines of the Cancer Cell Line Encyclopedia (CCLE; Barretina et al., 2012), which includes gene expression data of 962 cell lines, into highly-invasive and less-invasive types, according to the levels of E-cadherin, N-cadherin, Vimentin, ZEB1, and SNAI1, which constitute reliable markers for EMT and tumor progression (Khan et al., 2017). Then, we examined whether JIGs are differentially expressed in highly- versus less-invasive cells across 24 common cancer types. We found that, compared to the control lists, a significantly higher number of JIGs is differentially expressed in high- versus low-aggressive cells (1.8-fold higher, *z* = 8.89, *p* < 0.001), where more JIGs are upregulated (JIGs vs. control: 100 vs. 39.1 ± 6.6 genes) than downregulated (39 vs. 40.4 ± 6.5 genes, *p* < 0.003, Figure 5A and Supplementary Table 3). These results indicate a non-stochastic tendency for enhanced transcription of JIGs in highly invasive states.

Additionally, to evaluate the clinical relevance of these findings, we meta-analyzed gene expression data in 35 different cancer types from PanCan TCGA cohort (Liu et al., 2018). Interestingly, correlation analyses implied that a large fraction of JIGs are co-expressed in patient tumors, with a tendency to preserve their crosstalks. In particular, relative to the control lists JIGs demonstrated increased value of (i) median expression correlation (MEC) per gene (JIG vs. control: 0.039 ± 0.045 vs. 0.008 ± 0.003, *z* = 6.99, *p* < 0.001, Figure 5B, red-boxed diagram), and (ii) more and greater positive correlation areas per gene (13.72 ± 13.05 vs. 3.04 ± 5.39, *z* = 33.6, *p* < 0.001, Figure 5B, green-boxed diagram). Using Cox regression, we deciphered all genes associated with patient survival, and subsequently determined the percentage of significant prognostic factors among JIGs. Overall, 227 JIGs correlated with patient outcomes, and showed a higher ratio of poor versus favorable prognostic factors (1.64:1, χ^2^ = 13.33, *p* < 0.001) compared to the corresponding ratio of the control lists (1.25:1, χ^2^ = 2.79, *p* = 0.095, correspondingly). We also found that 118 JIGs are both, deregulated in highly aggressive states in CCLE and correlated with patient outcomes in PanCan, with a ratio of 1.68:1 for poor versus favorable prognostic factors (χ^2^ = 7.63, *p* < 0.006) as opposed to the corresponding 0.93:1 ratio of the control lists (χ^2^ = 0.1, *p* = 0.755, Figure 5C and Supplementary Table 3). Collectively, JIGs appear to become deterministically deregulated in highly invasive cells, with frequent transactivation events, and predict poor patient outcomes. Similar alterations in JIGs also occur in metastatic lesions. In particular, we additionally compared these data with transcriptomes of primary versus metastatic lesions from breast, colon, hepatocellular, medulloblastoma, melanoma, and prostate cancers, that were retrieved from the GEO database (Clough and Barrett, 2016). We found that more JIGs that were poor prognostic factors in PanCan cohort are deregulated in metastases, as opposed to the corresponding favorable factors (1.85:1, χ^2^ = 15.21, *p* < 0.001, Figure 5D and Supplementary Table 3).

Next, we estimated the effect of each JIG on prognosis for individual cancer types, using Cox regression (Figure 5E, Table 1, and Supplementary Table 4). We found that all JIGs correlate with patient outcomes and that a single JIG can affect prognosis of at least three cancer types. Although there were JIGs implicated in as many as 16 cancer types, no single JIG was universally associated with prognosis in all 35 cancers. Rather, their effect is cancer type-dependent. The top-ten most impacted cancer types, ranked by the total number of JIGs affecting, either beneficially (left) or detrimentally (right), the disease outcome are KIRC, LGG, KIRP, PAAD, UVM, ACC, MESO, BLCA, LIHC, and STAD.

**TABLE 1:** Effects of JIGs on cancer patient prognosis (in descending order of total number of JIGs that affect each cancer type).

Abbreviation	Cancer type	Number of JIGs associated with poor prognosis	Number of JIGs associated with favorable prognosis
KIRC	Kidney renal clear cell carcinoma	115	87
LGG	Brain Lower Grade Glioma	130	49
KIRP	Kidney renal papillary cell carcinoma	112	46
PAAD	Pancreatic adenocarcinoma	89	47
UVM	Uveal Melanoma	75	52
ACC	Adrenocortical carcinoma	77	49
MESO	Mesothelioma	88	25
BLCA	Bladder Urothelial Carcinoma	89	21
LIHC	Liver hepatocellular carcinoma	75	29
STAD	Stomach adenocarcinoma	72	21
UCEC	Uterine Corpus Endometrial Carcinoma	54	33
LUAD	Lung adenocarcinoma	46	40
AML	Acute Myeloid Leukemia	60	23
SKCM	Skin Cutaneous Melanoma	42	39
LAML	Acute Myeloid Leukemia	18	58
LUSC	Lung squamous cell carcinoma	40	34
SARC	Sarcoma	55	17
KICH	Kidney Chromophobe	34	35
HNSC	Head and Neck squamous cell carcinoma	34	33
CESC	Cervical squamous cell carcinoma and endocervical adenocarcinoma	39	25
BRCA	Breast invasive carcinoma	26	32
THYM	Thymoma	19	33
OV	Ovarian serous cystadenocarcinoma	31	19
THCA	Thyroid carcinoma	41	9
COAD	Colon adenocarcinoma	33	16
ESCA	Esophageal carcinoma	15	25
GBM	Glioblastoma multiforme	24	15
PCPG	Pheochromocytoma and Paraganglioma	21	16
PRAD	Prostate adenocarcinoma	10	26
WT	Wilms Tumor	7	29
READ	Rectum adenocarcinoma	24	8
DLBC	Lymphoid Neoplasm Diffuse Large B-cell Lymphoma	14	11
CHOL	Cholangiocarcinoma	17	3
UCS	Uterine Carcinosarcoma	7	11
TGCT	Testicular Germ Cell Tumors	1	5

### Gnathostome-Specific JIGs Are Preferably Deregulated During Cancer Progression

The developmental origin of the cartilaginous jaw consists a turning point in vertebrate evolutionary history and is attributed to the neural crest (Sauka-Spengler and Bronner-Fraser, 2008), and especially to the cranial neural crest cells, from which cartilage is exclusively formed (Martik and Bronner, 2017). Evolutionary Developmental (Evo-Devo) biology studies comparing embryonic programs in jawless versus early jawed chordates indicate that instead of appearing *de novo*, jaws have rather arisen through the co-option of an ancient developmental pre-pattern (Cerny et al., 2010), in association with corresponding changes in the underlying GRNs. Jaw evolution was driven by incorporation of new genes into a pre-existing dorso-ventral patterning program, which altered the identity of jaw-forming chondrocytes (Cerny et al., 2010). For instance, some transcription factors are components of neural crest GRNs in both, jawed and jawless vertebrates, while other transcription factors are cranial neural crest-specific and included in gnathostomes, but missing from lampreys’ GRNs (Martik and Bronner, 2017).

**FIGURE 6:**
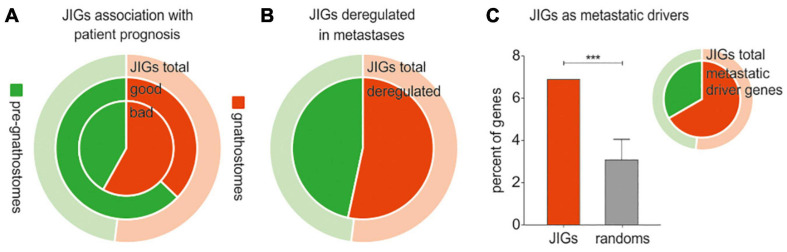
Cancer progression and metastasis are associated with deregulation of evolutionarily younger JIGs. Red indicates gnathostome-specific, green indicates pre-gnathostome orthologs. In all pie charts, the outer, ring represents the composition of the total 305 JIGs in gnathostome-specific (light red) and pre-gnathostome (light green) JIGs. **(A)** Cox regression analysis of JIGs in PanCan revealed that 51.6% of gnathostome-specific orthologs predict worse and only 20.1% better prognosis (ratio 2.56:1) versus 40.4 and 37.0% (ratio 1.09:1) in pre-gnathostome genes. **(B)** In metastasis, a slightly higher number of gnathostome-specific JIGs is deregulated compared to the pre-gnathostomes (inner circle). **(C)** Estimation of gnathostome versus pre-gnathostome genes that are deregulated in a setting of xenograft experimental evolution. Diagram: among the 700 genes which were identified as drivers of tumor evolution, significant enrichment of JIGs was found (6.89%, or 21 genes) as compared to random lists (3.08 ± 0.98%). Pie: of the 21 deregulated JIGs, 14 (66.7%) were of gnathostome and 7 (33.4%) of pre-gnathostome origin.

According to the abovementioned Evo-Devo concepts, the JDN components do not have a similar evolutionary age. It is rather plausible that gene homologs which arose in gnathostomes might have got interconnected with a network that pre-existed in agnatha, perhaps to support morphological novelty. Motivated by this, we questioned whether invasive cancer cells show preference to “usurp” the pre-existing genes or the ones that were incorporated to the JDN after the divergence of jawed vertebrates from cyclostomes. In this respect, we approximated the evolutionary age of all JIGs (Supplementary Table 5) and assessed the effects of pre-gnathostome versus gnathostome-specific orthologs on cancer progression. Importantly, to eliminate bias in the estimation of the JIGs’ evolutionary age from partially sequenced animal genomes, we included only model organisms with well-annotated genomes. We found that of 305 JIGs, 159 JIGs probably originated in jawed vertebrates, since no “true orthologs” of their corresponding proteins could be detected in jawless species, whereas the other 146 JIGs are of pre-gnathostome origin. Cox regression analysis in PanCan revealed that a higher number of gnathostome-specific JIGs is associated with poor patient outcomes, as opposed to the pre-gnathostome JIGs with an almost equal distribution (82:32 vs. 59:54, χ^2^ = 8.56, *p* < 0.002, Figure 6A). Consistently, a tendency toward transcriptional deregulation of gnathostome-specific versus pre-gnathostome JIGs was observed in metastases compared to primary tumors (χ^2^ = 1.09, *p* < 0.296, Figure 6B). We also performed a similar meta-analysis on data produced in a mouse experimental setting, where a tumor’s evolution from initiation to metastasis has been simulated *in vivo* via sequential xenografting (Chen et al., 2015). We estimated gnathostome versus pre-gnathostome genes that are deregulated in a setting of xenograft experimental evolution, where a tumor’s full-life history from initiation to metastasis was simulated by transforming the immortalized human breast epithelial cell line with *HRAS* and performing sequential xenografting in mice until metastases were observed (Chen et al., 2015). Among the 700 genes which underwent driver expression changes, on the basis that their expression was exclusively increasing or decreasing (Chen et al., 2015), we found that JIGs are significantly enriched (*z* = 3.9, *p* < 0.001), while their majority tends to be of gnathostome rather than pre-gnathostome origin (14:7, χ^2^ = 1.71, *p* = 0.19, Figure 6C and Supplementary Table 3). Collectively, the strong correlations of gnathostome-specific genes with poor and metastatic outcomes, as well as with drivers of experimental tumor evolution underscore a preference for deregulation of evolutionarily younger JIGs toward tumor progression.

### Deregulated Expression of JDN Components During Cancer Progression Is More Pronounced in the Gnathostome-Specific Hubs

**FIGURE 7:**
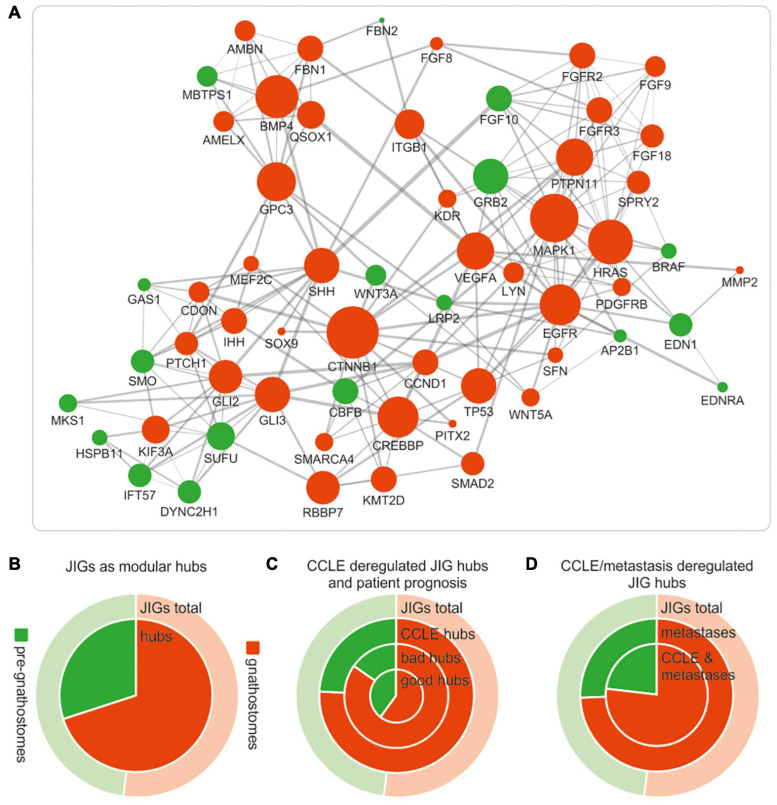
Deregulations of gnathostome-specific hubs of the JDN are more frequent associated with invasive and metastatic stages, as opposed to pre-gnathostome hubs. **(A)** Functional interaction network (confidence interaction score ≥ 0.9) of the gnathostome-specific and pre-gnathostome JIG hubs. The size of the nodes is proportional to the node degree in the “original” JDN network in Figure 3d. The 60 nodes that represent intra- and inter-modular hubs in the original network appear to be interconnected and form a rather dense network. **(B)** The gnathostome-specific genes make up 52% (159) of all 305 JIGs (outer ring “JIG total,” red), whilst the proportion of gnathostome hubs is significantly enriched in relation to the pre-gnathostome (inner circle “hubs,” red). **(C)** More gnathostome-specific hubs that are deregulated in CCLE cells are correlated with worse prognosis in PanCan cohort (ring “bad hubs,” red). In contrast, more pre-gnathostome hubs tend to correlate with favorable than poor prognosis (compare ring “bad hubs” with inner circle “good hubs,” green). **(D)** More gnathostome-specific hubs are found deregulated, compared to the pre-gnathostome ones, in metastatic lesions (inner ring). This preference is more pronounced for hubs commonly deregulated in metastatic samples and CCLE cells (inner circle).

JIGs appear to form a highly interconnected network (Figure 3D). In biological networks, the most highly connected nodes, the so-called “hubs,” are considered biologically significant and more relevant to the overall function of the network (Barabási et al., 2011; Kontou et al., 2016; Pavlopoulos et al., 2018). The intra-modular hubs are central to a given network module, with the highest number of connections to the neighboring nodes, whereas inter-modular hubs are intermediate between two or more modules. Taking this into account, we sought to investigate whether gnathostome-specific genes that are deregulated in cancer also occupy hub positions in the JDN. First we identified, through STRING network analysis, 60 nodes representing intra- and inter-modular hubs (Figure 7A). By approximating their evolutionary age, we found that these hubs correspond largely to gnathostome-specific versus pre-gnathostome orthologs (Figure 7B and Supplementary Table 5). Upon comparison of these 60 hubs to CCLE-derived data, we found that 33 (55.0%) of them are deregulated in highly invasive cancer cells, 25 of which are of gnathostome-, and 8 of pre-gnathostome origin (χ^2^ = 7.76, *p* = 0.005).

Notably, all 33 JDN hubs predicted by this approach are indeed causatively linked with invasiveness and/or metastasis in a cancer type-dependent manner, according to experimental evidence in the preclinical setting (Supplementary Table 6). In particular, studies using *in vitro* cell lines, mouse xenografts and/or patient samples underscore that most of the hubs promote tumor progression and metastasis. Further data curation in the clinical trials database showed that for several of these hubs, molecular modifiers have been developed and entered clinical trials, either as monotherapies or as combination regimens. In addition, screenings in the drugs.com online pharmaceutical encyclopedia and the publicly available database of the United States Food and Drug Administration (FDA) indicated that small molecules targeting six of these hubs are already approved and marketed drugs for the treatment of aggressive and metastatic cancers. Indeed, as shown in Supplementary Table 6, our model was able to identify EGFR which is targeted by approved drugs such as cetuximab, dacomitinib and erlotinib; members of the VEGF (VEGFA/KDR) pathway which are targeted by, for example, bevacizumab, pazopanib or sorafenib; members of the FGFR family of receptors, which are targeted by erdafitinib or pemigatinib; and effectors of the hedgehog signaling pathway (SMO, SHH) which are targeted by glasdegib, sonidegib or vismodegib. Other factors, such as GLI2, GLI3, CDON, MEF2C, PITX, SFN, SPRY2, WNT3A and WNT5A (Supplementary Table 6), show a consistent metastasis-promoting role across several cancer types in preclinical studies, which provides a foundation for the development of molecular modifiers against them and their introduction to clinical trials.

Regarding the age of the hubs of JDN in relation with tumor invasiveness, our studies indicate that among the 60 JDN hubs, the evolutionarily younger ones tend to be more frequently deregulated in aggressive states. The tendency for deregulation of gnathostome-specific hubs as compared to the pre-gnathostome hubs is clinically relevant for cancer patients. We found that more gnathostome-specific hubs are correlated with worse than favorable prognosis (17:10 hubs, correspondingly), whereas the opposite applies for the pre-gnathostome hubs (4:9, χ^2^ = 2.47, *p* = 0.116, Figure 7C). In addition, aberrant transcription of more gnathostome-specific than pre-gnathostome hub JIGs was observed in metastatic versus primary tumors (35:12 = 2.92, χ^2^ = 11.26, *p* < 0.001, Figure 7D). This effect is particularly observed among those hubs that are deregulated in highly invasive cancer cell lines (25:8 = 3.13, χ^2^ = 8.76, *p* = 0.003, Figure 7D). Overall, there is a preference for transcriptional deregulation of gnathostome-specific hubs in the invasive and metastatic stages. Taken together, these results indicate a prevalence of gnathostome-specific genes in occupying hub positions in JDN. However, at the same time, these are preferentially exploited by cancer cells over pre-gnathostome hubs to promote cancer aggressiveness.

## Discussion

Cancer is considered an evolutionary and ecological process (Merlo et al., 2006). A neoplasm consists of genetically and epigenetically heterogeneous cell populations that compete for space and resources, evade immune surveillance and cooperate to disseminate to secondary organs. The fitness of cell subpopulations is further shaped by their interactions with cellular and molecular components of the tumor microenvironment. The fittest, or “evolutionarily successful,” cell variants are those acquiring capabilities which increase the probability to obtain metastatic potential (Merlo et al., 2006). Darwinian laws apply to both, the tumors and the organisms on which tumors grow. Somatic selection occurs along with organismal selection, following “a mirror within a mirror”-like pattern: heterogeneous cell subpopulations of a growing tumor undergo selection of the fittest within a population of organisms which is under constant evolutionary pressure. Although the timeframe for clonal selection of tumor cells (in months or years) is significantly more narrow than for species selection (millions of years), it is reasonable to envisage that, at a given evolutionary time point, the attributes encoded in the genome of a specific species can be accessed by its cancerous tumor.

Herein, we provide compelling evidence that metastasis is phenotypically manifested within the gnathostome clade, and that genes which are essential for jaw development, a hallmark macroevolutionary trait of gnathostomes, are co-opted during cancer progression. Genes supporting jaw developmental programs tend to undergo mutational and epigenetic changes, with frequent transactivations in invasive and metastatic stages, and a preference for enhanced transcription of the gnathostome-specific versus the ancient ones. These data strongly suggest that the same genes/gene interactions underlying key innovations are also preferentially co-opted within the tumor context toward aggressive outcomes. Certain structures which provide selective advantages at an organismal level, such as placenta development (Costanzo et al., 2018), neural crest formation (Kerosuo and Bronner-Fraser, 2012), and jaw development (this study) may be “hacked” by cancer cells to improve their own fitness, manifested as metastatic potential. The preference of invasive cancer cells to usurp evolutionarily newer hubs of the JDN further reinforces this notion. This observation also implies that there might be a trade-off between the vulnerability to metastasis and the conservation of key innovations that are indispensable for vertebrate fitness and, thus, cannot undergo secondary losses.

Successful prediction of the likely paths of tumor progression is valuable for diagnostic, prognostic, and treatment purposes, but effective models are still not in place (Diaz-Uriarte and Vasallo, 2019). For establishing such prediction models and designing drugs that target events of tumor evolution (Amirouchene-Angelozzi et al., 2017), it is essential to unveil parallels between organismal and clonal selection. Based on our findings, we introduce a systems-based, key innovation-driven model, as an *in silico* tool for prediction of putative prometastatic drivers, on the rationale that genes that are crucial for evolution of a species might be important for tumor evolution. The fact that the results derived using this model are in agreement with the bulk of preclinical studies and with clinical interventions (see Supplementary Table 6 for details) underscores both, the prediction accuracy of this approach and the translational value of these candidates. In particular, our computational model predicted 33 hub JIGs that are deregulated in highly invasive cell lines of CCLE, consistently with experimental *in vitro*, *in vivo* and/or in patient evidence that these genes mainly support cancer progression. Several of these gene targets have already been translated to marketed drugs, while other factors predicted by this model might represent promising novel targets, since they show consistent metastasis-inducing effects across several cancers. New molecular entities able to inhibit these candidates can be developed and be further assessed in the clinical setting for their potential to prevent metastatic progression or disease recurrence. Out of these 33 hubs, 25 are of gathostome- versus 8 of pregnathostome-origin, indicating a pronounced tendency of cancer cells to usurp the evolutionarily-younger hubs of the JDN network in order to evolve to aggressive stages. Hence, taking into account that a tumor may progress by usurping genes specifically related with vertebrate key-innovations, this model may hold a potential to facilitate the prediction of tumor evolutionary trajectories.

A main future challenge is to design comprehensive experimental settings, where associations between key innovations and metastasis could be investigated at the mechanistic and molecular level, in parallel with corresponding cancer phenotypes. Studies in primitive metastasis-competent taxa versus the incompetent ones could facilitate reconstruction of the evolutionary history of metastasis by identifying which molecular events that catalyzed gnathostome evolution have also consistently benefited the evolution of primary tumors toward more aggressive stages. Advantageously, a well-established Evo-Devo study system of gnathostomes versus pre-gnathostomes, amenable to experimental and genetic manipulations at embryonic stages (Kuratani et al., 2002; Cerny et al., 2010; Jandzik et al., 2015), could be repurposed for cancer research. This *in vivo* system is comprised of three chordata species of the same evolutionary lineage, presenting progressively aggressive cancer phenotypes from the most ancient to the most recent: amphioxus, the most basal extant chordate, with benign tumors; the metastasis-incompetent jawless lampreys; and the metastasis-competent jawed zebrafish. By comparing lamprey and amphioxus development with that of zebrafish, and other vertebrates like frog and salamander, Evo-Devo specialists attempt to reconstruct genetic and developmental changes underlying the major events in vertebrate evolution (Kuratani et al., 2002; Cerny et al., 2010; Jandzik et al., 2015). Subjecting this system into carcinogenic treatments and comparatively examining the developing lesions in conjunction with genetic and functional changes might provide a glimpse into conserved pathways which are consistently recapitulated in metastases. For instance, it could be investigated whether absence of certain gnathostome-specific genes/gene interactions in the jawless organisms hinders activation of prometastatic cascades in their respective tumors, as opposed to their jawed counterparts. Moreover, studies on cancer-resistant pre-vertebrates, such as echinoderms, urochordates and cephalochordates, could also be considered to dissect their cancer resilience in relation with their hallmark attributes, for instance their increased regeneration ability (Somorjai et al., 2012).

Our key innovation-driven model underscores a preference of invasive tumors for usurping evolutionarily newer features. Otherwise, a recent concept, known as “atavistic model,” asserts that neoplasms rely on re-expression of ancestral traits and reverse evolution from multicellularity (MC) to unicellularity (UC; Bussey et al., 2017), which is likely promoted by upregulation of UC genes, disruption of interconnectedness between UC and MC genes (Trigos et al., 2017) and loss-of-function mutations on MC genes (Chen et al., 2015). Furthermore, recent studies show that metastatic competence arises from heterogeneous cancer cell populations without the need for acquisition of additional mutations, and is benefited from further selection of tumor-initiating mutations that seed primary tumorigenesis (Jacob et al., 2015). By unifying these notions, we propose that tumor initiation may be triggered by mutations in evolutionarily old genes governing processes at the “dawn” of multicellularity, such as cell proliferation and genomic stability (Lineweaver et al., 2014). Such alterations in ancient genes may confer genetic heterogeneity (Chen et al., 2015), which is the driving force of evolution. Subsequently, tumor progression may be enabled via selection of clones that entail crosstalks between tumor-initiating mutations already acquired at primary tumor cells and evolutionarily-young genes that support gnathostome key-innovations. Future studies could uncover if such crosstalks guide recurrent routes to metastasis, thereby providing a foundation for the rational design of strategies that prevent cancer evolution.

## Data Availability Statement

The datasets presented in this study can be found in online repositories. The names of the repository/repositories and accession number(s) can be found in the article/Supplementary Material.

## Author Contributions

SL, SM, and BP conceived the project and designed experiments. SM developed bioinformatics pipelines and analyzed high-throughput data. AP and IT designed and performed phylogenomics and network analyses. PD and SL performed literature research. SL, SM, and AP analyzed and interpreted data. IT and SM prepared the figures. SL drafted the manuscript. SL, AP, and BP reviewed and edited the manuscript. All authors read and approved the final manuscript.

## Conflict of Interest

The authors declare that the research was conducted in the absence of any commercial or financial relationships that could be construed as a potential conflict of interest.

## Supplementary Material

Supplementary Figure 1 Maximum likelihood-based tree of protein sequences of the p53 family. A characteristic example of the well-studied *p53* gene family, where distinct orthologs of the three members of this family (i.e., TP53, TP63 and TP73) were found exclusively in gnathostome species. The agnathan p53 homologs occupy a basal position in this phylogenetic tree, suggesting that a primordial *TP53/63/73* gene might have existed in jawless species which after a series of duplications gave rise to the functionally divergent *TP53*, *TP63* and *TP73* genes in jaw-bearing vertebrates.

Supplementary Table 1 Taxa under study in correlation with their tumor characteristics, their circulatory and immune systems, the level of body organization and their ability to develop metastatic or lethal cancers.

Supplementary Table 2 Mouse gene knockouts which present jaws abnormalities. The human orthologs are included. The ratio of the jaw-related phenotypes to all developmental phenotypes affected by each gene knockout is also shown.

Supplementary Table 3 Associations of each JIG with genetic and epigenetic alterations in tumors, survival of PanCan patients, and expression in highly invasive or metastatic stages. Column annotations: CGC = included in the Cancer Gene Census list, Cancer driver = included among the cancer driver genes in PanCan, freqmut = included among the frequently mutated genes in PanCan, Methylation = differentially methylated JIGs in human tumors versus normal tissue, from DiseaseMeth database (hypo = hypomethylated, hyper = hypermethylated), CCLE = up- or down-regulated in highly-invasive vs. less-invasive human cells from the CCLE, Survival = impact on the survival prognosis of the patients from the PanCancer cohort based on Cox regression analysis, hsa_Metastases = up- or down-regulated in human metastatic vs. primary lesions (GSE21510, GSE2509, GSE25976, GSE43837, GSE468, GSE6919, GSE7929, GSE7930, GSE8401), mmu_metast_driver = included among metastasis driver genes in a mouse model of tumor evolution.

Supplementary Table 4 JIGs and their effect on patient survival prognosis across different cancer types of TCGA based on Cox regression analysis. Annotations: poor prognosis = 1, favorable prognosis = -1, insignificant = 0.

Supplementary Table 5 JIG orthologs across pre-gnathostome and gnathostome species. Annotations: green = pregnathostome orthologs, orange: gnathostome-specific orthologs, underlined = network hubs.

Supplementary Table 6 Comprehensive overview of the preclinical and clinical research studies on the hub JIGs that are deregulated in highly-invasive cell lines of CCLE. The drivers of tumor progression predicted using the key innovation-driven model are in agreement with existing experimental data.
