# Supplementary material for: A Systems-Based Key Innovation-Driven Approach Infers Co-option of Jaw Developmental Programs During Cancer Progression
Source: Front Cell Dev Biol. 2021 Jun 2;9:682619. doi: 10.3389/fcell.2021.682619 (PMC8207138; doi:10.3389/fcell.2021.682619)
Supplement: Supplementary Figure 1 — Maximum likelihood-based tree of protein sequences of the p53 family. A characteristic example of the well-studied p53 gene family, where distinct orthologs of the three members of this family (i.e., TP53, TP63 and TP73) were found exclusively in gnathostome species. The agnathan p53 homologs occupy a basal position in this phylogenetic tree, suggesting that a primordial TP53/63/73 gene might have existed in jawless species which after a series of duplications gave rise to the functionally divergent TP53, TP63 and TP73 genes in jaw-bearing vertebrates. [file Data_Sheet_1.PDF]

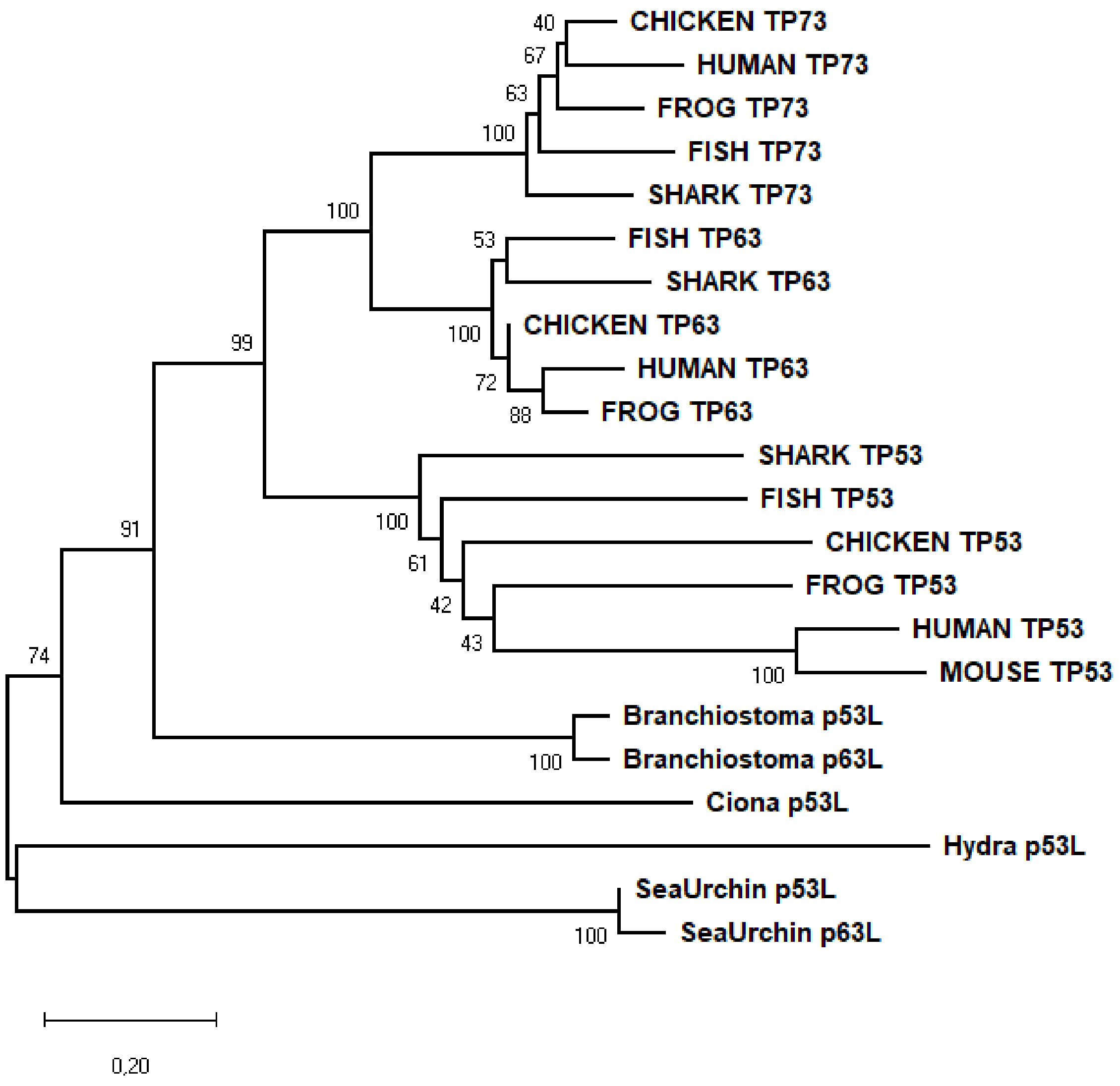

S1 Fig.: Maximum likelihood-based tree of protein sequences of the p53 family. A characteristic example of the well-studied *p53* gene family, where distinct orthologues of the three members of this family (i.e., TP53, TP63 and TP73) were found exclusively in gnathostome species. The agnathan p53 homologs occupy a basal position in this phylogenetic tree, suggesting that a primordial *TP53/63/73* gene might have existed in jawless species which after a series of duplications gave rise to the functionally divergent *TP53*, *TP63* and *TP73* genes in jaw-bearing vertebrates.

Supplementary Table 1: Taxa under study in correlation with their tumor characteristics, their circulatory and immune systems, the level of body organization and their ability to develop metastatic or lethal cancers.

TAXON	LEVEL OF BODY ORGANIZATION	EVOLUTIONARY HALLMARK FIRST DEVELOPED IN THIS TAXON	CIRCULATORY SYSTEM	IMMUNE DEFENSE	TUMOR CHARACTERISTICS	METASTATIC/ LETHAL TUMORS
**Porifera**	Cellular	Multicellularity	no circulatory system	innate immunity	No apparent tumor lesions	NO
**Cnidarians**	Cell-Tissue	Tissues	no circulatory system	innate immunity	Tumors in two Hydra species (*Hydra oligactis* and *Pelmatohydra robusta*) are composed of intersistial cells that are morphologically similar to female germline precursor cells. Tumor cells cannot terminally differentiate, do not show increase in proliferation activity and are not removed by apoptosis. They show autonomous growth, insensitivity to environmental stimuli and increased migratory potential. Although tumors impact fitness, causing reduced capacity for egg production and reduced population growth rate, they are not lethal for the affected Hydra polyps	NO
**Platyhelminthes**	Tissue-Organ	Bilateral symmetry, triploblastic (endoderm-mesoderm-exoderm)	no circulatory system	innate immunity	Spontaneous tumors in planarians (*Dugesia tigrina* , *D. etrusca* and *D. ilvana*). In Trematode, one case of ganglioneuroblastoma is reported in RTLA. In plannarian, 6 cases of parenchyma polyps and 7 cases of epidermal papilloma have been reported in RTLA.	NO
**Nemerteans**	Organ/system organ	circulatory system, complete digestive system	open circulatory system	innate immunity	nd	nd
**Nematodes**	Organ/System organ	Body cavity	no circulatory system	innate immunity	Germline cell-derived tumors, confined in the gonads. Tumor cells are mitotically active and fail to differentiate to gametes. Excessive proliferation leads to severe swelling of the proximal gonad and content release	NO
**Molluscs**	Organ/System organ	Coelom	open circulatory system	innate immunity	In Gatrsopoda, Bivalvia and Cephalopoda, there are reports for (a) disseminated neoplasia, where cells in the hemolymph are amplified, disseminate through the circulatory system and infiltrate the connective tissue of various organs; and (b) gonadal neoplasia, which consists of an abnormal proliferation of undifferentiated germinal cells. They are characterized by undifferentiated cells, rapid and invasive growth, aberrant chromosome numbers and progressive development, which is often fatal for the affected individuals. Additionally, horizontally transmissible DN has in the populations of Mya arenaria in New York, Maine and Prince Edward Island, Canada	YES
**Echinoderma**	Organ/System organ	Deuterostome development, endoskeleton	Main circulation of coelomic fluids by peritoneal cilia	innate immunity	No reported spontaneous tumors in the Registry of Tumors in Lower Animals. No tumor formation after treatment of post-embryonic stage sea urchins with chemical carcinogens under laboratory condition. Pigmented tumor-like lesions, at a percentage of 7.4%, have been reported in a population of 95 Ophiuroids collected between 1954-1955 in Plymouth, England. These lesions consisted of masses of dedifferentiated cells and were designated as early, intermediate and late stages of development, but were non-invasive and non-lethal	NO
**Cephalochordates**	Organ/System organ	Notochord, dorsal tubular nerve cord, pharyngeal pouches, endostyle, postnatal tail	closed circulatory system. Ventral heart, with dorsal and ventral blood vessels	innate immunity	A single report of neoplasm in Branchiostoma lancelotus, a chromaffinoma similar to the same neoplasm in humans	NO
**Urochordates**	Organ/System organ	Notochord, dorsal tubular nerve cord, pharyngeal pouches, endostyle, postnatal tail	closed circulatory system. Dorsal and ventral blood vessels. No heart	innate immunity	No actual spontaneous tumors have been recorded	NO

**Cyclostomes**	Organ/system organ	Neural crest	closed circulatory system, 2-chambered heart	innate and adaptive immunity	Hepatomas, liver hamartomas, and hyperplastic nodules. Mesenchymal tumors of the skin, epithelial neoplasms. Tumors exhibit low and high differentiation characteristics, but there is no apparent metastasis	NO
**Chondrichthyes**	Organ/System organ	cartilaginous jaws	closed circulatory system, 2-chambered heart	innate and adaptive immunity	Cholangiocarcinoma, Chondroma of lumbar vertebrae, Chondroma-vertebral, Choroid plexus papilloma, Chromaffinoma, Cutaneous chondroma, Cutaneous epithelioma, Cutaneous fibroma, Cutaneous fibrosarcoma, Cutaneous hemangioma, Cutaneous melanoma, Cutaneous melanoma-invasive, Cutaneous melanoma-metastatic, Cutaneous myxofibroma, Cutaneous odontoma, Cutaneous osteoma, Enteric adenoma/carcinoma, Epidermal papilloma, Fibroepithelial lip polyp, Fibroma, Hepatic capsular fibroma, Hepatocellular adenoma, Hypodermal lipoma, Lymphoma, metastatic adenocarcinomas, mesothelioma, Mucoepidermoid papilloma of the maxillary gingiva, Neurofibroma, Olfactory neuroblastoma, Renal carcinoma, Seminoma, Subcutaneous fibrous hemangioma, Thyroid carcinoma	YES
**Osteichthyes**	Organ/System organ	bony endoskeleton	closed circulatory system, 2-chambered heart	innate and adaptive immunity (Cooper MD 2006)	branchial, cardiovascular, cutaneous, endocrine, gonadal, hepatobiliary and pancreatic, lymphohematopoietic, mesenchymal, neoplasms of embryonal origin, neoplasms of the alimentary track, neoplasms of the nervous system, ocular, renal. Malignant neoplasms are characterized ny local invasion. Distant metastasis is rare, however it has been reported for this animal group	YES
**Amphibians**	Organ/System organ	lungs, tetrapod leg	closed circulatory system, double circulation, 3-chambered heart	innate and adaptive immunity (Cooper MD 2006)	Epidermal papillomas, cutaneous hyperplasia, dermal gland tumors, gastric adenocarcinoma, granulocytic leukemia, hepatocellular carcinoma, intestinal adenocarcinoma, Lucke´ renal adenocarcinoma, lymphoma, mast cell tumors, melanophoromas (melanoma, melanocytoma, melanosarcoma), mesenchymal skin tumors, nephroblastoma, neuroepithelial tumors, pancreatic carcinoma, squamous cell carcinomas, testicular tumors, tumors of the biliary system	YES
**Reptiles**	Organ/System organ	amniotic egg	closed circulatory system, double circulation, 3-chambered heart	innate and adaptive immunity	Cases with oligometastases and wide-spread metastases have been reported in sea turtles, crocodiles and snakes. Cancer types include squamous cell carcinomas, splenopancreatic ductal adenocarcinoma, renal cell carcinoma and ductal adenocarcinoma	YES
**Birds**	Organ/System organ	endothermy, homeothermy	closed circulatory system, double circulation, 4-chambered heart	innate and adaptive immunity	Cases with oligometastases and wide-spread metastases have been reported in macaw parrots, cockatiels, chickens and penguins. Cancer types include cholangiocarcinoma, cloacolithiasis and intestinal lymphosarcoma, oropharyngeals and cloacal papilloma, ventriculus carcinoma, bilateral seminoma, osteoblastic osteocarcinoma	YES
**Mammals**	Organ/System organ	hair, mammary glands	closed circulatory system, double circulation, 4-chambered heart	innate and adaptive immunity	Common cancer types across eutherian mammals include soft-tissue sarcoma, lymphoma and leukaemia, urothelial carcinoma, mammary tumour, genital neoplasms, uterine leiomyomas and squamous cell carcinoma. Metastatic cancers are often reported in mammalian species. Over 30 cancer types have been reported in humans	YES

Supplementary Table 2: Mouse gene knockouts which present jaws abnormalities. The human orthologues are also included. The ratio of the jaw-related phenotypes to all developmental phenotypes affected by each gene knockout is also shown.

MOUSE GENE SYMBOL	GENE NAME	Jaw-related phenotypes	number affected phenotypes			HUMAN GENE SYMBOL	HUMAN GENE NAME	HGNC_ID
			Jaw-related	all	ratio: Jaw-to-all			
Eps8l3	EPS8-like 3	abnormal maxilla morphology	1	2	0.500	EPS8L3	EPS8 like 3	HGNC:21297
Tgds	TDP-glucose 4,6-dehydratase	micrognathia	1	2	0.500	TGDS	TDP-glucose 4,6-dehydratase	HGNC:20324
Rbbp7	retinoblastoma binding protein 7, chromatin remodeling factor	abnormal maxilla morphology	1	4	0.250	RBBP7	RB binding protein 7, chromatin remodeling factor	HGNC:9890
Dlx6	distal-less homeobox 6	abnormal jaw morphology; abnormal maxilla morphology; abnormal premaxilla morphology; absent Meckel's cartilage; small mandible; abnormal mandibular coronoid process morphology; abnormal mandibular condyloid process morphology; abnormal mandibular angle morphology; absent mandibular coronoid process; abnormal mandibular prominence morphology; mandibular condyloid process hypoplasia; lower jaw to upper jaw transformation	12	53	0.226	DLX6	distal-less homeobox 6	HGNC:2919
Atpaf2	ATP synthase mitochondrial F1 complex assembly factor 2	abnormal maxilla morphology	1	6	0.167	ATPAF2	ATP synthase mitochondrial F1 complex assembly factor 2	HGNC:18802
Rspo2	R-spondin 2	short mandible; abnormal maxilla morphology; abnormal maxillary shelf morphology; small maxilla; small mandible; abnormal mandibular angle morphology; absent mandibular coronoid process; maxillary shelf hypoplasia; short Meckel's cartilage; small mandibular condyloid process; abnormal maxillary zygomatic process morphology	11	66	0.167	RSPO2	R-spondin 2	HGNC:28583
Mau2	MAU2 sister chromatid cohesion factor	short mandible; abnormal jaw morphology; small mandible	3	19	0.158	MAU2	MAU2 sister chromatid cohesion factor	HGNC:29140
Satb2	special AT-rich sequence binding protein 2	short mandible; short maxilla; abnormal jaw morphology; abnormal maxilla morphology; abnormal mandible morphology; micrognathia; abnormal premaxilla morphology; premaxilla hypoplasia	8	53	0.151	SATB2	SATB homeobox 2	HGNC:21637
Arhgap29	Rho GTPase activating protein 29	palatal shelf fusion with tongue or mandible	1	7	0.143	ARHGAP29	Rho GTPase activating protein 29	HGNC:30207
Rfng	RFNG O-fucosylpeptide 3-beta-N-acetylglucosaminyltransferase	short mandible	1	7	0.143	RFNG	RFNG O-fucosylpeptide 3-beta-N-acetylglucosaminyltransferase	HGNC:9974
Schip1	schwannomin interacting protein 1	short premaxilla; short maxilla	2	14	0.143	SCHIP1	schwannomin interacting protein 1	HGNC:15678
Wdr19	WD repeat domain 19	short mandible; short premaxilla; short maxilla; abnormal maxilla morphology; abnormal premaxilla morphology; abnormal mandibular angle morphology	6	43	0.140	WDR19	WD repeat domain 19	HGNC:18340

Mapk1	mitogen-activated protein kinase 1	short maxilla; maxilla hypoplasia; abnormal mandible morphology; mandible hypoplasia; micrognathia; small mandible; abnormal mandibular coronoid process morphology; abnormal mandibular angle morphology; abnormal Meckel's cartilage morphology; small Meckel's cartilage; small mandibular condyloid process	11	82	0.134	MAPK1	mitogen-activated protein kinase 1	HGNC:6871
Cdon	cell adhesion molecule-related/down-regulated by oncogenes	absent maxillary shelf; abnormal maxilla morphology; abnormal premaxilla morphology; abnormal maxillary shelf morphology; decreased maxillary shelf size	5	38	0.132	CDON	cell adhesion associated, oncogene regulated	HGNC:17104
Foxi3	forkhead box I3	abnormal maxilla morphology; abnormal mandible morphology; abnormal Meckel's cartilage morphology; syngnathia	4	31	0.129	FOXI3	forkhead box I3	HGNC:35123
Hhat	hedgehog acyltransferase	abnormal mandible morphology; absent mandibular condyloid process; absent mandibular coronoid process; absent mandibular angle; abnormal mandibular prominence morphology; abnormal maxillary prominence morphology; agnathia	7	55	0.127	HHAT	hedgehog acyltransferase	HGNC:18270
Mthfd1l	methylenetetrahydrofolate dehydrogenase (NADP+ dependent) 1-like	abnormal mandibular prominence morphology; abnormal maxillary prominence morphology	2	16	0.125	MTHFD1L	methylenetetrahydrofolate dehydrogenase (NADP+ dependent) 1 like	HGNC:21055
Zfp366	zinc finger protein 366	micrognathia	1	8	0.125	ZNF366	zinc finger protein 366	HGNC:18316
Tcof1	treacle ribosome biogenesis factor 1	short mandible; short premaxilla; short maxilla; abnormal maxilla morphology; maxilla hypoplasia; mandible hypoplasia; abnormal premaxilla morphology; retrognathia; small mandible; premaxilla hypoplasia; abnormal maxillary prominence morphology	11	96	0.115	TCOF1	treacle ribosome biogenesis factor 1	HGNC:11654
Gsc	goosecoid homeobox	short mandible; abnormal jaw morphology; abnormal maxilla morphology; abnormal mandible morphology; mandible hypoplasia; abnormal mandibular angle morphology; abnormal Meckel's cartilage morphology; small mandibular coronoid process	8	70	0.114	GSC	goosecoid homeobox	HGNC:4612
Ambn	ameloblastin	abnormal jaw morphology	1	9	0.111	AMBN	ameloblastin	HGNC:452
Cask	calcium/calmodulin-dependent serine protein kinase (MAGUK family)	short mandible; abnormal maxilla morphology; mandibular retrognathia	3	27	0.111	CASK	calcium/calmodulin dependent serine protein kinase	HGNC:1497
Kcnj2	potassium inwardly-rectifying channel, subfamily J, member 2	narrow maxilla	1	9	0.111	KCNJ2	potassium inwardly rectifying channel subfamily J member 2	HGNC:6263
Dlx5	distal-less homeobox 5	short mandible; short premaxilla; short maxilla; abnormal jaw morphology; abnormal maxilla morphology; abnormal mandible morphology; micrognathia; abnormal premaxilla morphology; absent Meckel's cartilage; small maxilla; abnormal mandibular coronoid process morphology; abnormal mandibular condyloid process morphology; abnormal mandibular angle morphology; absent mandibular coronoid process; abnormal Meckel's cartilage morphology; decreased maxillary shelf size; abnormal mandibular prominence morphology; short Meckel's cartilage; lower jaw to upper jaw transformation	19	172	0.110	DLX5	distal-less homeobox 5	HGNC:2918
Zic5	zinc finger protein of the cerebellum 5	absent mandible; micrognathia; small mandible; small premaxilla	4	37	0.108	ZIC5	Zic family member 5	HGNC:20322

Slc39a13	solute carrier family 39 (metal ion transporter), member 13	abnormal jaw morphology; small maxilla; small mandible	3	28	0.107	SLC39A13	solute carrier family 39 member 13	HGNC:20859
Prdm16	PR domain containing 16	short maxilla; abnormal mandible morphology; mandible hypoplasia; micrognathia; small mandible; short Meckel's cartilage; narrow maxilla	7	69	0.101	PRDM16	PR/SET domain 16	HGNC:14000
Daam2	dishevelled associated activator of morphogenesis 2	abnormal maxilla morphology	1	10	0.100	DAAM2	dishevelled associated activator of morphogenesis 2	HGNC:18143
Pappa2	pappalysin 2	short mandible; abnormal mandible morphology	2	20	0.100	PAPPA2	pappalysin 2	HGNC:14615
Ryk	receptor-like tyrosine kinase	short mandible; abnormal maxilla morphology	2	20	0.100	RYK	receptor like tyrosine kinase	HGNC:10481
Msx1	msh homeobox 1	short mandible; absent premaxilla; short maxilla; absent maxillary shelf; abnormal mandible morphology	5	52	0.096	MSX1	msh homeobox 1	HGNC:7391
Hat1	histone aminotransferase 1	absent mandible; abnormal mandible morphology	2	21	0.095	HAT1	histone acetyltransferase 1	HGNC:4821
Foxd3	forkhead box D3	short mandible; short premaxilla; short maxilla; mandibular hyperostosis	4	43	0.093	FOXD3	forkhead box D3	HGNC:3804
Ednra	endothelin receptor type A	short mandible; abnormal jaw morphology; abnormal maxilla morphology; abnormal mandible morphology; mandible hypoplasia; micrognathia; absent Meckel's cartilage; abnormal mandibular condyloid process morphology; abnormal Meckel's cartilage morphology; abnormal temporomandibular joint morphology; lower jaw to upper jaw transformation	11	119	0.092	EDNRA	endothelin receptor type A	HGNC:3179
Amelx	amelogenin, X-linked	abnormal mandible morphology; large mandible	2	22	0.091	AMELX	amelogenin X-linked	HGNC:461
Hapln1	hyaluronan and proteoglycan link protein 1	small mandible; abnormal Meckel's cartilage morphology	2	22	0.091	HAPLN1	hyaluronan and proteoglycan link protein 1	HGNC:2380
Pax9	paired box 9	absent premaxilla; absent maxillary shelf; abnormal mandible morphology; abnormal maxillary shelf morphology; absent mandibular coronoid process	5	55	0.091	PAX9	paired box 9	HGNC:8623
Wwp2	WW domain containing E3 ubiquitin protein ligase 2	abnormal jaw morphology	1	11	0.091	WWP2	WW domain containing E3 ubiquitin protein ligase 2	HGNC:16804
Prrx1	paired related homeobox 1	absent maxillary shelf; abnormal mandible morphology; mandible hypoplasia; abnormal Meckel's cartilage morphology; maxillary shelf hypoplasia; absent temporomandibular joint	6	68	0.088	PRRX1	paired related homeobox 1	HGNC:9142
Brd4	bromodomain containing 4	short mandible; short premaxilla	2	23	0.087	BRD4	bromodomain containing 4	HGNC:13575
Dph1	diphthamide biosynthesis 1	short mandible; mandible hypoplasia; micrognathia; short Meckel's cartilage	4	46	0.087	DPH1	diphthamide biosynthesis 1	HGNC:3003
Flvcr1	feline leukemia virus subgroup C cellular receptor 1	absent mandible; small Meckel's cartilage	2	23	0.087	FLVCR1	FLVCR heme transporter 1	HGNC:24682
Ifitm5	interferon induced transmembrane protein 5	short mandible; abnormal mandible morphology; mandible hypoplasia; abnormal premaxilla morphology	4	47	0.085	IFITM5	interferon induced transmembrane protein 5	HGNC:16644
Grem2	gremlin 2, DAN family BMP antagonist	small mandible	1	12	0.083	GREM2	gremlin 2, DAN family BMP antagonist	HGNC:17655
Hand2	heart and neural crest derivatives expressed 2	short mandible; absent maxillary shelf; abnormal jaw morphology; abnormal mandible morphology; mandible hypoplasia; small mandible; abnormal mandibular angle morphology; abnormal Meckel's cartilage morphology; short Meckel's cartilage	9	113	0.080	HAND2	heart and neural crest derivatives expressed 2	HGNC:4808

Fuz	fuzzy planar cell polarity protein	maxilla hypoplasia; abnormal mandible morphology; mandible hypoplasia; abnormal Meckel's cartilage morphology; Meckel's cartilage hyperplasia	5	63	0.079	FUZ	fuzzy planar cell polarity protein	HGNC:26219
Acvr1	activin A receptor, type 1	short mandible; abnormal mandible morphology; mandible hypoplasia; abnormal Meckel's cartilage morphology; absent temporomandibular joint; absent mandibular symphysis; small mandibular coronoid process; abnormal mandibular fossa morphology	8	104	0.077	ACVR1	activin A receptor type 1	HGNC:171
Alx1	ALX homeobox 1	short mandible; short maxilla	2	26	0.077	ALX1	ALX homeobox 1	HGNC:1494
Gkn1	gastrokine 1	abnormal mandible morphology	1	13	0.077	GKN1	gastrokine 1	HGNC:23217
Hand1	heart and neural crest derivatives expressed 1	short premaxilla; abnormal maxilla morphology; mandible hypoplasia; small mandible; premaxilla hypoplasia; decreased maxillary shelf size; abnormal maxillary prominence morphology; short Meckel's cartilage	8	104	0.077	HAND1	heart and neural crest derivatives expressed 1	HGNC:4807
Mef2c	myocyte enhancer factor 2C	short mandible; mandible hypoplasia; abnormal mandibular angle morphology; mandibular condyloid process hypoplasia; mandibular coronoid process hypoplasia; Meckel's cartilage hypoplasia	6	78	0.077	MEF2C	myocyte enhancer factor 2C	HGNC:6996
Pigv	phosphatidylinositol glycan anchor biosynthesis, class V	micrognathia	1	13	0.077	PIGV	phosphatidylinositol glycan anchor biosynthesis class V	HGNC:26031
Pitx1	paired-like homeodomain transcription factor 1	short mandible; micrognathia; small mandible; abnormal Meckel's cartilage morphology	4	52	0.077	PITX1	paired like homeodomain 1	HGNC:9004
Qsox1	quiescin Q6 sulfhydryl oxidase 1	micrognathia	1	13	0.077	QSOX1	quiescin sulfhydryl oxidase 1	HGNC:9756
Ski	ski sarcoma viral oncogene homolog (avian)	short mandible; abnormal jaw morphology; mandibular hyperostosis	3	39	0.077	SKI	SKI proto-oncogene	HGNC:10896
Slc26a2	solute carrier family 26 (sulfate transporter), member 2	mandibular hyperostosis	1	13	0.077	SLC26A2	solute carrier family 26 member 2	HGNC:10994
Nipbl	NIPBL cohesin loading factor	short mandible; abnormal jaw morphology; maxilla hypoplasia; mandible hypoplasia; small mandible	5	66	0.076	NIPBL	NIPBL cohesin loading factor	HGNC:28862
Chrd	chordin	absent mandible; short mandible; abnormal mandible morphology; small mandible; absent mandibular condyloid process; absent mandibular coronoid process; absent mandibular angle	7	94	0.074	CHRD	chordin	HGNC:1949
Ybx1	Y box protein 1	abnormal maxilla morphology; abnormal mandible morphology	2	27	0.074	YBX1	Y-box binding protein 1	HGNC:8014
Ift27	intraflagellar transport 27	abnormal jaw morphology; micrognathia; small mandible	3	41	0.073	IFT27	intraflagellar transport 27	HGNC:18626
Dvl3	dishevelled segment polarity protein 3	micrognathia	1	14	0.071	DVL3	dishevelled segment polarity protein 3	HGNC:3087
Mmp21	matrix metallopeptidase 21	micrognathia	1	14	0.071	MMP21	matrix metallopeptidase 21	HGNC:14357
Otulin	OTU deubiquitinase with linear linkage specificity	micrognathia	1	14	0.071	OTULIN	OTU deubiquitinase with linear linkage specificity	HGNC:25118
Asap1	ArfGAP with SH3 domain, ankyrin repeat and PH domain1	abnormal mandible morphology; abnormal premaxilla morphology	2	30	0.067	ASAP1	ArfGAP with SH3 domain, ankyrin repeat and PH domain 1	HGNC:2720
Loxl3	lysyl oxidase-like 3	short mandible	1	15	0.067	LOXL3	lysyl oxidase like 3	HGNC:13869
Nfic	nuclear factor I/C	small mandible	1	15	0.067	NFIC	nuclear factor I C	HGNC:7786
Spry4	sprouty RTK signaling antagonist 4	abnormal mandible morphology	1	15	0.067	SPRY4	sprouty RTK signaling antagonist 4	HGNC:15533

Pgap1	post-GPI attachment to proteins 1	absent mandible; micrognathia; absent maxilla; small mandible	4	62	0.065	PGAP1	post-GPI attachment to proteins inositol deacylase 1	HGNC:25712
Prkra	protein kinase, interferon inducible double stranded RNA dependent activator	small mandibular condyloid process; small mandibular coronoid process	2	31	0.065	PRKRA	protein activator of interferon induced protein kinase EIF2AK2	HGNC:9438
Ror2	receptor tyrosine kinase-like orphan receptor 2	short mandible; abnormal jaw morphology; abnormal maxilla morphology; abnormal mandible morphology; abnormal mandibular condyloid process morphology; abnormal mandibular angle morphology; abnormal Meckel's cartilage morphology; short Meckel's cartilage	8	127	0.063	ROR2	receptor tyrosine kinase like orphan receptor 2	HGNC:10257
Ap2b1	adaptor-related protein complex 2, beta 1 subunit	micrognathia	1	16	0.063	AP2B1	adaptor related protein complex 2 subunit beta 1	HGNC:563
Kcnj13	potassium inwardly-rectifying channel, subfamily J, member 13	absent maxillary shelf	1	16	0.063	KCNJ13	potassium inwardly rectifying channel subfamily J member 13	HGNC:6259
Zeb1	zinc finger E-box binding homeobox 1	short mandible; short premaxilla; short maxilla; abnormal jaw morphology; mandible hypoplasia; abnormal premaxilla morphology; abnormal maxillary shelf morphology; abnormal mandibular angle morphology; mandibular condyloid process hypoplasia; mandibular coronoid process hypoplasia; Meckel's cartilage hyperplasia	11	179	0.061	ZEB1	zinc finger E-box binding homeobox 1	HGNC:11642
Ltbp1	latent transforming growth factor beta binding protein 1	short mandible; short maxilla; abnormal jaw morphology	3	49	0.061	LTBP1	latent transforming growth factor beta binding protein 1	HGNC:6714
Ccnd1	cyclin D1	abnormal maxilla morphology; abnormal mandible morphology	2	33	0.061	CCND1	cyclin D1	HGNC:1582
Cacna1s	calcium channel, voltage-dependent, L type, alpha 1S subunit	short mandible; abnormal mandible morphology; micrognathia; small mandibular condyloid process; small mandibular coronoid process	5	83	0.060	CACNA1S	calcium voltage-gated channel subunit alpha1 S	HGNC:1397
Pkdcc	protein kinase domain containing, cytoplasmic	short maxilla; abnormal maxilla morphology; small maxilla	3	50	0.060	PKDCC	protein kinase domain containing, cytoplasmic	HGNC:25123
Kif3a	kinesin family member 3A	short mandible; abnormal maxilla morphology; abnormal premaxilla morphology; absent mandibular condyloid process; absent mandibular ramus	5	84	0.060	KIF3A	kinesin family member 3A	HGNC:6319
Sox11	SRY (sex determining region Y)-box 11	short mandible; maxilla hypoplasia; abnormal mandible morphology; mandible hypoplasia; small Meckel's cartilage	5	84	0.060	SOX11	SRY-box transcription factor 11	HGNC:11191
Baz1b	bromodomain adjacent to zinc finger domain, 1B	short mandible; mandible hypoplasia	2	34	0.059	BAZ1B	bromodomain adjacent to zinc finger domain 1B	HGNC:961
Bnc2	basonuclin 2	abnormal maxillary shelf morphology	1	17	0.059	BNC2	basonuclin 2	HGNC:30988
Gli2	GLI-Kruppel family member GLI2	short maxilla; absent maxillary shelf; small mandible; small mandibular condyloid process; abnormal maxillary frontal process morphology; absent temporomandibular joint disk	6	103	0.058	GLI2	GLI family zinc finger 2	HGNC:4318
Tgfb2	transforming growth factor, beta 2	short mandible; absent maxillary shelf; abnormal mandible morphology; retrognathia; absent mandibular angle; small mandibular condyloid process; small mandibular coronoid process	7	122	0.057	TGFB2	transforming growth factor beta 2	HGNC:11768
Col11a1	collagen, type XI, alpha 1	short mandible; small Meckel's cartilage	2	35	0.057	COL11A1	collagen type XI alpha 1 chain	HGNC:2186
Dlx2	distal-less homeobox 2	absent maxillary shelf; abnormal maxilla morphology	2	35	0.057	DLX2	distal-less homeobox 2	HGNC:2915
Sc5d	sterol-C5-desaturase	mandible hypoplasia; micrognathia	2	35	0.057	SC5D	sterol-C5-desaturase	HGNC:10547

Tfap2a	transcription factor AP-2, alpha	short mandible; short maxilla; abnormal maxilla morphology; abnormal mandible morphology; abnormal premaxilla morphology; absent maxilla; abnormal maxillary prominence morphology	7	125	0.056	TFAP2A	transcription factor AP-2 alpha	HGNC:11742
Lpar4	lysophosphatidic acid receptor 4	absent mandible	1	18	0.056	LPAR4	lysophosphatidic acid receptor 4	HGNC:4478
Mfsd12	major facilitator superfamily domain containing 12	abnormal jaw morphology	1	18	0.056	MFSD12	major facilitator superfamily domain containing 12	HGNC:28299
Nabp2	nucleic acid binding protein 2	abnormal mandible morphology; mandible hypoplasia; micrognathia; abnormal premaxilla morphology	4	73	0.055	NABP2	nucleic acid binding protein 2	HGNC:28412
Trps1	transcriptional repressor GATA binding 1	short mandible; short maxilla; micrognathia	3	55	0.055	TRPS1	transcriptional repressor GATA binding 1	HGNC:12340
Cplane2	ciliogenesis and planar polarity effector 2	micrognathia	1	19	0.053	CPLANE2	ciliogenesis and planar polarity effector 2	HGNC:28127
Dctn5	dynactin 5	micrognathia	1	19	0.053	DCTN5	dynactin subunit 5	HGNC:24594
Drc1	dynein regulatory complex subunit 1	micrognathia	1	19	0.053	DRC1	dynein regulatory complex subunit 1	HGNC:24245
Ift57	intraflagellar transport 57	abnormal maxilla morphology	1	19	0.053	IFT57	intraflagellar transport 57	HGNC:17367
Irf6	interferon regulatory factor 6	abnormal jaw morphology; small mandible; abnormal mandibular angle morphology; palatal shelf fusion with tongue or mandible	4	76	0.053	IRF6	interferon regulatory factor 6	HGNC:6121
Mmp2	matrix metallopeptidase 2	short mandible; short maxilla	2	39	0.051	MMP2	matrix metallopeptidase 2	HGNC:7166
Cnbp	cellular nucleic acid binding protein	small mandible	1	20	0.050	CNBP	CCHC-type zinc finger nucleic acid binding protein	HGNC:13164
Crkl	v-crk avian sarcoma virus CT10 oncogene homolog-like	absent mandible; mandible hypoplasia	2	40	0.050	CRKL	CRK like proto-oncogene, adaptor protein	HGNC:2363
Kdf1	keratinocyte differentiation factor 1	palatal shelf fusion with tongue or mandible	1	20	0.050	KDF1	keratinocyte differentiation factor 1	HGNC:26624
Ostm1	osteopetrosis associated transmembrane protein 1	abnormal maxilla morphology; abnormal maxillary zygomatic process morphology	2	40	0.050	OSTM1	osteoclastogenesis associated transmembrane protein 1	HGNC:21652
Pak1ip1	PAK1 interacting protein 1	abnormal maxilla morphology	1	20	0.050	PAK1IP1	PAK1 interacting protein 1	HGNC:20882
Trappc10	trafficking protein particle complex 10	micrognathia	1	20	0.050	TRAPPC10	trafficking protein particle complex 10	HGNC:11868
Bmp4	bone morphogenetic protein 4	abnormal jaw morphology; maxilla hypoplasia; abnormal mandible morphology; mandible hypoplasia; retrognathia; absent mandibular condyloid process; absent mandibular coronoid process; absent mandibular angle; palatal shelf fusion with tongue or mandible; abnormal temporomandibular joint morphology; syngnathia; agnathia	12	245	0.049	BMP4	bone morphogenetic protein 4	HGNC:1071
Chst11	carbohydrate sulfotransferase 11	short mandible; short maxilla	2	41	0.049	CHST11	carbohydrate sulfotransferase 11	HGNC:17422
Jag2	jagged 2	absent maxillary shelf; palatal shelf fusion with tongue or mandible	2	41	0.049	JAG2	jagged canonical Notch ligand 2	HGNC:6189
Bmp7	bone morphogenetic protein 7	abnormal maxilla morphology; abnormal mandible morphology; abnormal maxillary shelf morphology; small Meckel's cartilage	4	83	0.048	BMP7	bone morphogenetic protein 7	HGNC:1074
Cdk10	cyclin-dependent kinase 10	mandible hypoplasia	1	21	0.048	CDK10	cyclin dependent kinase 10	HGNC:1770

Mbtps1	membrane-bound transcription factor peptidase, site 1	short mandible; short maxilla	2	42	0.048	MBTPS1	membrane bound transcription factor peptidase, site 1	HGNC:15456
Miga2	mitoguardin 2	abnormal maxilla morphology	1	21	0.048	MIGA2	mitoguardin 2	HGNC:23621
Slc25a21	solute carrier family 25 (mitochondrial oxodicarboxylate carrier), member 21	abnormal mandible morphology	1	21	0.048	SLC25A21	solute carrier family 25 member 21	HGNC:14411
Vps25	vacuolar protein sorting 25	mandible hypoplasia	1	21	0.048	VPS25	vacuolar protein sorting 25 homolog	HGNC:28122
Dkk1	dickkopf WNT signaling pathway inhibitor 1	absent mandible; absent maxilla; abnormal mandibular prominence morphology	3	64	0.047	DKK1	dickkopf WNT signaling pathway inhibitor 1	HGNC:2891
Ide	insulin degrading enzyme	abnormal maxilla morphology	1	22	0.045	IDE	insulin degrading enzyme	HGNC:5381
Mnt	max binding protein	small mandible	1	22	0.045	MNT	MAX network transcriptional repressor	HGNC:7188
Ttc28	tetratricopeptide repeat domain 28	abnormal maxilla morphology	1	22	0.045	TTC28	tetratricopeptide repeat domain 28	HGNC:29179
Otx2	orthodenticle homeobox 2	absent mandible; abnormal mandible morphology; micrognathia; absent maxilla; small mandible; absent mandibular coronoid process; abnormal Meckel's cartilage morphology; abnormal maxillary prominence morphology	8	178	0.045	OTX2	orthodenticle homeobox 2	HGNC:8522
Ift140	intraflagellar transport 140	abnormal maxilla morphology; maxilla hypoplasia; premaxilla hypoplasia	3	67	0.045	IFT140	intraflagellar transport 140	HGNC:29077
Zic3	zinc finger protein of the cerebellum 3	abnormal jaw morphology; maxilla hypoplasia; abnormal mandible morphology; premaxilla hypoplasia; mandibular coronoid process hypoplasia	5	112	0.045	ZIC3	Zic family member 3	HGNC:12874
Fign	fidgetin	abnormal mandible morphology; absent mandibular foramen	2	45	0.044	FIGN	fidgetin, microtubule severing factor	HGNC:13285
Smad2	SMAD family member 2	absent mandible; abnormal mandible morphology; mandible hypoplasia; abnormal Meckel's cartilage morphology	4	90	0.044	SMAD2	SMAD family member 2	HGNC:6768
Cplane1	ciliogenesis and planar polarity effector 1	mandible hypoplasia; micrognathia	2	46	0.043	CPLANE1	ciliogenesis and planar polarity effector 1	HGNC:25801
Ctnnbip1	catenin beta interacting protein 1	absent maxilla	1	23	0.043	CTNNBIP 1	catenin beta interacting protein 1	HGNC:16913
Nisch	nischarin	abnormal maxilla morphology	1	23	0.043	NISCH	nischarin	HGNC:18006
Fgfr2	fibroblast growth factor receptor 2	short mandible; short premaxilla; short maxilla; abnormal mandible morphology; small mandible; abnormal mandibular angle morphology; palatal shelf fusion with tongue or mandible; abnormal maxillary-premaxillary suture morphology; abnormal intermaxillary suture morphology; abnormal nasomaxillary suture morphology; abnormal maxillary frontal process morphology; abnormal maxillary zygomatic process morphology; premature zygomaticomaxillary suture closure; premature palatomaxillary suture closure; wide intermaxillary suture; premature maxillary-premaxillary suture closure	16	377	0.042	FGFR2	fibroblast growth factor receptor 2	HGNC:3689
Hoxa3	homeobox A3	short mandible; short maxilla	2	48	0.042	HOXA3	homeobox A3	HGNC:5104
Hspb11	heat shock protein family B (small), member 11	micrognathia	1	24	0.042	HSPB11	heat shock protein family B (small) member 11	HGNC:25019

Pfas	phosphoribosylformylglycinamidine synthase (FGAR amidotransferase)	short maxilla	1	24	0.042	PFAS	phosphoribosylformylglycinamidine synthase	HGNC:8863
Prickle1	prickle planar cell polarity protein 1	short mandible; short premaxilla; micrognathia	3	73	0.041	PRICKLE1	prickle planar cell polarity protein 1	HGNC:17019
Ece1	endothelin converting enzyme 1	absent Meckel's cartilage; small mandible	2	49	0.041	ECE1	endothelin converting enzyme 1	HGNC:3146
Edn1	endothelin 1	mandible hypoplasia; abnormal Meckel's cartilage morphology	2	49	0.041	EDN1	endothelin 1	HGNC:3176
Eya1	EYA transcriptional coactivator and phosphatase 1	absent premaxilla; abnormal jaw morphology; mandible hypoplasia; absent maxilla; decreased maxillary shelf size	5	124	0.040	EYA1	EYA transcriptional coactivator and phosphatase 1	HGNC:3519
Limk2	LIM motif-containing protein kinase 2	short mandible	1	25	0.040	LIMK2	LIM domain kinase 2	HGNC:6614
Ltbp3	latent transforming growth factor beta binding protein 3	short maxilla; prognathia	2	50	0.040	LTBP3	latent transforming growth factor beta binding protein 3	HGNC:6716
Pou3f3	POU domain, class 3, transcription factor 3	absent temporomandibular joint	1	25	0.040	POU3F3	POU class 3 homeobox 3	HGNC:9216
Tiparp	TCDD-inducible poly(ADP-ribose) polymerase	short premaxilla; short maxilla	2	50	0.040	TIPARP	TCDD inducible poly(ADP-ribose) polymerase	HGNC:23696
Col27a1	collagen, type XXVII, alpha 1	mandibular retrognathia	1	26	0.038	COL27A1	collagen type XXVII alpha 1 chain	HGNC:22986
Foxn3	forkhead box N3	mandible hypoplasia	1	26	0.038	FOXN3	forkhead box N3	HGNC:1928
Xylt1	xylosyltransferase 1	short mandible	1	26	0.038	XYLT1	xylosyltransferase 1	HGNC:15516
Pcsk6	proprotein convertase subtilisin/kexin type 6	agnathia	1	27	0.037	PCSK6	proprotein convertase subtilisin/kexin type 6	HGNC:8569
Tgfbr2	transforming growth factor, beta receptor II	abnormal mandible morphology; small maxilla; small mandible; absent mandibular angle; small mandibular condyloid process; small mandibular coronoid process	6	163	0.037	TGFBR2	transforming growth factor beta receptor 2	HGNC:11773
Sh3pxd2b	SH3 and PX domains 2B	short mandible; short maxilla; micrognathia	3	82	0.037	SH3PXD2B	SH3 and PX domains 2B	HGNC:29242
Chd1	chromodomain helicase DNA binding protein 1	abnormal maxilla morphology	1	28	0.036	CHD1	chromodomain helicase DNA binding protein 1	HGNC:1915
Dhrs3	dehydrogenase/reductase (SDR family) member 3	micrognathia	1	28	0.036	DHRS3	dehydrogenase/reductase 3	HGNC:17693
Lrrk1	leucine-rich repeat kinase 1	abnormal maxilla morphology; abnormal mandible morphology	2	56	0.036	LRRK1	leucine rich repeat kinase 1	HGNC:18608
Ctsk	cathepsin K	abnormal jaw morphology; absent mandibular angle	2	57	0.035	CTSK	cathepsin K	HGNC:2536
Gldc	glycine decarboxylase	absent mandible	1	29	0.034	GLDC	glycine decarboxylase	HGNC:4313
Glg1	golgi apparatus protein 1	abnormal maxillary shelf morphology	1	29	0.034	GLG1	golgi glycoprotein 1	HGNC:4316
Kat6b	K(lysine) acetyltransferase 6B	short mandible	1	29	0.034	KAT6B	lysine acetyltransferase 6B	HGNC:17582
Sox9	SRY (sex determining region Y)-box 9	short mandible; short maxilla; micrognathia; absent Meckel's cartilage; small mandible; abnormal Meckel's cartilage morphology	6	174	0.034	SOX9	SRY-box transcription factor 9	HGNC:11204
Mmp14	matrix metallopeptidase 14 (membrane-inserted)	short mandible; short maxilla; small mandible; abnormal Meckel's cartilage morphology	4	118	0.034	MMP14	matrix metallopeptidase 14	HGNC:7160
Dnaic1	dynein, axonemal, intermediate chain 1	micrognathia	1	30	0.033	DNAI1	dynein axonemal intermediate chain 1	HGNC:2954

Dock1	dedicator of cytokinesis 1	micrognathia	1	30	0.033	DOCK1	dedicator of cytokinesis 1	HGNC:2987
Fam20c	family with sequence similarity 20, member C	abnormal jaw morphology; abnormal maxilla morphology; mandibular hyperostosis	3	90	0.033	FAM20C	FAM20C golgi associated secretory pathway kinase	HGNC:22140
Fbln1	fibulin 1	micrognathia; abnormal premaxilla morphology	2	60	0.033	FBLN1	fibulin 1	HGNC:3600
Tbx15	T-box 15	short mandible; abnormal mandibular angle morphology	2	60	0.033	TBX15	T-box transcription factor 15	HGNC:11594
Six1	sine oculis-related homeobox 1	short mandible; short maxilla; micrognathia; abnormal Meckel's cartilage morphology	4	121	0.033	SIX1	SIX homeobox 1	HGNC:10887
Sfn	stratifin	abnormal jaw morphology; abnormal mandible morphology; palatal shelf fusion with tongue or mandible	3	91	0.033	SFN	stratifin	HGNC:10773
Dync2h1	dynein cytoplasmic 2 heavy chain 1	micrognathia	1	31	0.032	DYNC2H1	dynein cytoplasmic 2 heavy chain 1	HGNC:2962
Sostdc1	sclerostin domain containing 1	short mandible	1	31	0.032	SOSTDC1	sclerostin domain containing 1	HGNC:21748
Foxc2	forkhead box C2	small mandible; abnormal Meckel's cartilage morphology; small Meckel's cartilage	3	95	0.032	FOXC2	forkhead box C2	HGNC:3801
Gpc3	glypican 3	absent mandible; mandible hypoplasia	2	64	0.031	GPC3	glypican 3	HGNC:4451
Hras	Harvey rat sarcoma virus oncogene	short premaxilla; short maxilla	2	64	0.031	HRAS	HRas proto-oncogene, GTPase	HGNC:5173
Mn1	meningioma 1	maxillary shelf hypoplasia	1	32	0.031	MN1	MN1 proto-oncogene, transcriptional regulator	HGNC:7180
Acvr2a	activin receptor IIA	mandible hypoplasia	1	33	0.030	ACVR2A	activin A receptor type 2A	HGNC:173
Cyb561	cytochrome b-561	abnormal maxilla morphology	1	33	0.030	CYB561	cytochrome b561	HGNC:2571
Pax7	paired box 7	short maxilla	1	33	0.030	PAX7	paired box 7	HGNC:8621
Smo	smoothened, frizzled class receptor	abnormal maxilla morphology; abnormal mandible morphology; abnormal mandibular condyloid process morphology; short Meckel's cartilage; Meckel's cartilage hypoplasia	5	167	0.030	SMO	smoothened, frizzled class receptor	HGNC:11119
Cyp51	cytochrome P450, family 51	micrognathia	1	34	0.029	CYP51A1	cytochrome P450 family 51 subfamily A member 1	HGNC:2649
Foxf2	forkhead box F2	abnormal maxillary shelf morphology	1	34	0.029	FOXF2	forkhead box F2	HGNC:3810
Gad1	glutamate decarboxylase 1	narrow maxilla	1	34	0.029	GAD1	glutamate decarboxylase 1	HGNC:4092
Pdss2	prenyl (solanesyl) diphosphate synthase, subunit 2	short mandible; micrognathia	2	68	0.029	PDSS2	decaprenyl diphosphate synthase subunit 2	HGNC:23041
Spry2	sprouty RTK signaling antagonist 2	short mandible	1	34	0.029	SPRY2	sprouty RTK signaling antagonist 2	HGNC:11270
Kat14	lysine acetyltransferase 14	abnormal maxilla morphology	1	35	0.029	KAT14	lysine acetyltransferase 14	HGNC:15904
Shox2	short stature homeobox 2	abnormal temporomandibular joint disk morphology	1	35	0.029	SHOX2	short stature homeobox 2	HGNC:10854
Ccn2	cellular communication network factor 2	short mandible; abnormal Meckel's cartilage morphology	2	71	0.028	CCN2	cellular communication network factor 2	HGNC:2500
Pkd1	polycystin 1, transient receptor poteintial channel interacting	short mandible; short premaxilla; short maxilla; maxilla hypoplasia; micrognathia; abnormal Meckel's cartilage morphology	6	215	0.028	PKD1	polycystin 1, transient receptor potential channel interacting	HGNC:9008
Arid5b	AT rich interactive domain 5B (MRF1-like)	short premaxilla; short maxilla	2	73	0.027	ARID5B	AT-rich interaction domain 5B	HGNC:17362

Mks1	MKS transition zone complex subunit 1	mandible hypoplasia; micrognathia; absent maxilla	3	110	0.027	MKS1	MKS transition zone complex subunit 1	HGNC:7121
Cyp26b1	cytochrome P450, family 26, subfamily b, polypeptide 1	micrognathia	1	37	0.027	CYP26B1	cytochrome P450 family 26 subfamily B member 1	HGNC:20581
Ptch1	patched 1	abnormal mandibular prominence morphology; abnormal maxillary prominence morphology; short mandibular coronoid process; mandibular coronoid process hypoplasia	4	148	0.027	PTCH1	patched 1	HGNC:9585
Slc38a10	solute carrier family 38, member 10	abnormal maxilla morphology	1	37	0.027	SLC38A10	solute carrier family 38 member 10	HGNC:28237
Zmpste24	zinc metallopeptidase, STE24	abnormal mandible morphology; micrognathia	2	75	0.027	ZMPSTE24	zinc metallopeptidase STE24	HGNC:12877
Dlg1	discs large MAGUK scaffold protein 1	mandible hypoplasia; premaxilla hypoplasia	2	76	0.026	DLG1	discs large MAGUK scaffold protein 1	HGNC:2900
Klf2	Kruppel-like factor 2 (lung)	abnormal mandible morphology	1	38	0.026	KLF2	Kruppel like factor 2	HGNC:6347
Phex	phosphate regulating endopeptidase homolog, X-linked	short mandible; short premaxilla; short maxilla	3	117	0.026	PHEX	phosphate regulating endopeptidase homolog X-linked	HGNC:8918
Smg1	SMG1 homolog, phosphatidylinositol 3-kinase-related kinase (C. elegans)	prognathia	1	39	0.026	SMG1	SMG1 nonsense mediated mRNA decay associated PI3K related kinase	HGNC:30045
Ibsp	integrin binding sialoprotein	abnormal mandible morphology	1	40	0.025	IBSP	integrin binding sialoprotein	HGNC:5341
Col2a1	collagen, type II, alpha 1	short mandible; maxilla hypoplasia; retrognathia; premaxilla hypoplasia	4	161	0.025	COL2A1	collagen type II alpha 1 chain	HGNC:2200
Fgfr3	fibroblast growth factor receptor 3	abnormal maxilla morphology; abnormal premaxilla morphology; prognathia; maxillary retrognathia; abnormal maxillary zygomatic process morphology	5	202	0.025	FGFR3	fibroblast growth factor receptor 3	HGNC:3690
Robo1	roundabout guidance receptor 1	micrognathia	1	41	0.024	ROBO1	roundabout guidance receptor 1	HGNC:10249
Slc10a7	solute carrier family 10 (sodium/bile acid cotransporter family), member 7	abnormal mandibular angle morphology	1	41	0.024	SLC10A7	solute carrier family 10 member 7	HGNC:23088
Arsb	arylsulfatase B	short maxilla	1	42	0.024	ARSB	arylsulfatase B	HGNC:714
Psph	phosphoserine phosphatase	absent maxilla; fragmented Meckel's cartilage; short Meckel's cartilage	3	126	0.024	PSPH	phosphoserine phosphatase	HGNC:9577
Ptk7	PTK7 protein tyrosine kinase 7	micrognathia	1	42	0.024	PTK7	protein tyrosine kinase 7 (inactive)	HGNC:9618
Cdc73	cell division cycle 73, Paf1/RNA polymerase II complex component	abnormal jaw morphology	1	43	0.023	CDC73	cell division cycle 73	HGNC:16783
Grb2	growth factor receptor bound protein 2	abnormal mandible morphology	1	43	0.023	GRB2	growth factor receptor bound protein 2	HGNC:4566
Fbn2	fibrillin 2	micrognathia	1	44	0.023	FBN2	fibrillin 2	HGNC:3604
Fgf18	fibroblast growth factor 18	maxilla hypoplasia	1	44	0.023	FGF18	fibroblast growth factor 18	HGNC:3674

Aldh1a2	aldehyde dehydrogenase family 1, subfamily A2	abnormal mandibular prominence morphology; abnormal maxillary prominence morphology	2	89	0.022	ALDH1A2	aldehyde dehydrogenase 1 family member A2	HGNC:15472
Bmp5	bone morphogenetic protein 5	short mandible; short maxilla	2	89	0.022	BMP5	bone morphogenetic protein 5	HGNC:1072
Wnt5a	wingless-type MMTV integration site family, member 5A	short mandible; micrognathia	2	89	0.022	WNT5A	Wnt family member 5A	HGNC:12784
Rpgrip1l	Rpgrip1-like	abnormal maxilla morphology; abnormal mandible morphology; mandible hypoplasia	3	136	0.022	RPGRIP1L	RPGRIP1 like	HGNC:29168
Twsg1	twisted gastrulation BMP signaling modulator 1	abnormal jaw morphology; agnathia	2	92	0.022	TWSG1	twisted gastrulation BMP signaling modulator 1	HGNC:12429
Morc2a	microrchidia 2A	abnormal jaw morphology; abnormal Meckel's cartilage morphology; fragmented Meckel's cartilage	3	140	0.021	MORC2	MORC family CW-type zinc finger 2	HGNC:23573
Crim1	cysteine rich transmembrane BMP regulator 1 (chordin like)	abnormal Meckel's cartilage morphology; fragmented Meckel's cartilage	2	94	0.021	CRIM1	cysteine rich transmembrane BMP regulator 1	HGNC:2359
Slc27a4	solute carrier family 27 (fatty acid transporter), member 4	micrognathia	1	47	0.021	SLC27A4	solute carrier family 27 member 4	HGNC:10998
Tmem67	transmembrane protein 67	mandible hypoplasia	1	47	0.021	TMEM67	transmembrane protein 67	HGNC:28396
Crebbp	CREB binding protein	short premaxilla; short maxilla; maxilla hypoplasia	3	143	0.021	CREBBP	CREB binding protein	HGNC:2348
Fras1	Fraser extracellular matrix complex subunit 1	micrognathia	1	48	0.021	FRAS1	Fraser extracellular matrix complex subunit 1	HGNC:19185
Ccdc39	coiled-coil domain containing 39	micrognathia	1	49	0.020	CCDC39	coiled-coil domain containing 39	HGNC:25244
Gas2l2	growth arrest-specific 2 like 2	abnormal Meckel's cartilage morphology	1	49	0.020	GAS2L2	growth arrest specific 2 like 2	HGNC:24846
Kmt2d	lysine (K)-specific methyltransferase 2D	short maxilla	1	49	0.020	KMT2D	lysine methyltransferase 2D	HGNC:7133
Psip1	PC4 and SFRS1 interacting protein 1	abnormal jaw morphology	1	49	0.020	PSIP1	PC4 and SFRS1 interacting protein 1	HGNC:9527
Tent5a	terminal nucleotidyltransferase 5A	abnormal mandible morphology	1	49	0.020	TENT5A	terminal nucleotidyltransferase 5A	HGNC:18345
Arid1a	AT rich interactive domain 1A (SWI-like)	short premaxilla	1	50	0.020	ARID1A	AT-rich interaction domain 1A	HGNC:11110
Gas1	growth arrest specific 1	short maxilla	1	50	0.020	GAS1	growth arrest specific 1	HGNC:4165
Ctnnb1	catenin (cadherin associated protein), beta 1	absent mandible; abnormal maxillary shelf morphology; absent maxilla; abnormal Meckel's cartilage morphology; abnormal mandibular prominence morphology; abnormal maxillary prominence morphology; abnormal maxillary zygomatic process morphology	7	355	0.020	CTNNB1	catenin beta 1	HGNC:2514
Frem1	Fras1 related extracellular matrix protein 1	abnormal maxillary-premaxillary suture morphology	1	51	0.020	FREM1	FRAS1 related extracellular matrix 1	HGNC:23399
Gjb2	gap junction protein, beta 2	absent mandible	1	51	0.020	GJB2	gap junction protein beta 2	HGNC:4284
Npr2	natriuretic peptide receptor 2	short maxilla; abnormal mandibular condyloid process morphology	2	102	0.020	NPR2	natriuretic peptide receptor 2	HGNC:7944

Pds5b	PDS5 cohesin associated factor B	short mandible	1	51	0.020	PDS5B	PDS5 cohesin associated factor B	HGNC:20418
Pitx2	paired-like homeodomain transcription factor 2	abnormal maxilla morphology; abnormal mandible morphology; small Meckel's cartilage	3	153	0.020	PITX2	paired like homeodomain 2	HGNC:9005
Tnfrsf11b	tumor necrosis factor receptor superfamily, member 11b (osteoprotegerin)	abnormal mandible morphology	1	51	0.020	TNFRSF11B	TNF receptor superfamily member 11b	HGNC:11909
Postn	periostin, osteoblast specific factor	abnormal mandible morphology	1	52	0.019	POSTN	periostin	HGNC:16953
Foxc1	forkhead box C1	short mandible; small Meckel's cartilage; abnormal maxillary zygomatic process morphology; enlarged maxillary zygomatic process	4	210	0.019	FOXC1	forkhead box C1	HGNC:3800
Kdr	kinase insert domain protein receptor	abnormal mandibular prominence morphology	1	53	0.019	KDR	kinase insert domain receptor	HGNC:6307
Fgf8	fibroblast growth factor 8	abnormal maxilla morphology; abnormal mandible morphology; absent Meckel's cartilage	3	161	0.019	FGF8	fibroblast growth factor 8	HGNC:3686
Pax3	paired box 3	absent premaxilla; maxilla hypoplasia; abnormal premaxilla morphology	3	161	0.019	PAX3	paired box 3	HGNC:8617
Atp11a	ATPase, class VI, type 11A	abnormal Meckel's cartilage morphology	1	54	0.019	ATP11A	ATPase phospholipid transporting 11A	HGNC:13552
Cep290	centrosomal protein 290	micrognathia	1	54	0.019	CEP290	centrosomal protein 290	HGNC:29021
Dnase1l2	deoxyribonuclease 1-like 2	abnormal maxilla morphology	1	54	0.019	DNASE1L2	deoxyribonuclease 1 like 2	HGNC:2958
Wnt3a	wingless-type MMTV integration site family, member 3A	short mandible	1	54	0.019	WNT3A	Wnt family member 3A	HGNC:15983
Alkbh1	alkB homolog 1, histone H2A dioxygenase	abnormal mandible morphology	1	55	0.018	ALKBH1	alkB homolog 1, histone H2A dioxygenase	HGNC:17911
Pthlh	parathyroid hormone-like peptide	short mandible; abnormal mandible morphology	2	110	0.018	PTHLH	parathyroid hormone like hormone	HGNC:9607
Map3k7	mitogen-activated protein kinase kinase kinase 7	short mandible; short maxilla	2	112	0.018	MAP3K7	mitogen-activated protein kinase kinase kinase 7	HGNC:6859
Plxnd1	plexin D1	micrognathia	1	57	0.018	PLXND1	plexin D1	HGNC:9107
Abhd5	abhydrolase domain containing 5	abnormal maxilla morphology	1	58	0.017	ABHD5	abhydrolase domain containing 5, lysophosphatidic acid acyltransferase	HGNC:21396
Atmin	ATM interactor	micrognathia	1	59	0.017	ATMIN	ATM interactor	HGNC:29034
Cbfb	core binding factor beta	abnormal maxilla morphology; abnormal mandible morphology	2	119	0.017	CBFB	core-binding factor subunit beta	HGNC:1539
Ids	iduronate 2-sulfatase	abnormal mandible morphology	1	61	0.016	IDS	iduronate 2-sulfatase	HGNC:5389
Pax1	paired box 1	abnormal jaw morphology	1	62	0.016	PAX1	paired box 1	HGNC:8615
Pole4	polymerase (DNA-directed), epsilon 4 (p12 subunit)	short mandible	1	62	0.016	POLE4	DNA polymerase epsilon 4, accessory subunit	HGNC:18755
Egfr	epidermal growth factor receptor	short mandible; mandible hypoplasia; small Meckel's cartilage	3	188	0.016	EGFR	epidermal growth factor receptor	HGNC:3236

Ank	progressive ankylosis	mandibular hyperostosis	1	63	0.016	ANKH	ANKH inorganic pyrophosphate transport regulator	HGNC:15492
Disp1	dispatched RND transporter family member 1	absent premaxilla	1	63	0.016	DISP1	dispatched RND transporter family member 1	HGNC:19711
Ihh	Indian hedgehog	short mandible	1	64	0.016	IHH	Indian hedgehog signaling molecule	HGNC:5956
Pth1r	parathyroid hormone 1 receptor	short mandible	1	64	0.016	PTH1R	parathyroid hormone 1 receptor	HGNC:9608
Srf	serum response factor	mandible hypoplasia	1	64	0.016	SRF	serum response factor	HGNC:11291
Fgf9	fibroblast growth factor 9	short premaxilla; enlarged Meckel's cartilage	2	129	0.016	FGF9	fibroblast growth factor 9	HGNC:3687
Kat6a	K(lysine) acetyltransferase 6A	micrognathia	1	65	0.015	KAT6A	lysine acetyltransferase 6A	HGNC:13013
Megf8	multiple EGF-like-domains 8	micrognathia	1	65	0.015	MEGF8	multiple EGF like domains 8	HGNC:3233
Nfatc1	nuclear factor of activated T cells, cytoplasmic, calcineurin dependent 1	abnormal mandibular condyloid process morphology	1	66	0.015	NFATC1	nuclear factor of activated T cells 1	HGNC:7775
Sufu	SUFU negative regulator of hedgehog signaling	micrognathia	1	66	0.015	SUFU	SUFU negative regulator of hedgehog signaling	HGNC:16466
Bcl11b	B cell leukemia/lymphoma 11B	short maxilla	1	67	0.015	BCL11B	BAF chromatin remodeling complex subunit BCL11B	HGNC:13222
Sh3bp2	SH3-domain binding protein 2	abnormal jaw morphology	1	67	0.015	SH3BP2	SH3 domain binding protein 2	HGNC:10825
Ndst1	N-deacetylase/N-sulfotransferase (heparan glucosaminyl) 1	absent mandible	1	68	0.015	NDST1	N-deacetylase and N-sulfotransferase 1	HGNC:7680
Tbx1	T-box 1	short mandible; micrognathia; absent mandibular coronoid process	3	213	0.014	TBX1	T-box transcription factor 1	HGNC:11592
Trp63	transformation related protein 63	small maxilla; small mandible	2	142	0.014	TP63	tumor protein p63	HGNC:15979
Trp53bp2	transformation related protein 53 binding protein 2	micrognathia	1	72	0.014	TP53BP2	tumor protein p53 binding protein 2	HGNC:12000
Wdr11	WD repeat domain 11	small mandible	1	72	0.014	WDR11	WD repeat domain 11	HGNC:13831
Foxh1	forkhead box H1	absent mandible	1	74	0.014	FOXH1	forkhead box H1	HGNC:3814
Fgfrl1	fibroblast growth factor receptor-like 1	mandible hypoplasia	1	75	0.013	FGFRL1	fibroblast growth factor receptor like 1	HGNC:3693
Dnah5	dynein, axonemal, heavy chain 5	micrognathia	1	76	0.013	DNAH5	dynein axonemal heavy chain 5	HGNC:2950
Egr2	early growth response 2	abnormal mandible morphology	1	76	0.013	EGR2	early growth response 2	HGNC:3239
Dnah11	dynein, axonemal, heavy chain 11	micrognathia	1	79	0.013	DNAH11	dynein axonemal heavy chain 11	HGNC:2942
Fgfr1	fibroblast growth factor receptor 1	short mandible; abnormal maxillary-premaxillary suture morphology; premature maxillary-premaxillary suture closure	3	245	0.012	FGFR1	fibroblast growth factor receptor 1	HGNC:3688

Smarca4	SWI/SNF related, matrix associated, actin dependent regulator of chromatin, subfamily a, member 4	micrognathia	1	83	0.012	SMARCA 4	SWI/SNF related, matrix associated, actin dependent regulator of chromatin, subfamily a, member 4	HGNC:11100
Lmna	lamin A	short maxilla; mandible hypoplasia; micrognathia	3	251	0.012	LMNA	lamin A/C	HGNC:6636
Hoxa2	homeobox A2	abnormal Meckel's cartilage morphology	1	84	0.012	HOXA2	homeobox A2	HGNC:5103
Setd5	SET domain containing 5	abnormal maxilla morphology	1	89	0.011	SETD5	SET domain containing 5	HGNC:25566
Ptpn11	protein tyrosine phosphatase, non-receptor type 11	absent mandible; abnormal mandible morphology; micrognathia	3	273	0.011	PTPN11	protein tyrosine phosphatase non-receptor type 11	HGNC:9644
Myh10	myosin, heavy polypeptide 10, non-muscle	micrognathia	1	94	0.011	MYH10	myosin heavy chain 10	HGNC:7568
Ednrb	endothelin receptor type B	maxillary shelf hypoplasia	1	96	0.010	EDNRB	endothelin receptor type B	HGNC:3180
Atr	ataxia telangiectasia and Rad3 related	micrognathia	1	98	0.010	ATR	ATR serine/threonine kinase	HGNC:882
Lyn	LYN proto-oncogene, Src family tyrosine kinase	abnormal maxilla morphology	1	99	0.010	LYN	LYN proto-oncogene, Src family tyrosine kinase	HGNC:6735
Chtop	chromatin target of PRMT1	abnormal maxilla morphology	1	100	0.010	CHTOP	chromatin target of PRMT1	HGNC:24511
Elp1	elongator complex protein 1	mandibular retrognathia	1	101	0.010	ELP1	elongator complex protein 1	HGNC:5959
Asxl1	additional sex combs like 1	mandible hypoplasia	1	102	0.010	ASXL1	ASXL transcriptional regulator 1	HGNC:18318
Pdpk1	3-phosphoinositide dependent protein kinase 1	short mandible	1	102	0.010	PDPK1	3-phosphoinositide dependent protein kinase 1	HGNC:8816
Idua	iduronidase, alpha-L	abnormal mandibular condyloid process morphology	1	103	0.010	IDUA	alpha-L-iduronidase	HGNC:5391
Rdh10	retinol dehydrogenase 10 (all-trans)	abnormal maxillary prominence morphology	1	107	0.009	RDH10	retinol dehydrogenase 10	HGNC:19975
Apaf1	apoptotic peptidase activating factor 1	abnormal maxilla morphology	1	109	0.009	APAF1	apoptotic peptidase activating factor 1	HGNC:576
Lrp2	low density lipoprotein receptor-related protein 2	micrognathia	1	109	0.009	LRP2	LDL receptor related protein 2	HGNC:6694
Apc	APC, WNT signaling pathway regulator	absent mandible; abnormal mandible morphology	2	224	0.009	APC	APC regulator of WNT signaling pathway	HGNC:583
Nog	noggin	abnormal mandible morphology	1	112	0.009	NOG	noggin	HGNC:7866
Gja1	gap junction protein, alpha 1	small maxilla; small mandible	2	226	0.009	GJA1	gap junction protein alpha 1	HGNC:4274
B9d2	B9 protein domain 2	abnormal maxilla morphology	1	114	0.009	B9D2	B9 domain containing 2	HGNC:28636
Pdgfrb	platelet derived growth factor receptor, beta polypeptide	micrognathia	1	116	0.009	PDGFRB	platelet derived growth factor receptor beta	HGNC:8804
Tnfsf11	tumor necrosis factor (ligand) superfamily, member 11	mandible hypoplasia	1	117	0.009	TNFSF11	TNF superfamily member 11	HGNC:11926
Sh3pxd2a	SH3 and PX domains 2A	fragmented Meckel's cartilage	1	123	0.008	SH3PXD2 A	SH3 and PX domains 2A	HGNC:23664
Hspg2	perlecan (heparan sulfate proteoglycan 2)	short mandible	1	127	0.008	HSPG2	heparan sulfate proteoglycan 2	HGNC:5273

Chuk	conserved helix-loop-helix ubiquitous kinase	short mandible	1	128	0.008	CHUK	component of inhibitor of nuclear factor kappa B kinase complex	HGNC:1974
Itgb1	integrin beta 1 (fibronectin receptor beta)	mandible hypoplasia; micrognathia	2	262	0.008	ITGB1	integrin subunit beta 1	HGNC:6153
Jag1	jagged 1	short maxilla	1	131	0.008	JAG1	jagged canonical Notch ligand 1	HGNC:6188
Vegfa	vascular endothelial growth factor A	short mandible; abnormal mandible morphology	2	262	0.008	VEGFA	vascular endothelial growth factor A	HGNC:12680
Mecp2	methyl CpG binding protein 2	abnormal jaw morphology	1	139	0.007	MECP2	methyl-CpG binding protein 2	HGNC:6990
Fbn1	fibrillin 1	long mandible	1	141	0.007	FBN1	fibrillin 1	HGNC:3603
Fgf10	fibroblast growth factor 10	palatal shelf fusion with tongue or mandible	1	145	0.007	FGF10	fibroblast growth factor 10	HGNC:3666
Shh	sonic hedgehog	abnormal maxilla morphology; abnormal maxillary shelf morphology	2	295	0.007	SHH	sonic hedgehog signaling molecule	HGNC:10848
Col1a1	collagen, type I, alpha 1	abnormal maxilla morphology	1	157	0.006	COL1A1	collagen type I alpha 1 chain	HGNC:2197
Pax6	paired box 6	abnormal maxillary shelf morphology	1	175	0.006	PAX6	paired box 6	HGNC:8620
Gli3	GLI-Kruppel family member GLI3	abnormal maxilla morphology	1	219	0.005	GLI3	GLI family zinc finger 3	HGNC:4319
Braf	Braf transforming gene	mandible hypoplasia	1	228	0.004	BRAF	B-Raf proto-oncogene, serine/threonine kinase	HGNC:1097
Trp53	transformation related protein 53	short mandible	1	292	0.003	TP53	tumor protein p53	HGNC:11998

Supplementary Table 3: Associations of each JIG with genetic and epigenetic alterations in tumors, survival of PanCan

CGC	included in the Cancer Gene Census list
Cancer driver	included among the cancer driver genes in PanCan
Freqmut	included among the frequently mutated genes in PanCan
Methylation	differentially methylated JIGs in human tumors versus normal tissue, from DiseaseMeth database (hypo = hypomethylated, hyper = hypermethylated)
CCLE	up- or down-regulated in highly-invasive vs. less-invasive human cells from the CCLE
Survival	impact on the survival prognosis of the patients from the PanCancer cohort based on Cox regression analysis
Hsa Metastases	up- or down-regulated in human metastasic vs. primary lesions (GSE21510, GSE2509, GSE25976, GSE43837, GSE468, GSE6919, GSE7929, GSE7930, GSE8401)
Mmu metast driver	included among metastasis driver genes in a mouse model of tumor evolution

Gene name	Gene ID	Evolutionary origin	Hub	CGC	Cancer driver	Freqmut	Methylation	CCLE	Survival	Hsa metastases	Mmu metast driver
ABHD5	ENSG00000011198	gnathostome	No	No	No	No	-	-	insignificant	UP/DN	No
ACVR1	ENSG00000115170	pre-gnathostome	No	Yes	No	No	hypo	UP	insignificant	UP/DN	No
ACVR2A	ENSG00000121989	gnathostome	No	Yes	Yes	Yes	hypo	-	favorable	UP/DN	No
ALDH1A2	ENSG00000128918	pre-gnathostome	No	No	No	No	hyper	-	insignificant	UP	No
ALKBH1	ENSG00000100601	pre-gnathostome	No	No	No	No	hypo	-	favorable	-	No
ALX1	ENSG00000180318	gnathostome	No	No	No	No	hyper	UP	poor	DN	No
AMBN	ENSG00000178522	gnathostome	Yes	No	No	No	hypo	-	poor	-	No
AMELX	ENSG00000125363	gnathostome	Yes	No	No	No	hypo/hyper	-	poor	UP	No
ANKH	ENSG00000154122	gnathostome	No	No	No	No	hypo	UP	favorable	DN	No
AP2B1	ENSG00000006125	pre-gnathostome	Yes	No	No	No	hypo	-	favorable	UP/DN	No
APAF1	ENSG00000120868	pre-gnathostome	No	No	No	No	hypo	-	poor	UP	No
APC	ENSG00000134982	pre-gnathostome	No	Yes	Yes	Yes	-	DN	poor	UP/DN	No
ARHGAP29	ENSG00000137962	gnathostome	No	No	No	No	hyper	UP/DN	favorable	UP/DN	No
ARID1A	ENSG00000117713	gnathostome	No	Yes	Yes	Yes	-	-	poor	UP/DN	No
ARID5B	ENSG00000150347	gnathostome	No	No	No	Yes	-	UP	favorable	UP/DN	No
ARSB	ENSG00000113273	pre-gnathostome	No	No	No	No	-	UP	poor	-	No
ASAP1	ENSG00000153317	pre-gnathostome	No	No	No	No	hypo/hyper	UP	poor	UP/DN	No
ASXL1	ENSG00000171456	gnathostome	No	Yes	Yes	Yes	hypo	-	poor	-	No
ATMIN	ENSG00000166454	pre-gnathostome	No	No	No	No	hypo	-	favorable	UP	No
ATP11A	ENSG00000068650	gnathostome	No	No	No	No	hypo	DN	poor	UP/DN	No
ATPAF2	ENSG00000171953	pre-gnathostome	No	No	No	No	-	-	favorable	DN	No
ATR	ENSG00000175054	pre-gnathostome	No	Yes	Yes	Yes	hypo	-	poor	UP	No
B9D2	ENSG00000123810	pre-gnathostome	No	No	No	No	-	-	favorable	-	No
BAZ1B	ENSG00000009954	pre-gnathostome	No	No	No	No	hypo	-	poor	DN	No
BCL11B	ENSG00000127152	gnathostome	No	Yes	No	No	hypo/hyper	UP/DN	poor	UP/DN	No
BMP4	ENSG00000125378	gnathostome	Yes	No	No	No	hypo/hyper	DN	favorable	DN	No
BMP5	ENSG00000112175	gnathostome	No	Yes	No	No	hypo	DN	insignificant	UP/DN	No
BMP7	ENSG00000101144	gnathostome	No	No	No	No	hyper	UP/DN	insignificant	UP/DN	No
BNC2	ENSG00000173068	pre-gnathostome	No	No	No	No	hyper	UP	insignificant	DN	No
BRAF	ENSG00000157764	pre-gnathostome	Yes	Yes	Yes	Yes	-	-	favorable	-	No
BRD4	ENSG00000141867	gnathostome	No	Yes	No	No	hypo/hyper	-	poor	UP	No
CACNA1S	ENSG00000081248	gnathostome	No	No	No	No	hypo	-	poor	DN	No
CASK	ENSG00000147044	pre-gnathostome	No	No	No	No	hyper	-	poor	UP/DN	No
CBFB	ENSG00000067955	pre-gnathostome	Yes	Yes	Yes	Yes	hypo	-	poor	UP/DN	No
CCDC39	ENSG00000284862	pre-gnathostome	No	No	No	No	hypo/hyper	-	-	-	No
CCN2	ENSG00000118523	pre-gnathostome	No	No	No	No	hypo	UP	favorable	-	No
CCND1	ENSG00000110092	gnathostome	Yes	Yes	Yes	Yes	hypo	-	favorable	UP/DN	No

CDC73	ENSG00000134371	pre-gnathostome	No	Yes	No	No	-	-	poor	UP/DN	No
CDK10	ENSG00000185324	pre-gnathostome	No	No	No	No	-	-	favorable	UP/DN	No
CDON	ENSG00000064309	gnathostome	Yes	No	No	No	hypo	UP	favorable	-	No
CEP290	ENSG00000198707	pre-gnathostome	No	No	No	No	hypo	-	favorable	-	No
CHD1	ENSG00000153922	gnathostome	No	No	No	No	-	-	insignificant	-	No
CHRD	ENSG00000090539	pre-gnathostome	No	No	No	No	-	UP/DN	favorable	DN	No
CHST11	ENSG00000171310	gnathostome	No	Yes	No	No	hypo/hyper	UP	poor	DN	No
CHTOP	ENSG00000160679	pre-gnathostome	No	No	No	No	hypo	-	poor	UP	No
CHUK	ENSG00000213341	gnathostome	No	No	No	No	-	-	insignificant	UP	No
CNBP	ENSG00000169714	pre-gnathostome	No	Yes	No	No	hypo	-	favorable	-	No
COL11A1	ENSG00000060718	gnathostome	No	No	No	No	hypo/hyper	UP	poor	UP/DN	No
COL1A1	ENSG00000108821	gnathostome	No	Yes	No	No	hyper	UP	poor	UP/DN	No
COL27A1	ENSG00000196739	gnathostome	No	No	No	No	hypo	UP	insignificant	UP/DN	No
COL2A1	ENSG00000139219	pre-gnathostome	No	Yes	No	No	-	DN	insignificant	DN	No
CPLANE1	ENSG00000197603	pre-gnathostome	No	No	No	No	-	UP	insignificant	-	No
CPLANE2	ENSG00000132881	pre-gnathostome	No	No	No	No	-	-	favorable	-	No
CREBBP	ENSG00000005339	gnathostome	Yes	Yes	Yes	No	hypo	-	poor	DN	No
CRIM1	ENSG00000150938	pre-gnathostome	No	No	No	No	hypo	UP	favorable	DN	Yes
CRKL	ENSG00000099942	pre-gnathostome	No	No	No	No	-	-	poor	UP	No
CTNNB1	ENSG00000168036	gnathostome	Yes	Yes	Yes	Yes	hypo	-	insignificant	UP	No
CTNNBIP1	ENSG00000178585	gnathostome	No	No	No	No	hypo	DN	insignificant	UP/DN	No
CTSK	ENSG00000143387	gnathostome	No	No	No	No	hypo/hyper	UP	poor	UP/DN	No
CYB561	ENSG00000008283	pre-gnathostome	No	No	No	No	hypo	DN	favorable	UP/DN	No
CYP26B1	ENSG00000003137	pre-gnathostome	No	No	No	No	hypo	-	insignificant	DN	No
CYP51A1	ENSG00000001630	pre-gnathostome	No	No	No	No	hypo	-	poor	-	No
DAAM2	ENSG00000146122	pre-gnathostome	No	No	No	No	hypo/hyper	UP	insignificant	UP/DN	No
DCTN5	ENSG00000166847	pre-gnathostome	No	No	No	No	hypo	-	insignificant	UP/DN	No
DHRS3	ENSG00000162496	pre-gnathostome	No	No	No	No	hypo/hyper	-	favorable	UP/DN	No
DISP1	ENSG00000154309	gnathostome	No	No	No	No	hypo	DN	favorable	UP	No
DKK1	ENSG00000107984	gnathostome	No	No	No	No	hyper	UP	poor	UP/DN	Yes
DLG1	ENSG00000075711	gnathostome	No	No	No	No	hypo	-	poor	UP/DN	No
DLX2	ENSG00000115844	gnathostome	No	No	No	No	hyper	UP/DN	poor	UP	No
DLX5	ENSG00000105880	gnathostome	No	No	No	No	hyper	-	insignificant	UP/DN	Yes
DLX6	ENSG00000006377	gnathostome	No	No	No	No	-	-	insignificant	-	No
DNAH11	ENSG00000105877	gnathostome	No	No	No	No	hypo/hyper	UP	insignificant	-	No
DNAH5	ENSG00000039139	pre-gnathostome	No	No	No	No	hypo	-	favorable	-	No
DNAI1	ENSG00000122735	pre-gnathostome	No	No	No	No	hypo	DN	favorable	UP	No
DNASE1L2	ENSG00000167968	pre-gnathostome	No	No	No	No	-	DN	favorable	UP/DN	No
DOCK1	ENSG00000150760	gnathostome	No	No	No	No	hypo	UP	favorable	DN	No
DPH1	ENSG00000108963	pre-gnathostome	No	No	No	No	-	-	favorable	-	No
DRC1	ENSG00000157856	pre-gnathostome	No	No	No	No	hyper	-	poor	-	No
DVL3	ENSG00000161202	gnathostome	No	No	No	No	hypo	-	poor	-	No
DYNC2H1	ENSG00000187240	pre-gnathostome	Yes	No	No	No	hypo	UP	favorable	DN	No
ECE1	ENSG00000117298	gnathostome	No	No	No	No	hypo	-	favorable	DN	No
EDN1	ENSG00000078401	pre-gnathostome	Yes	No	No	No	-	-	insignificant	DN	Yes
EDNRA	ENSG00000151617	pre-gnathostome	Yes	No	No	No	hyper	UP	favorable	UP/DN	No
EDNRB	ENSG00000136160	pre-gnathostome	No	No	No	No	hypo/hyper	UP	favorable	DN	No
EGFR	ENSG00000146648	gnathostome	Yes	Yes	Yes	Yes	-	UP/DN	insignificant	UP/DN	Yes
EGR2	ENSG00000122877	gnathostome	No	No	No	No	hypo	UP	favorable	DN	No

ELP1	ENSG00000070061	pre-gnathostome	No	No	No	No	-	-	insignificant	-	No
EPS8L3	ENSG00000198758	pre-gnathostome	No	No	No	No	hypo	-	poor	UP	No
EYA1	ENSG00000104313	gnathostome	No	No	No	No	hypo	UP	poor	UP/DN	No
FAM20C	ENSG00000177706	pre-gnathostome	No	No	No	No	hypo/hyper	UP	poor	DN	No
FBLN1	ENSG00000077942	gnathostome	No	No	No	No	-	DN	insignificant	UP/DN	No
FBN1	ENSG00000166147	gnathostome	Yes	No	No	No	hyper	UP	insignificant	UP/DN	No
FBN2	ENSG00000138829	pre-gnathostome	Yes	No	No	No	hyper	UP	poor	DN	No
FGF10	ENSG00000070193	pre-gnathostome	Yes	No	No	No	hypo/hyper	-	insignificant	-	No
FGF18	ENSG00000156427	gnathostome	Yes	No	No	No	-	DN	favorable	DN	No
FGF8	ENSG00000107831	gnathostome	Yes	No	No	No	hyper	-	poor	-	No
FGF9	ENSG00000102678	gnathostome	Yes	No	No	No	-	DN	insignificant	UP/DN	No
FGFR1	ENSG00000077782	gnathostome	No	Yes	Yes	No	hypo	UP	favorable	DN	Yes
FGFR2	ENSG00000066468	gnathostome	Yes	Yes	Yes	Yes	hypo/hyper	DN	favorable	UP/DN	No
FGFR3	ENSG00000068078	gnathostome	Yes	Yes	Yes	Yes	-	DN	poor	UP/DN	No
FGFRL1	ENSG00000127418	pre-gnathostome	No	No	No	No	hypo	UP	favorable	-	No
FIGN	ENSG00000182263	gnathostome	No	No	No	No	hyper	UP/DN	poor	UP/DN	No
FLVCR1	ENSG00000162769	gnathostome	No	No	No	No	hypo	DN	poor	UP	No
FOXC1	ENSG00000054598	gnathostome	No	No	No	No	-	-	poor	DN	No
FOXC2	ENSG00000176692	gnathostome	No	No	No	No	hyper	UP	poor	UP	No
FOXD3	ENSG00000187140	pre-gnathostome	No	No	No	No	-	-	poor	DN	No
FOXF2	ENSG00000137273	gnathostome	No	No	No	No	-	UP	poor	UP	No
FOXH1	ENSG00000160973	pre-gnathostome	No	No	No	No	-	-	poor	UP	No
FOXI3	ENSG00000214336	gnathostome	No	No	No	No	hypo/hyper	-	poor	-	No
FOXN3	ENSG00000053254	gnathostome	No	No	No	No	hypo/hyper	UP	poor	DN	No
FRAS1	ENSG00000138759	pre-gnathostome	No	No	No	No	hypo	DN	favorable	-	No
FREM1	ENSG00000164946	pre-gnathostome	No	No	No	No	hyper	-	favorable	DN	No
FUZ	ENSG00000010361	pre-gnathostome	No	No	No	No	hypo	-	favorable	-	No
GAD1	ENSG00000128683	pre-gnathostome	No	No	No	No	-	DN	poor	-	No
GAS1	ENSG00000180447	pre-gnathostome	Yes	No	No	No	hypo	UP	poor	DN	Yes
GAS2L2	ENSG00000270765	gnathostome	No	No	No	No	hypo	-	poor	-	No
GJA1	ENSG00000152661	gnathostome	No	No	No	No	-	UP	insignificant	UP/DN	No
GJB2	ENSG00000165474	pre-gnathostome	No	No	No	No	-	DN	poor	DN	Yes
GKN1	ENSG00000169605	gnathostome	No	No	No	No	hypo	-	poor	-	No
GLDC	ENSG00000178445	pre-gnathostome	No	No	No	No	hypo/hyper	UP	poor	UP	No
GLG1	ENSG00000090863	pre-gnathostome	No	No	No	No	hypo	UP	insignificant	UP	No
GLI2	ENSG00000074047	gnathostome	Yes	No	No	No	hypo/hyper	UP	poor	DN	No
GLI3	ENSG00000106571	gnathostome	Yes	No	No	No	hyper	UP	poor	DN	No
GPC3	ENSG00000147257	gnathostome	Yes	Yes	No	No	-	DN	poor	-	No
GRB2	ENSG00000177885	pre-gnathostome	Yes	No	No	No	hypo	-	insignificant	UP/DN	No
GREM2	ENSG00000180875	gnathostome	No	No	No	No	hypo/hyper	UP	insignificant	DN	No
GSC	ENSG00000133937	pre-gnathostome	No	No	No	No	hypo/hyper	UP	insignificant	-	No
HAND1	ENSG00000113196	gnathostome	No	No	No	No	-	-	poor	UP	No
HAND2	ENSG00000164107	gnathostome	No	No	No	No	hyper	UP	poor	-	No
HAPLN1	ENSG00000145681	gnathostome	No	No	No	No	-	UP	insignificant	UP/DN	No
HAT1	ENSG00000128708	pre-gnathostome	No	No	No	No	hypo	-	poor	UP	No
HHAT	ENSG00000054392	pre-gnathostome	No	No	No	No	-	UP/DN	favorable	DN	No
HOXA2	ENSG00000105996	gnathostome	No	No	No	No	-	UP	poor	-	No
HOXA3	ENSG00000105997	gnathostome	No	No	No	No	hypo/hyper	UP	poor	UP	No
HRAS	ENSG00000174775	gnathostome	Yes	Yes	Yes	No	hypo	-	insignificant	UP/DN	No

HSPB11	ENSG00000081870	pre-gnathostome	Yes	No	No	No	hypo	-	poor	UP/DN	No
HSPG2	ENSG00000142798	pre-gnathostome	No	No	No	No	hypo	UP	poor	UP/DN	No
IBSP	ENSG00000029559	gnathostome	No	No	No	No	hypo	-	poor	-	No
IDE	ENSG00000119912	pre-gnathostome	No	No	No	No	-	-	favorable	UP/DN	No
IDS	ENSG00000010404	pre-gnathostome	No	No	No	No	hypo/hyper	-	insignificant	UP/DN	No
IDUA	ENSG00000127415	gnathostome	No	No	No	No	hypo	-	insignificant	UP	No
IFITM5	ENSG00000206013	gnathostome	No	No	No	No	hypo	-	poor	-	No
IFT140	ENSG00000187535	pre-gnathostome	No	No	No	No	hypo	-	favorable	DN	No
IFT27	ENSG00000100360	pre-gnathostome	No	No	No	No	hypo	-	favorable	-	No
IFT57	ENSG00000114446	pre-gnathostome	Yes	No	No	No	hypo	UP	favorable	UP	No
IHH	ENSG00000163501	gnathostome	Yes	No	No	No	hypo	-	favorable	UP/DN	No
IRF6	ENSG00000117595	gnathostome	No	No	Yes	No	hypo/hyper	DN	favorable	UP/DN	Yes
ITGB1	ENSG00000150093	gnathostome	Yes	No	No	No	hypo	UP	poor	UP/DN	No
JAG1	ENSG00000101384	pre-gnathostome	No	No	No	No	hypo	DN	poor	UP/DN	Yes
JAG2	ENSG00000184916	gnathostome	No	No	No	No	hyper	DN	favorable	DN	No
KAT14	ENSG00000149474	pre-gnathostome	No	No	No	No	-	-	favorable	UP/DN	No
KAT6A	ENSG00000083168	gnathostome	No	Yes	No	No	hypo	-	poor	UP	No
KAT6B	ENSG00000156650	gnathostome	No	Yes	No	No	hypo	-	favorable	UP/DN	No
KCNJ13	ENSG00000115474	pre-gnathostome	No	No	No	No	hypo/hyper	-	insignificant	DN	No
KCNJ2	ENSG00000123700	gnathostome	No	No	No	No	hyper	UP/DN	favorable	UP	No
KDF1	ENSG00000175707	gnathostome	No	No	No	No	hypo	-	favorable	-	Yes
KDR	ENSG00000128052	gnathostome	Yes	Yes	No	No	hyper	DN	favorable	-	No
KIF3A	ENSG00000131437	gnathostome	Yes	No	No	No	-	-	favorable	UP	No
KLF2	ENSG00000127528	gnathostome	No	No	No	No	-	UP	insignificant	UP/DN	No
KMT2D	ENSG00000167548	gnathostome	Yes	Yes	Yes	No	hypo/hyper	-	poor	UP/DN	No
LIMK2	ENSG00000182541	gnathostome	No	No	No	No	hypo	DN	insignificant	UP/DN	No
LMNA	ENSG00000160789	pre-gnathostome	No	Yes	No	No	hypo	UP	poor	DN	No
LOXL3	ENSG00000115318	gnathostome	No	No	No	No	-	UP	poor	DN	No
LPAR4	ENSG00000147145	gnathostome	No	No	No	No	-	UP	poor	-	No
LRP2	ENSG00000081479	pre-gnathostome	Yes	No	No	No	hypo	DN	favorable	-	No
LRRK1	ENSG00000154237	pre-gnathostome	No	No	No	No	hyper	UP/DN	poor	DN	No
LTBP1	ENSG00000049323	pre-gnathostome	No	No	No	No	hypo/hyper	UP/DN	poor	UP/DN	No
LTBP3	ENSG00000168056	gnathostome	No	No	No	No	-	UP	favorable	UP/DN	No
LYN	ENSG00000254087	gnathostome	Yes	No	No	No	hypo/hyper	UP/DN	poor	UP/DN	No
MAP3K7	ENSG00000135341	pre-gnathostome	No	No	No	No	hypo	-	poor	UP/DN	No
MAPK1	ENSG00000100030	gnathostome	Yes	Yes	Yes	No	hypo	-	insignificant	UP/DN	No
MAU2	ENSG00000129933	pre-gnathostome	No	No	No	No	hypo	-	poor	DN	No
MBTPS1	ENSG00000140943	pre-gnathostome	Yes	No	No	No	hypo	-	favorable	DN	No
MECP2	ENSG00000169057	pre-gnathostome	No	No	No	No	hyper	-	favorable	DN	No
MEF2C	ENSG00000081189	gnathostome	Yes	No	No	No	hyper	UP	poor	DN	No
MEGF8	ENSG00000105429	pre-gnathostome	No	No	No	No	hypo	-	insignificant	UP/DN	No
MFSD12	ENSG00000161091	pre-gnathostome	No	No	No	No	-	-	poor	UP/DN	No
MIGA2	ENSG00000148343	gnathostome	No	No	No	No	-	-	favorable	-	No
MKS1	ENSG00000011143	pre-gnathostome	Yes	No	No	No	hypo	-	favorable	-	No
MMP14	ENSG00000157227	gnathostome	No	No	No	No	hypo/hyper	UP	poor	DN	Yes
MMP2	ENSG00000087245	gnathostome	Yes	No	No	No	hyper	UP	poor	UP/DN	No
MMP21	ENSG00000154485	gnathostome	No	No	No	No	hypo	-	insignificant	-	No
MN1	ENSG00000169184	gnathostome	No	Yes	No	No	hypo	UP	poor	DN	No
MNT	ENSG00000070444	pre-gnathostome	No	No	No	No	hypo	-	poor	UP	No

MORC2	ENSG00000133422	pre-gnathostome	No	No	No	No	hypo	-	poor	UP	No
MSX1	ENSG00000163132	gnathostome	No	No	No	No	hypo/hyper	UP/DN	favorable	UP/DN	Yes
MTHFD1L	ENSG00000120254	pre-gnathostome	No	No	No	No	hypo	UP	poor	UP/DN	No
MYH10	ENSG00000133026	pre-gnathostome	No	No	No	No	-	UP	insignificant	UP/DN	No
NABP2	ENSG00000139579	gnathostome	No	No	No	No	hypo	-	poor	DN	No
NDST1	ENSG00000070614	gnathostome	No	No	No	No	-	-	insignificant	UP/DN	No
NFATC1	ENSG00000131196	gnathostome	No	No	No	No	hypo/hyper	DN	poor	UP	Yes
NFIC	ENSG00000141905	gnathostome	No	No	No	No	hypo/hyper	UP	favorable	DN	No
NIPBL	ENSG00000164190	pre-gnathostome	No	No	Yes	No	hypo	-	poor	UP/DN	No
NISCH	ENSG00000010322	pre-gnathostome	No	No	No	No	-	-	favorable	DN	No
NOG	ENSG00000183691	pre-gnathostome	No	No	No	No	-	UP	poor	-	No
NPR2	ENSG00000159899	gnathostome	No	No	No	No	hypo/hyper	UP	poor	UP/DN	No
OSTM1	ENSG00000081087	pre-gnathostome	No	No	No	No	hypo	UP	poor	UP	No
OTULIN	ENSG00000154124	pre-gnathostome	No	No	No	No	hypo	-	poor	UP	No
OTX2	ENSG00000165588	gnathostome	No	No	No	No	hypo/hyper	-	poor	-	No
PAK1IP1	ENSG00000111845	pre-gnathostome	No	No	No	No	-	-	insignificant	UP/DN	No
PAPPA2	ENSG00000116183	gnathostome	No	No	No	No	hypo	UP	poor	-	No
PAX1	ENSG00000125813	gnathostome	No	No	No	No	hypo/hyper	-	insignificant	-	No
PAX3	ENSG00000135903	gnathostome	No	Yes	No	No	hyper	-	poor	-	No
PAX6	ENSG00000007372	pre-gnathostome	No	No	No	No	hyper	-	poor	DN	No
PAX7	ENSG00000009709	gnathostome	No	Yes	No	No	hyper	-	poor	DN	No
PAX9	ENSG00000198807	gnathostome	No	No	No	No	hyper	DN	favorable	-	No
PCSK6	ENSG00000140479	gnathostome	No	No	No	No	-	UP/DN	insignificant	UP	No
PDGFRB	ENSG00000113721	gnathostome	Yes	Yes	No	No	-	UP	insignificant	DN	No
PDPK1	ENSG00000140992	pre-gnathostome	No	No	No	No	hypo	-	favorable	UP	No
PDS5B	ENSG00000083642	pre-gnathostome	No	No	No	No	hypo	-	insignificant	UP/DN	No
PDSS2	ENSG00000164494	pre-gnathostome	No	No	No	No	hypo	-	favorable	DN	No
PFAS	ENSG00000178921	pre-gnathostome	No	No	No	No	hypo	UP	poor	UP	No
PGAP1	ENSG00000197121	pre-gnathostome	No	No	No	No	hypo	UP/DN	poor	UP/DN	No
PHEX	ENSG00000102174	pre-gnathostome	No	No	No	No	hypo	DN	insignificant	-	No
PIGV	ENSG00000060642	pre-gnathostome	No	No	No	No	-	-	favorable	DN	No
PITX1	ENSG00000069011	gnathostome	No	No	No	No	-	DN	poor	UP/DN	No
PITX2	ENSG00000164093	gnathostome	Yes	No	No	No	hyper	UP/DN	poor	UP	No
PKD1	ENSG00000008710	pre-gnathostome	No	No	No	No	hypo	UP	poor	DN	No
PKDCC	ENSG00000162878	pre-gnathostome	No	No	No	No	hyper	UP	favorable	-	No
PLXND1	ENSG00000004399	pre-gnathostome	No	No	No	No	hypo	-	poor	UP	No
POLE4	ENSG00000115350	pre-gnathostome	No	No	No	No	-	-	poor	-	No
POSTN	ENSG00000133110	gnathostome	No	No	No	No	hypo	UP	poor	DN	Yes
POU3F3	ENSG00000198914	gnathostome	No	No	No	No	hyper	UP	insignificant	DN	Yes
PRDM16	ENSG00000142611	gnathostome	No	Yes	No	No	hyper	UP	poor	-	No
PRICKLE1	ENSG00000139174	gnathostome	No	No	No	No	hypo/hyper	UP	favorable	DN	No
PRKRA	ENSG00000180228	gnathostome	No	No	No	No	hypo	-	poor	DN	No
PRRX1	ENSG00000116132	gnathostome	No	Yes	No	No	hypo/hyper	UP	insignificant	UP/DN	No
PSIP1	ENSG00000164985	pre-gnathostome	No	Yes	Yes	No	hyper	UP	poor	UP/DN	No
PSPH	ENSG00000146733	pre-gnathostome	No	No	No	No	-	-	poor	UP/DN	No
PTCH1	ENSG00000185920	gnathostome	Yes	Yes	Yes	No	hypo	-	insignificant	UP/DN	No
PTH1R	ENSG00000160801	gnathostome	No	No	No	No	hypo	UP	insignificant	DN	No
PTHLH	ENSG00000087494	pre-gnathostome	No	No	No	No	hypo/hyper	UP	poor	UP/DN	No
PTK7	ENSG00000112655	pre-gnathostome	No	No	No	No	hypo	-	insignificant	UP/DN	No

PTPN11	ENSG00000179295	gnathostome	Yes	Yes	Yes	Yes	hypo	-	poor	UP/DN	No
QSOX1	ENSG00000116260	gnathostome	Yes	No	No	No	-	UP	poor	UP/DN	No
RBBP7	ENSG00000102054	gnathostome	Yes	No	No	No	hyper	UP	favorable	-	No
RDH10	ENSG00000121039	pre-gnathostome	No	No	No	No	-	-	poor	-	No
RFNG	ENSG00000169733	gnathostome	No	No	No	No	-	-	insignificant	DN	Yes
ROBO1	ENSG00000169855	gnathostome	No	No	No	No	hyper	UP	poor	UP/DN	No
ROR2	ENSG00000169071	gnathostome	No	No	No	No	-	UP	favorable	UP/DN	No
RPGRIP1L	ENSG00000103494	pre-gnathostome	No	No	No	No	hypo	-	insignificant	UP/DN	No
RSPO2	ENSG00000147655	gnathostome	No	Yes	No	No	hypo/hyper	-	poor	DN	No
RYK	ENSG00000163785	pre-gnathostome	No	No	No	No	hypo	-	insignificant	UP	No
SATB2	ENSG00000119042	gnathostome	No	No	No	No	hyper	UP	insignificant	DN	No
SC5D	ENSG00000109929	pre-gnathostome	No	No	No	No	hypo	DN	favorable	UP/DN	No
SCHIP1	ENSG00000151967	pre-gnathostome	No	No	No	No	hypo/hyper	UP	poor	-	No
SETD5	ENSG00000168137	gnathostome	No	No	No	No	hypo	-	poor	UP/DN	No
SFN	ENSG00000175793	gnathostome	Yes	No	No	No	hypo	DN	insignificant	UP/DN	Yes
SH3BP2	ENSG00000087266	pre-gnathostome	No	No	No	No	hyper	-	poor	UP/DN	No
SH3PXD2A	ENSG00000107957	gnathostome	No	No	No	No	-	UP	poor	UP/DN	No
SH3PXD2B	ENSG00000174705	gnathostome	No	No	No	No	-	UP	poor	DN	No
SHH	ENSG00000164690	gnathostome	Yes	No	No	No	hypo	DN	insignificant	-	No
SHOX2	ENSG00000168779	pre-gnathostome	No	No	No	No	-	UP/DN	poor	UP	No
SIX1	ENSG00000126778	pre-gnathostome	No	Yes	No	No	hypo	UP	favorable	UP	No
SKI	ENSG00000157933	gnathostome	No	Yes	No	No	hypo	-	poor	UP	No
SLC10A7	ENSG00000120519	pre-gnathostome	No	No	No	No	-	-	insignificant	DN	No
SLC25A21	ENSG00000183032	pre-gnathostome	No	No	No	No	hypo/hyper	DN	favorable	-	No
SLC26A2	ENSG00000155850	pre-gnathostome	No	No	No	No	-	UP/DN	favorable	UP/DN	No
SLC27A4	ENSG00000167114	gnathostome	No	No	No	No	hypo	-	insignificant	UP/DN	No
SLC38A10	ENSG00000157637	pre-gnathostome	No	No	No	No	hypo	-	favorable	UP/DN	No
SLC39A13	ENSG00000165915	pre-gnathostome	No	No	No	No	hypo	UP	insignificant	-	No
SMAD2	ENSG00000175387	gnathostome	Yes	Yes	Yes	Yes	hypo	-	insignificant	UP/DN	No
SMARCA4	ENSG00000127616	gnathostome	Yes	Yes	Yes	No	hypo	-	insignificant	UP/DN	No
SMG1	ENSG00000157106	pre-gnathostome	No	No	No	No	hypo	-	poor	DN	No
SMO	ENSG00000128602	pre-gnathostome	Yes	Yes	No	No	hyper	UP	insignificant	UP/DN	No
SOSTDC1	ENSG00000171243	gnathostome	No	No	No	No	hypo	-	favorable	UP/DN	No
SOX11	ENSG00000176887	gnathostome	No	No	No	No	hyper	-	poor	UP	No
SOX9	ENSG00000125398	gnathostome	Yes	No	Yes	Yes	hypo	-	favorable	UP/DN	No
SPRY2	ENSG00000136158	gnathostome	Yes	No	No	No	hypo	UP	poor	UP/DN	No
SPRY4	ENSG00000187678	gnathostome	No	No	No	No	-	UP	insignificant	UP/DN	No
SRF	ENSG00000112658	pre-gnathostome	No	No	No	No	hypo	-	insignificant	UP/DN	No
SUFU	ENSG00000107882	pre-gnathostome	Yes	Yes	No	No	hypo	-	favorable	-	No
TBX1	ENSG00000184058	gnathostome	No	No	No	No	hyper	-	poor	UP/DN	No
TBX15	ENSG00000092607	gnathostome	No	No	No	No	hyper	UP	poor	-	No
TCOF1	ENSG00000070814	gnathostome	No	No	No	No	-	-	poor	UP/DN	No
TENT5A	ENSG00000112773	gnathostome	No	No	No	No	-	-	poor	-	No
TFAP2A	ENSG00000137203	pre-gnathostome	No	No	No	No	-	DN	poor	UP/DN	No
TGDS	ENSG00000088451	gnathostome	No	No	No	No	-	-	poor	UP/DN	No
TGFB2	ENSG00000092969	pre-gnathostome	No	No	No	No	hypo/hyper	UP	insignificant	DN	No
TGFBR2	ENSG00000163513	pre-gnathostome	No	Yes	Yes	Yes	hypo	UP	insignificant	UP/DN	Yes
TIPARP	ENSG00000163659	pre-gnathostome	No	No	No	No	hypo	UP/DN	poor	DN	Yes
TMEM67	ENSG00000164953	pre-gnathostome	No	No	No	No	hypo	-	poor	-	No

TNFRSF11B	ENSG00000164761	pre-gnathostome	No	No	No	No	-	UP	favorable	UP/DN	No
TNFSF11	ENSG00000120659	gnathostome	No	No	No	No	hyper	-	poor	UP/DN	No
TP53	ENSG00000141510	gnathostome	Yes	Yes	Yes	Yes	hypo	-	insignificant	DN	No
TP53BP2	ENSG00000143514	gnathostome	No	No	No	No	-	DN	poor	-	No
TP63	ENSG00000073282	gnathostome	No	Yes	No	No	hypo/hyper	DN	insignificant	DN	No
TRAPPC10	ENSG00000160218	pre-gnathostome	No	No	No	No	hypo	-	poor	UP	No
TRPS1	ENSG00000104447	pre-gnathostome	No	No	No	No	hyper	UP/DN	favorable	UP/DN	No
TTC28	ENSG00000100154	pre-gnathostome	No	No	No	No	hypo/hyper	UP	favorable	UP/DN	No
TWSG1	ENSG00000128791	pre-gnathostome	No	No	No	No	-	UP	poor	DN	No
VEGFA	ENSG00000112715	gnathostome	Yes	No	No	No	-	UP	insignificant	UP	No
VPS25	ENSG00000131475	pre-gnathostome	No	No	No	No	hypo	-	insignificant	DN	No
WDR11	ENSG00000120008	pre-gnathostome	No	No	No	No	-	-	favorable	UP/DN	No
WDR19	ENSG00000157796	pre-gnathostome	No	No	No	No	hypo	UP	favorable	-	No
WNT3A	ENSG00000154342	pre-gnathostome	Yes	No	No	No	hypo	DN	insignificant	-	No
WNT5A	ENSG00000114251	gnathostome	Yes	No	No	No	hypo/hyper	UP	insignificant	UP/DN	No
WWP2	ENSG00000198373	gnathostome	No	No	No	No	-	-	favorable	UP/DN	No
XYLT1	ENSG00000103489	pre-gnathostome	No	No	No	No	hypo	-	poor	DN	No
YBX1	ENSG00000065978	gnathostome	No	No	No	No	hypo	UP	poor	-	No
ZEB1	ENSG00000148516	gnathostome	No	Yes	No	No	hypo/hyper	UP	poor	UP/DN	No
ZIC3	ENSG00000156925	gnathostome	No	No	No	No	hypo	-	poor	DN	Yes
ZIC5	ENSG00000139800	gnathostome	No	No	No	No	hyper	DN	poor	UP	No
ZMPSTE24	ENSG00000084073	pre-gnathostome	No	No	No	No	hypo	-	insignificant	-	No
ZNF366	ENSG00000178175	pre-gnathostome	No	No	No	No	-	-	favorable	-	No
			60	51	27	18	131 hypo	100 UP	141 poor	46 UP	21
							47 hyper	39 DN	86 favorable	73 DN	
							47 hypo/hyper	20 UP/DN	77 insignificant	115 UP/DN	
							225		227	234	
				305	305	305	305	305	305	305	305
				16.72131	8.852459016	5.901639344			1.639534884	111 poor	
										60 good	
										1.85	

Supplementary Table 4: JIGs and their effect on patient survival prognosis in different cancer types of TCGA based on Cox regression analysis

poor prognosis = 1, favorable prognosis = -1, insignificant = 0

Gene_name	Gene_ID	KIRC	LGG	KIRP	PAAD	UVM	ACC	MESO	BLCA	LIHC	STAD	UCEC	LUAD	AML	SKCM	LAML	LUSC	SARC	KICH	HNSC	CESC	BRCA	THYM	OV	THCA	COAD	ESCA	GBM	PCPG	PRAD	WT	READ	DLBC	CHOL	UCS	TGCT	#poor	#favorable	#total
SOX9	ENSG00000125398	-1	1	1	0	0	0	0	0	0	0	0	0	0	0	-1	1	1	1	1	0	0	1	0	0	0	1	1	-1	0	1	1	0	0	-1	-1	11	5	16
CBFB	ENSG00000067955	1	1	0	0	0	0	0	0	0	0	1	-1	1	1	1	1	1	0	1	1	0	1	0	1	0	1	0	0	0	0	0	0	0	1	0	14	1	15
PITX1	ENSG00000069011	1	0	1	0	0	0	0	-1	0	0	-1	1	1	1	0	1	0	1	0	1	0	1	0	0	0	1	1	0	0	0	1	0	0	1	0	13	2	15
ARHGAP29	ENSG00000137962	-1	1	1	0	1	0	0	0	0	0	1	-1	0	0	0	1	0	0	0	1	1	1	0	0	1	0	0	1	0	1	0	1	0	1	0	13	2	15
EGFR	ENSG00000146648	0	0	1	0	1	-1	0	0	-1	-1	1	0	-1	1	-1	1	-1	0	0	0	1	1	0	0	0	0	1	1	0	0	0	0	0	0	0	9	6	15
GAD1	ENSG00000128683	0	0	-1	0	-1	1	0	0	0	0	0	-1	1	1	0	-1	1	0	-1	1	-1	0	0	0	0	0	-1	-1	0	0	0	-1	0	1	0	6	9	15
KLF2	ENSG00000127528	1	0	0	-1	-1	0	0	1	0	0	-1	0	-1	-1	0	-1	-1	0	1	0	0	0	-1	-1	0	-1	0	0	0	0	0	-1	0	1	0	4	11	15
DHRS3	ENSG00000162496	-1	-1	-1	0	0	0	0	0	0	0	0	-1	-1	0	0	1	-1	0	0	0	1	1	0	-1	0	-1	-1	0	0	-1	0	-1	0	1	0	4	11	15
COL11A1	ENSG00000060718	1	1	0	0	1	0	0	0	0	0	0	0	1	1	0	1	1	1	0	1	0	1	1	0	0	1	0	0	0	0	0	1	0	-1	0	13	1	14
DLX2	ENSG00000115844	1	0	0	0	0	0	1	0	0	0	1	1	1	1	0	1	1	1	0	1	0	0	0	0	0	1	0	0	0	0	-1	1	0	1	0	13	1	14
ZIC5	ENSG00000139800	1	1	0	1	-1	0	0	0	0	0	0	1	1	1	0	1	1	1	-1	1	0	0	0	0	0	1	0	0	0	0	0	1	0	0	0	12	2	14
SH3PXD2B	ENSG00000174705	-1	1	1	0	1	0	0	0	-1	0	0	1	0	0	0	1	1	0	0	1	1	1	0	0	0	0	1	1	0	0	0	0	0	1	0	12	2	14
SATB2	ENSG00000119042	0	0	1	0	1	0	0	0	1	0	0	0	-1	-1	0	1	0	1	0	1	0	0	1	0	0	1	1	0	0	1	0	0	0	1	-1	11	3	14
FLVCR1	ENSG00000162769	1	1	-1	0	0	0	0	1	1	-1	0	0	0	1	0	0	1	0	0	1	0	-1	1	0	0	1	1	0	0	0	0	0	0	1	0	11	3	14
CHST11	ENSG00000171310	-1	-1	1	0	0	0	0	0	0	1	1	0	1	0	0	0	1	1	1	1	0	1	0	0	0	0	0	1	0	0	0	0	0	1	-1	11	3	14
SOX11	ENSG00000176887	1	0	1	1	0	0	1	0	0	0	0	0	1	1	0	0	1	0	0	1	0	1	0	0	0	1	1	0	0	0	0	1	0	1	0	13	0	13
COL1A1	ENSG00000108821	1	0	1	0	1	0	0	0	0	0	0	1	1	1	-1	1	0	1	0	1	0	1	0	0	0	0	1	1	0	0	0	0	0	0	0	12	1	13
POSTN	ENSG00000133110	0	1	1	0	1	0	0	0	0	0	0	0	1	1	0	1	1	1	0	1	0	1	0	0	0	0	0	1	0	0	0	1	0	-1	0	12	1	13
ASAP1	ENSG00000153317	0	0	1	0	1	0	0	0	0	0	0	0	0	1	0	1	1	1	1	1	1	1	0	0	0	0	0	0	0	0	0	1	0	1	-1	12	1	13
GJB2	ENSG00000165474	1	1	1	0	1	0	0	-1	0	1	0	0	1	0	0	1	0	1	0	0	0	1	0	0	0	1	1	0	0	1	0	0	0	0	0	12	1	13
OSTM1	ENSG00000081087	-1	0	1	1	0	0	0	0	1	0	1	0	1	1	0	1	1	0	1	1	0	0	0	0	0	-1	0	1	0	0	0	0	0	0	0	11	2	13
MTHFD1L	ENSG00000120254	0	-1	1	0	1	0	0	0	0	0	1	-1	1	1	0	1	1	1	0	0	0	1	-1	0	0	0	0	0	0	0	0	1	0	0	0	10	3	13
FBN2	ENSG00000138829	1	0	1	0	0	0	0	0	-1	0	0	1	1	0	0	0	1	1	-1	1	0	0	0	0	0	1	0	1	0	0	0	1	0	0	-1	10	3	13
CHTOP	ENSG00000160679	1	0	-1	0	0	0	0	-1	1	0	0	0	1	1	0	0	1	0	0	1	-1	0	1	0	0	1	1	0	0	0	0	0	0	0	1	10	3	13
SHOX2	ENSG00000168779	1	1	1	0	0	0	0	0	0	0	-1	0	1	1	0	1	1	1	-1	1	0	0	0	0	0	0	0	0	0	0	0	1	-1	0	0	10	3	13
MIGA2	ENSG00000148343	1	0	-1	0	-1	0	1	0	0	0	-1	0	1	-1	0	0	-1	0	0	0	1	-1	0	0	0	-1	0	-1	0	0	0	0	0	0	1	5	8	13
PTHLH	ENSG00000087494	0	0	1	0	1	0	0	0	0	0	0	1	1	1	1	1	1	0	0	1	0	1	0	0	0	0	0	1	0	0	0	0	0	1	0	12	0	12
FBN1	ENSG00000166147	1	1	1	0	0	0	0	0	0	0	0	1	0	1	0	1	0	0	0	1	0	1	0	0	0	0	0	1	0	1	1	1	0	0	0	12	0	12
TWSG1	ENSG00000128791	1	0	0	0	1	0	0	1	0	1	0	0	0	1	0	1	0	0	0	1	-1	1	1	0	1	0	0	1	0	0	0	0	0	0	0	11	1	12
LTBP1	ENSG00000049323	1	1	1	0	0	0	1	-1	0	0	0	0	1	1	0	1	0	0	0	1	0	1	0	0	0	0	0	1	0	0	0	0	0	-1	0	10	2	12
PHEX	ENSG00000102174	1	0	1	0	0	0	0	0	0	0	1	1	1	1	0	1	1	0	0	0	0	0	0	0	0	0	-1	0	0	0	0	1	0	1	-1	10	2	12
CDC73	ENSG00000134371	1	1	1	0	1	0	0	0	0	-1	0	0	-1	1	0	1	1	1	0	0	0	1	0	0	0	0	0	0	0	0	0	0	0	1	0	10	2	12
MMP14	ENSG00000157227	1	0	1	0	1	0	0	0	0	0	1	0	1	0	-1	1	1	1	0	1	0	1	0	-1	0	0	0	0	0	0	0	0	0	0	0	10	2	12
HAT1	ENSG00000128708	1	0	1	0	0	1	0	-1	0	0	0	0	-1	1	0	1	1	1	0	0	0	1	0	0	0	0	0	-1	0	0	0	1	0	0	0	9	3	12
GJA1	ENSG00000152661	-1	0	1	0	0	0	0	0	0	0	0	0	-1	0	-1	1	0	0	0	1	0	1	0	0	1	0	1	1	0	1	0	0	0	1	0	9	3	12
SFN	ENSG00000175793	1	1	0	0	0	0	0	0	0	0	0	0	1	1	0	1	1	1	0	0	0	1	-1	0	0	0	1	-1	0	0	0	-1	0	0	0	9	3	12
PTK7	ENSG00000112655	1	0	1	0	0	0	0	0	0	0	0	0	1	0	0	1	1	-1	0	1	0	0	0	-1	0	1	1	0	0	0	-1	0	0	-1	0	8	4	12
FAM20C	ENSG00000177706	0	0	1	0	0	0	0	0	0	1	0	-1	0	0	0	1	-1	1	0	1	1	-1	0	-1	0	0	0	1	0	0	0	0	0	1	0	8	4	12
GAS1	ENSG00000180447	0	0	1	0	0	0	0	0	0	-1	0	0	1	1	0	1	0	0	0	-1	1	1	0	-1	1	0	0	1	0	0	0	0	0	-1	0	8	4	12
SPRY4	ENSG00000187678	1	0	0	1	1	0	0	0	0	0	0	-1	-1	1	0	1	0	0	1	-1	0	0	0	0	0	1	0	0	0	0	0	0	0	1	-1	8	4	12
RBBP7	ENSG00000102054	-1	0	0	0	0	0	0	0	1	0	0	1	1	1	1	-1	1	1	-1	0	0	0	0	0	0	0	0	0	0	-1	0	-1	0	0	0	7	5	12
TFAP2A	ENSG00000137203	0	1	0	0	0	0	0	0	-1	0	0	-1	1	1	0	0	1	1	0	1	0	1	0	0	0	0	0	0	0	0	0	0	-1	-1	-1	7	5	12
TNFRSF11B	ENSG00000164761	0	0	0	-1	1	0	0	-1	0	0	-1	0	-1	0	0	1	1	0	0	1	0	1	0	0	0	0	-1	0	0	1	1	0	0	0	0	7	5	12
SMAD2	ENSG00000175387	1	1	1	0	0	0	-1	0	0	0	0	0	-1	-1	0	0	1	0	0	0	0	1	0	1	0	0	0	0	0	1	-1	0	0	-1	0	7	5	12
LYN	ENSG00000254087	-1	0	0	-1	0	0	0	1	0	0	0	0	0	1	1	1	0	0	0	0	0	1	0	0	0	-1	-1	0	0	0	1	0	0	1	-1	7	5	12
SMARCA4	ENSG00000127616	1	0	0	0	0	0	0	0	-1	0	-1	0	1	0	0	0	1	0	0	0	0	-1	0	0	0	1	1	-1	0	-1	-1	-1	0	0	0	5	7	12
TGFBR2	ENSG00000163513	-1	0	0	0	0	0	0	0	0	0	0	0	-1	0	-1	1	-1	-1	1	0	1	1	0	0	0	-1	-1	1	0	0	0	0	0	0	0	5	7	12
IFT27	ENSG00000100360	0	0	-1	0	-1	0	0	0	0	0	0	0	1	-1	0	-1	0	0	-1	0	-1	-1	0	-1	0	1	0	0	0	0	1	-1	0	0	0	3	9	12
IRF6	ENSG00000117595	-1	0	-1	0	1	0	0	0	0	0	0	-1	-1	-1	0	0	-1	0	0	0	-1	1	1	0	0	0	0	0	-1	0	0	0	0	-1	0	3	9	12
WDR19	ENSG00000157796	0	1	0	0	0	0	0	0	0	0	-1	0	1	-1	-1	1	0	-1	0	-1	0	-1	0	0	0	0	-1	0	0	0	0	0	-1	-1	0	3	9	12
WWP2	ENSG00000198373	-1	0	0	0	0	0	0	0	0	0	0	-1	-1	-1	0	-1	0	-1	1	-1	1	0	0	0	0	0	0	0	0	0	-1	-1	0	-1	0	2	10	12
IBSP	ENSG00000029559	1	0	0	0	0	0	1	0	0	0	1	1	1	1	0	1	1	0	0	1	0	1	0	0	0	0	0	0	0	0	0	0	0	1	0	11	0	11
GLI2	ENSG00000074047	0	1	1	0	0	0	0	0	-1	0	0	0	1	1	0	1	0	1	0	0	0	1	0	0	0	0	0	0	0	1	0	1	0	1	0	10	1	11

QSOX1	ENSG00000116260	0	0	0	0	1	0	0	0	0	1	0	0	1	1	0	0	0	0	1	1	1	1	0	-1	0	0	1	0	0	0	0	0	0	0	1	10	1	11
PITX2	ENSG00000164093	1	0	0	0	1	0	0	0	0	0	1	1	1	1	-1	1	1	0	0	0	0	0	0	0	0	1	0	0	0	0	0	0	0	1	0	10	1	11
YBX1	ENSG00000065978	1	-1	0	0	0	0	0	0	0	0	0	0	1	1	0	1	1	0	0	1	-1	1	0	0	0	0	1	0	0	0	0	1	0	0	0	9	2	11
MMP2	ENSG00000087245	0	0	0	0	0	0	0	0	0	1	0	0	1	0	-1	1	0	0	1	1	0	1	0	0	0	0	1	1	0	0	0	0	0	1	-1	9	2	11
RPGRIP1L	ENSG00000103494	0	0	1	1	0	0	0	0	0	-1	0	0	1	0	-1	1	1	0	0	1	0	0	0	0	0	1	0	1	0	1	0	0	0	0	0	9	2	11
MORC2	ENSG00000133422	1	1	0	0	0	0	1	0	0	0	0	0	1	1	0	-1	0	0	-1	1	0	0	0	0	0	0	1	0	0	0	1	0	0	1	0	9	2	11
DVL3	ENSG00000161202	1	0	0	0	0	0	1	0	0	0	0	0	1	1	-1	0	1	1	0	0	0	1	1	0	0	0	1	0	0	0	0	0	0	-1	0	9	2	11
MSX1	ENSG00000163132	1	0	1	0	0	0	0	0	0	0	1	0	1	1	0	1	0	1	0	1	0	0	0	0	0	0	0	0	0	0	-1	-1	0	1	0	9	2	11
AP2B1	ENSG00000006125	0	0	1	0	0	0	-1	1	0	0	1	0	-1	0	0	-1	1	0	1	1	0	1	0	0	0	1	0	0	0	0	0	0	0	0	0	8	3	11
ATP11A	ENSG00000068650	0	0	1	0	1	0	0	0	0	0	1	0	-1	0	0	1	1	-1	1	0	1	1	0	0	0	0	0	0	0	0	0	0	0	-1	0	8	3	11
JAG1	ENSG00000101384	1	1	0	0	0	0	1	0	0	0	0	0	-1	0	0	1	0	1	0	0	0	1	0	-1	0	0	1	0	0	0	0	0	0	1	-1	8	3	11
TRPS1	ENSG00000104447	0	0	1	0	0	0	0	0	-1	0	-1	0	0	0	0	1	0	0	0	0	0	1	0	0	1	1	-1	1	0	1	1	0	0	0	0	8	3	11
OTULIN	ENSG00000154124	0	0	0	0	0	0	0	-1	0	0	-1	1	1	1	0	1	0	0	0	1	0	0	1	0	0	0	-1	0	0	0	0	1	0	1	0	8	3	11
ATR	ENSG00000175054	0	1	0	0	0	0	0	0	0	0	-1	0	1	0	-1	1	1	0	0	0	1	1	0	0	0	1	-1	0	0	1	0	0	0	0	0	8	3	11
FOXD3	ENSG00000187140	1	0	0	0	-1	0	0	0	0	0	0	1	1	1	0	1	0	0	-1	1	0	0	0	0	1	0	0	0	0	0	0	1	0	-1	0	8	3	11
IDS	ENSG00000010404	-1	-1	0	0	0	0	0	1	1	1	1	0	0	1	0	0	0	0	0	0	0	0	0	0	0	-1	-1	1	0	0	1	0	0	0	0	7	4	11
TP63	ENSG00000073282	0	0	-1	-1	0	0	1	0	0	1	0	0	1	1	0	1	0	0	-1	0	0	1	0	0	1	0	0	0	0	0	0	0	0	0	-1	7	4	11
FBLN1	ENSG00000077942	1	0	0	0	0	0	0	0	0	0	0	0	1	1	-1	1	0	0	0	0	1	0	0	-1	0	0	1	1	0	0	0	-1	0	0	-1	7	4	11
PGAP1	ENSG00000197121	0	0	0	0	0	0	0	0	0	0	-1	0	-1	1	0	0	0	0	-1	1	0	-1	0	0	0	1	0	1	0	1	0	1	0	1	0	7	4	11
IDUA	ENSG00000127415	0	0	-1	-1	0	0	1	0	0	0	0	0	1	0	0	1	-1	0	0	0	1	-1	0	0	0	0	-1	0	0	0	1	0	0	1	0	6	5	11
PRICKLE1	ENSG00000139174	0	1	1	0	0	-1	0	0	-1	0	0	0	0	-1	0	-1	-1	0	0	1	0	0	0	0	1	0	0	1	0	0	0	0	0	0	-1	5	6	11
NISCH	ENSG00000010322	0	1	0	-1	0	0	1	0	0	0	-1	0	1	-1	0	-1	0	-1	0	0	0	-1	0	0	0	1	0	0	0	0	0	0	0	-1	0	4	7	11
MAPK1	ENSG00000100030	0	0	1	0	0	0	-1	0	-1	-1	0	0	-1	0	1	-1	0	0	0	0	0	1	-1	0	-1	0	0	0	0	0	0	0	0	1	0	4	7	11
IFT57	ENSG00000114446	-1	0	0	-1	0	0	0	0	0	0	0	0	-1	0	1	1	0	-1	1	0	-1	0	0	0	0	0	-1	0	0	0	0	1	0	-1	0	4	7	11
EDNRB	ENSG00000136160	1	0	0	0	0	0	0	0	0	0	0	-1	-1	-1	0	0	-1	0	1	1	-1	0	0	0	0	-1	0	1	0	0	0	0	0	-1	0	4	7	11
SOSTDC1	ENSG00000171243	0	0	1	0	0	0	0	0	0	0	0	0	-1	-1	0	0	0	-1	-1	0	-1	0	0	0	0	1	0	0	0	1	0	1	0	-1	-1	4	7	11
NFIC	ENSG00000141905	0	-1	1	0	-1	0	0	0	-1	0	-1	0	0	-1	0	1	-1	0	0	0	0	0	-1	0	0	0	0	0	0	0	1	-1	0	0	0	3	8	11
B9D2	ENSG00000123810	-1	0	-1	0	-1	0	0	-1	0	0	-1	0	0	0	0	1	0	0	0	0	0	-1	-1	0	0	-1	0	0	0	0	0	-1	0	1	0	2	9	11
KDF1	ENSG00000175707	-1	0	-1	0	0	0	-1	0	0	1	0	-1	-1	-1	0	0	0	0	0	0	0	0	0	0	-1	0	0	-1	-1	0	0	0	0	-1	0	1	10	11
SH3PXD2A	ENSG00000107957	1	0	1	0	0	1	0	0	0	0	0	0	1	1	0	0	0	0	0	1	1	0	0	0	0	0	1	0	0	1	0	0	0	1	0	10	0	10
ZMPSTE24	ENSG00000084073	0	1	0	1	0	0	-1	0	1	0	0	0	-1	1	0	1	1	0	0	0	0	0	0	0	0	0	0	0	0	0	0	1	0	1	0	8	2	10
DNAH11	ENSG00000105877	0	0	1	1	0	0	1	0	0	0	0	1	0	1	-1	1	0	0	0	1	0	0	0	0	0	0	0	0	0	0	1	0	-1	0	0	8	2	10
SIX1	ENSG00000126778	1	0	0	0	0	1	0	0	0	0	0	0	1	1	0	0	1	-1	-1	0	0	1	0	0	0	1	0	0	0	0	0	1	0	0	0	8	2	10
HAPLN1	ENSG00000145681	1	0	0	1	0	0	0	0	0	0	0	1	0	1	0	-1	1	0	-1	1	0	0	0	0	0	1	0	0	0	0	0	0	0	1	0	8	2	10
EDNRA	ENSG00000151617	0	1	1	0	0	0	0	0	0	-1	0	0	0	1	0	0	0	0	0	1	0	1	0	0	1	0	0	1	0	0	0	0	0	1	-1	8	2	10
OTX2	ENSG00000165588	0	0	0	0	0	1	0	0	-1	0	0	0	1	1	0	1	1	0	-1	0	0	0	0	0	0	1	0	1	0	0	0	1	0	0	0	8	2	10
FIGN	ENSG00000182263	0	1	0	0	0	0	0	0	0	0	1	0	1	0	0	0	1	1	1	0	-1	0	-1	0	0	0	0	1	0	0	0	1	0	0	0	8	2	10
PRRX1	ENSG00000116132	0	0	1	0	0	0	0	0	0	-1	-1	1	1	1	-1	0	0	0	0	0	0	1	0	0	0	0	0	1	0	0	0	0	0	1	0	7	3	10
VPS25	ENSG00000131475	0	0	1	0	0	1	0	0	0	0	1	0	1	-1	1	0	0	1	0	0	0	-1	0	0	0	0	1	0	0	-1	0	0	0	0	0	7	3	10
MN1	ENSG00000169184	-1	-1	1	0	0	0	0	0	0	0	1	0	0	1	0	-1	0	1	0	0	0	1	0	0	1	0	0	1	0	0	0	0	0	0	0	7	3	10
PLXND1	ENSG00000004399	0	-1	0	0	0	0	1	0	0	0	0	0	0	1	0	1	0	-1	1	1	0	0	0	0	0	0	0	0	1	0	-1	0	0	-1	0	6	4	10
CYB561	ENSG00000008283	-1	0	0	1	0	0	0	0	0	1	0	0	1	0	0	1	1	-1	1	0	0	0	0	0	0	0	0	-1	0	0	0	-1	0	0	0	6	4	10
CCND1	ENSG00000110092	0	0	0	0	0	1	0	0	0	0	1	0	-1	0	0	0	-1	0	0	1	0	1	0	0	0	0	1	-1	0	0	0	0	0	1	-1	6	4	10
SLC26A2	ENSG00000155850	-1	1	0	0	0	0	0	0	0	0	0	1	0	0	-1	1	1	0	0	0	0	0	0	0	0	0	-1	0	0	0	0	-1	1	1	0	6	4	10
PDS5B	ENSG00000083642	1	1	1	0	0	0	0	0	0	0	0	0	-1	0	-1	0	0	0	0	0	1	0	0	-1	0	0	-1	0	0	0	-1	0	0	1	0	5	5	10
TP53	ENSG00000141510	0	0	0	0	-1	0	-1	0	0	-1	0	0	1	0	0	1	-1	0	0	0	0	0	0	0	0	0	1	0	0	1	0	-1	1	0	0	5	5	10
ZEB1	ENSG00000148516	0	0	1	0	0	0	0	0	0	-1	0	0	-1	1	0	-1	0	0	0	0	0	0	0	0	1	0	-1	1	0	0	-1	0	0	1	0	5	5	10
PTH1R	ENSG00000160801	0	0	1	0	0	0	1	0	0	0	0	0	-1	-1	-1	-1	0	-1	1	0	0	0	0	0	1	0	0	1	0	0	0	0	0	0	0	5	5	10
MFSD12	ENSG00000161091	1	-1	0	0	0	0	0	0	0	0	0	0	0	-1	0	1	0	1	0	1	1	-1	0	0	0	0	0	-1	0	0	0	-1	0	0	0	5	5	10
DYNC2H1	ENSG00000187240	0	1	1	0	0	0	0	0	-1	0	0	0	-1	0	-1	1	0	0	0	-1	0	0	0	0	0	0	0	1	0	1	0	0	0	-1	0	5	5	10
SH3BP2	ENSG00000087266	-1	0	-1	0	0	0	0	0	0	1	-1	0	0	0	0	1	-1	0	0	0	0	0	0	0	0	0	0	-1	0	0	0	0	-1	1	1	4	6	10
BCL11B	ENSG00000127152	0	0	0	-1	0	0	0	0	0	1	0	0	1	1	-1	0	-1	-1	0	0	0	0	0	0	0	0	-1	-1	0	0	0	0	0	1	0	4	6	10
HRAS	ENSG00000174775	1	-1	0	0	0	0	0	0	0	0	0	0	1	-1	0	-1	0	1	0	1	0	-1	0	-1	0	0	0	0	0	-1	0	0	0	0	0	4	6	10
NFATC1	ENSG00000131196	0	0	1	-1	-1	-1	1	0	0	0	-1	0	0	-1	0	1	0	0	0	0	0	0	0	-1	0	0	0	0	0	0	0	0	0	-1	0	3	7	10
FREM1	ENSG00000164946	0	1	0	-1	-1	0	0	0	0	0	0	-1	-1	0	-1	1	0	-1	0	0	0	0	0	0	0	0	0	1	0	0	0	0	0	0	-1	3	7	10
SLC25A21	ENSG00000183032	-1	0	0	0	0	0	0	0	0	0	0	0	-1	-1	-1	-1	1	0	-1	0	0	0	-1	0	0	0	0	1	0	0	0	0	0	-1	0	2	8	10
PDGFRB	ENSG00000113721	0	0	1	0	0	0	0	0	0	0	0	0	0	1	0	1	0	0	1	1	0	1	0	0	0	0	1	1	0	0	0	0	0	1	0	9	0	9
ITGB1	ENSG00000150093	0	0	1	0	1	0	0	0	0	0	0	0	0	1	0	1	0	1	1	1	0	1	0	0	0	0	0	1	0	0	0	0	0	0	0	9	0	9
TMEM67	ENSG00000164953	0	0	0	1	0	0	0	0	0	0	0	0	1	1	0	1	1	0	0	0	0	0	0	1	0	1	0	0	0	1	0	0	0	1	0	9	0	9
TGFB2	ENSG00000092969	1	0	1	0	0	0	0	0	0	0	0	0	-1	1	0	1	0	0	0	1	0	1	0	0	1	0	0	1	0	0	0	0	0	0	0	8	1	9

HOXA3	ENSG00000105997	0	1	0	0	1	0	0	0	0	0	0	0	1	0	1	1	0	1	0	0	1	0	-1	0	0	0	0	0	0	0	0	0	0	0	0	0	1	8	1	9
POLE4	ENSG00000115350	1	0	0	0	0	1	0	0	0	1	1	1	0	0	1	0	0	1	0	0	0	0	0	0	0	0	1	0	0	-1	0	0	0	0	0	8	1	9
NDST1	ENSG00000070614	0	-1	0	0	1	0	0	1	0	0	0	0	-1	0	0	0	0	0	1	1	0	0	0	0	0	0	1	1	0	0	0	1	0	0	0	7	2	9
HOXA2	ENSG00000105996	0	1	0	0	0	0	0	0	0	0	0	0	1	0	0	1	-1	0	0	0	1	0	-1	0	1	1	0	0	0	0	0	0	0	0	1	7	2	9
RSPO2	ENSG00000147655	0	1	1	0	0	0	0	0	0	0	0	1	1	0	0	0	0	-1	0	1	0	0	0	0	0	0	-1	0	0	0	1	1	0	0	0	7	2	9
DRC1	ENSG00000157856	1	0	1	-1	0	0	0	0	0	1	0	0	1	0	0	1	1	0	0	1	0	-1	0	0	0	0	0	0	0	0	0	0	0	0	0	7	2	9
ASXL1	ENSG00000171456	0	0	0	0	0	0	0	0	0	0	-1	0	1	0	0	1	0	0	0	0	0	0	1	1	0	1	0	0	0	1	-1	1	0	0	0	7	2	9
CDON	ENSG00000064309	0	0	1	-1	0	0	0	0	0	0	0	1	0	1	0	-1	1	0	-1	0	0	0	1	0	0	0	0	0	0	0	0	1	0	0	0	6	3	9
GLG1	ENSG00000090863	0	0	1	1	1	0	0	0	0	0	1	0	-1	-1	0	1	0	1	0	0	0	0	0	0	0	0	0	0	0	0	0	0	-1	0	0	6	3	9
DLX5	ENSG00000105880	0	0	0	0	0	0	1	0	0	0	0	0	1	1	-1	0	1	0	-1	1	0	0	0	0	0	0	0	0	0	0	0	-1	0	1	0	6	3	9
ARSB	ENSG00000113273	0	0	1	0	0	0	-1	0	0	1	0	1	-1	0	0	0	0	0	0	1	0	0	1	0	0	-1	0	1	0	0	0	0	0	0	0	6	3	9
FOXF2	ENSG00000137273	0	0	0	0	0	0	0	0	0	-1	1	-1	0	1	0	-1	1	0	0	1	0	1	0	0	0	0	0	0	0	0	0	0	0	1	0	6	3	9
COL2A1	ENSG00000139219	1	1	0	0	0	0	0	0	0	0	0	0	0	1	-1	0	0	0	0	1	0	-1	0	0	0	1	0	1	0	0	0	0	0	-1	0	6	3	9
PRDM16	ENSG00000142611	0	1	0	0	0	0	0	0	0	0	0	-1	-1	1	1	1	0	-1	1	0	0	0	0	0	0	0	0	0	0	0	0	0	0	1	0	6	3	9
HSPG2	ENSG00000142798	0	0	1	0	1	0	0	0	0	0	0	0	-1	1	0	1	0	0	0	1	0	0	0	0	0	-1	0	0	0	1	0	0	0	-1	0	6	3	9
PSPH	ENSG00000146733	0	0	1	0	0	0	0	0	0	-1	1	0	0	0	0	1	1	1	-1	0	0	-1	0	0	0	0	0	0	0	0	0	0	0	1	0	6	3	9
NOG	ENSG00000183691	0	-1	0	0	0	0	0	0	0	0	1	0	1	0	-1	-1	0	0	1	0	0	1	0	0	0	1	0	0	0	0	0	0	0	1	0	6	3	9
TBX1	ENSG00000184058	1	0	-1	0	0	0	0	-1	0	0	0	0	0	1	-1	1	0	0	0	0	0	0	0	0	0	1	0	0	0	0	0	0	1	1	0	6	3	9
LRP2	ENSG00000081479	1	0	0	-1	0	0	1	0	0	0	0	0	-1	-1	0	0	1	0	1	-1	0	0	0	0	0	0	0	0	0	0	0	1	0	0	0	5	4	9
PAX1	ENSG00000125813	0	0	1	0	0	0	0	0	0	0	0	0	1	1	0	-1	0	1	0	0	0	-1	0	0	0	0	0	0	0	0	-1	1	-1	0	0	5	4	9
KDR	ENSG00000128052	-1	0	0	0	0	0	0	0	0	0	0	0	-1	1	-1	0	-1	0	0	0	0	0	0	0	1	0	1	1	0	0	0	0	0	1	0	5	4	9
MAP3K7	ENSG00000135341	-1	0	1	0	0	0	0	0	0	0	0	0	1	0	0	0	1	1	0	0	-1	0	0	0	0	1	-1	0	0	0	-1	0	0	0	0	5	4	9
CRIM1	ENSG00000150938	-1	0	0	0	0	0	0	0	0	0	1	0	-1	0	-1	1	0	0	0	-1	0	1	0	0	0	0	0	1	0	0	0	1	0	0	0	5	4	9
SCHIP1	ENSG00000151967	0	0	0	0	0	0	1	0	0	0	0	0	0	-1	1	0	0	0	0	1	0	-1	0	0	0	0	-1	1	0	0	-1	1	0	0	0	5	4	9
TIPARP	ENSG00000163659	0	0	0	-1	0	0	0	0	0	0	0	0	-1	0	0	1	0	0	1	0	1	1	0	0	0	0	-1	0	0	0	1	0	0	-1	0	5	4	9
LIMK2	ENSG00000182541	0	0	0	0	0	0	0	0	-1	0	0	0	1	-1	1	1	0	0	-1	1	0	0	0	0	0	-1	0	0	0	1	0	0	0	0	0	5	4	9
EPS8L3	ENSG00000198758	0	0	-1	-1	0	0	0	0	0	0	0	0	1	1	0	0	1	1	0	0	0	1	0	0	0	0	0	-1	0	0	0	0	0	0	-1	5	4	9
FGF8	ENSG00000107831	1	0	0	0	-1	0	0	0	0	0	-1	0	1	1	0	-1	0	0	-1	0	0	0	0	0	0	0	0	-1	0	0	0	0	1	0	0	4	5	9
EGR2	ENSG00000122877	0	0	1	-1	0	0	0	0	0	0	0	-1	0	0	0	1	0	-1	0	0	0	0	0	-1	1	0	-1	0	0	0	0	0	0	1	0	4	5	9
SMO	ENSG00000128602	1	0	0	0	0	0	0	0	0	0	0	-1	1	-1	-1	1	0	0	0	0	0	0	0	-1	0	0	0	1	0	0	-1	0	0	0	0	4	5	9
DISP1	ENSG00000154309	0	0	0	0	0	0	0	1	0	0	0	0	-1	0	0	1	0	0	1	-1	0	-1	0	0	0	0	0	1	0	-1	0	0	0	-1	0	4	5	9
MKS1	ENSG00000011143	0	0	0	0	0	0	0	0	-1	0	0	0	1	-1	0	0	0	0	0	0	0	-1	0	0	-1	1	1	-1	0	0	0	0	0	-1	0	3	6	9
SPRY2	ENSG00000136158	1	0	0	-1	0	1	0	0	0	0	0	-1	-1	0	0	1	0	0	0	-1	0	0	0	0	0	0	0	0	0	0	0	0	0	-1	-1	3	6	9
MECP2	ENSG00000169057	-1	0	0	0	-1	0	1	0	0	0	0	0	-1	0	0	0	0	-1	0	-1	0	-1	0	0	1	0	0	0	0	0	0	1	0	0	0	3	6	9
IFT140	ENSG00000187535	-1	0	0	0	0	0	0	0	0	0	-1	-1	0	0	0	1	0	0	0	-1	0	0	0	0	-1	1	0	0	0	1	0	-1	0	0	0	3	6	9
FGFR2	ENSG00000066468	0	0	0	0	-1	0	0	0	-1	0	-1	0	-1	0	-1	0	0	-1	-1	0	0	0	0	0	0	0	0	0	0	0	0	-1	0	0	1	1	8	9
TTC28	ENSG00000100154	0	0	0	0	0	0	0	0	-1	0	-1	0	-1	0	0	0	-1	-1	-1	0	-1	0	0	0	0	0	0	1	0	0	0	-1	0	0	0	1	8	9
DPH1	ENSG00000108963	-1	0	0	0	0	0	0	0	0	0	0	-1	1	-1	0	0	-1	-1	0	-1	0	-1	0	0	0	0	0	0	0	0	0	0	0	-1	0	1	8	9
MBTPS1	ENSG00000140943	0	0	0	0	0	0	0	0	0	0	0	-1	-1	0	-1	1	-1	0	0	0	0	0	-1	0	0	0	0	0	0	0	0	-1	-1	-1	0	1	8	9
KAT14	ENSG00000149474	-1	0	0	0	0	0	0	0	0	0	0	0	-1	-1	0	-1	0	0	0	0	0	-1	0	-1	-1	0	0	0	0	0	0	-1	0	-1	0	0	9	9
FOXC2	ENSG00000176692	1	0	1	0	1	0	0	0	0	0	1	0	0	1	0	0	0	0	0	1	0	0	0	0	0	0	0	1	0	0	0	0	0	1	0	8	0	8
TCOF1	ENSG00000070814	0	0	0	0	0	0	0	-1	0	0	0	1	1	1	0	0	1	1	0	1	0	0	0	0	0	0	0	0	0	0	0	1	0	0	0	7	1	8
TP53BP2	ENSG00000143514	0	0	0	0	1	0	0	0	0	0	0	0	0	1	-1	0	0	0	0	1	0	1	0	0	1	1	0	0	0	0	0	1	0	0	0	7	1	8
ROR2	ENSG00000169071	0	0	1	0	0	0	0	0	0	1	0	0	1	1	-1	1	0	0	0	0	0	0	0	0	0	0	0	1	0	1	0	0	0	0	0	7	1	8
BNC2	ENSG00000173068	0	0	1	0	0	0	0	0	0	0	0	0	0	0	-1	1	0	0	0	0	0	1	0	0	1	0	0	1	0	0	0	1	0	1	0	7	1	8
AMBN	ENSG00000178522	0	0	0	0	0	0	0	0	0	1	0	0	1	0	1	0	1	0	0	0	0	0	1	1	0	1	0	0	0	0	0	0	0	-1	0	7	1	8
FGFR1	ENSG00000077782	0	-1	1	0	0	0	0	0	0	1	0	0	0	1	0	1	0	0	0	0	0	0	0	-1	0	0	0	1	0	0	0	0	0	1	0	6	2	8
LOXL3	ENSG00000115318	-1	0	0	0	-1	0	0	0	0	0	0	0	1	0	0	1	0	0	0	0	0	1	0	0	0	0	0	1	0	0	1	0	0	1	0	6	2	8
ECE1	ENSG00000117298	0	1	0	0	0	0	0	0	-1	0	0	0	0	0	0	1	0	0	0	1	1	0	-1	0	0	0	0	0	0	0	0	0	1	1	0	6	2	8
BRD4	ENSG00000141867	0	0	0	-1	0	0	0	0	0	0	0	0	1	1	0	1	0	0	0	1	0	0	1	0	0	0	1	0	0	0	-1	0	0	0	0	6	2	8
CASK	ENSG00000147044	0	0	0	1	0	0	0	0	1	0	0	0	0	1	0	-1	0	0	0	0	1	1	0	0	0	0	0	0	-1	0	0	1	0	0	0	6	2	8
GPC3	ENSG00000147257	1	0	0	0	0	0	0	0	0	0	0	0	1	1	1	0	0	-1	-1	0	0	0	0	0	0	0	1	1	0	0	0	0	0	0	0	6	2	8
PKDCC	ENSG00000162878	-1	1	1	0	0	0	1	0	0	0	0	0	1	0	0	0	0	0	0	1	0	0	0	0	0	0	0	0	0	1	0	-1	0	0	0	6	2	8
ROBO1	ENSG00000169855	1	0	1	0	0	0	0	0	0	0	-1	0	0	0	0	0	1	0	0	1	0	0	0	0	0	1	0	0	0	1	0	0	0	-1	0	6	2	8
FOXC1	ENSG00000054598	0	0	0	0	-1	0	1	0	0	-1	0	0	0	0	0	1	0	0	0	-1	1	1	0	0	0	0	0	0	0	0	0	0	0	1	0	5	3	8
ELP1	ENSG00000070061	0	1	1	1	0	0	0	0	0	0	0	0	-1	0	0	0	0	0	0	0	1	-1	0	1	0	0	0	0	0	0	0	0	0	-1	0	5	3	8
EYA1	ENSG00000104313	0	-1	1	0	0	0	0	0	0	0	0	0	1	0	-1	-1	1	0	0	0	0	0	0	0	0	0	0	1	0	1	0	0	0	0	0	5	3	8
KIF3A	ENSG00000131437	0	1	0	0	0	0	0	0	1	0	0	0	-1	0	0	-1	1	0	0	0	0	-1	0	0	1	1	0	0	0	0	0	0	0	0	0	5	3	8
NABP2	ENSG00000139579	0	0	0	0	-1	0	0	0	0	0	0	1	1	0	1	0	1	1	0	0	-1	0	0	0	0	0	0	0	0	-1	0	0	0	0	0	5	3	8
DAAM2	ENSG00000146122	0	1	1	0	1	0	0	0	0	0	0	0	0	1	0	-1	-1	-1	0	0	0	0	0	0	0	0	0	1	0	0	0	0	0	0	0	5	3	8

DOCK1	ENSG00000150760	0	1	1	0	1	0	0	0	0	0	0	0	-1	0	0	0	0	-1	0	0	0	1	0	0	0	0	0	0	0	0	0	0	0	1	-1	5	3	8
CHD1	ENSG00000153922	0	0	0	0	0	0	0	0	1	0	0	0	0	1	-1	1	0	0	0	0	0	1	0	0	0	0	-1	0	0	1	-1	0	0	0	0	5	3	8
EDN1	ENSG00000078401	0	1	0	-1	0	0	0	1	0	0	0	-1	-1	0	0	0	0	0	0	0	0	1	0	0	-1	0	0	0	0	0	1	0	0	0	0	4	4	8
PAK1IP1	ENSG00000111845	0	0	0	0	0	1	0	0	0	0	0	0	0	1	0	-1	1	0	-1	0	-1	0	0	0	0	1	0	0	0	0	-1	0	0	0	0	4	4	8
TENT5A	ENSG00000112773	-1	0	0	0	0	0	0	0	1	0	0	0	0	0	-1	1	0	-1	1	0	0	-1	0	0	0	0	0	1	0	0	0	0	0	0	0	4	4	8
ALDH1A2	ENSG00000128918	0	0	-1	-1	0	0	0	0	0	0	0	0	0	0	0	0	-1	0	1	-1	1	0	0	0	0	0	0	1	0	0	0	0	0	1	0	4	4	8
APC	ENSG00000134982	-1	1	1	1	0	0	0	0	0	0	-1	0	-1	0	0	0	0	0	0	0	1	0	0	0	0	0	-1	0	0	0	0	0	0	0	0	4	4	8
ZIC3	ENSG00000156925	0	0	0	1	0	0	0	0	0	-1	0	0	0	1	0	-1	0	-1	0	1	0	0	0	0	0	0	-1	0	0	0	0	1	0	0	0	4	4	8
SHH	ENSG00000164690	0	0	0	0	0	0	0	1	0	0	0	-1	0	0	-1	-1	-1	0	0	1	0	0	0	0	0	1	0	0	0	1	0	0	0	0	0	4	4	8
CPLANE1	ENSG00000197603	0	1	1	0	0	0	0	0	-1	0	0	1	-1	0	-1	0	0	0	0	0	0	0	0	0	0	0	0	0	0	1	-1	0	0	0	0	4	4	8
PAX9	ENSG00000198807	0	0	0	0	-1	1	0	0	0	0	0	0	1	0	0	-1	1	0	-1	1	0	0	-1	0	0	0	0	0	0	0	0	0	0	0	0	4	4	8
ABHD5	ENSG00000011198	-1	0	-1	0	0	0	-1	0	0	0	0	1	-1	0	0	0	0	0	0	0	0	1	1	0	0	0	0	0	0	0	0	0	0	-1	0	3	5	8
ALKBH1	ENSG00000100601	0	0	0	0	0	0	0	0	0	0	1	0	-1	0	0	-1	0	0	-1	0	0	-1	1	0	0	0	0	0	0	0	-1	1	0	0	0	3	5	8
BMP5	ENSG00000112175	0	0	0	-1	0	0	-1	0	0	0	0	0	0	0	-1	1	0	-1	1	0	0	0	0	0	-1	1	0	0	0	0	0	0	0	0	0	3	5	8
SLC10A7	ENSG00000120519	-1	0	0	1	0	0	-1	0	0	0	0	0	-1	0	-1	1	0	0	0	0	0	1	0	0	0	0	0	-1	0	0	0	0	0	0	0	3	5	8
CPLANE2	ENSG00000132881	-1	0	1	0	0	0	0	0	0	1	0	0	-1	-1	0	0	0	0	1	-1	0	0	0	-1	0	0	0	0	0	0	0	0	0	0	0	3	5	8
PCSK6	ENSG00000140479	1	0	0	-1	0	0	0	0	0	0	0	0	-1	1	0	-1	-1	0	0	0	0	1	0	0	0	0	0	0	0	0	0	0	0	-1	0	3	5	8
LRRK1	ENSG00000154237	0	-1	0	0	0	0	0	-1	0	0	0	0	1	0	0	1	0	0	0	0	-1	1	0	0	0	0	0	0	0	0	0	0	0	-1	-1	3	5	8
SLC38A10	ENSG00000157637	0	0	0	0	0	0	0	0	1	0	0	0	1	1	0	0	0	0	0	-1	0	-1	0	-1	0	-1	0	0	0	0	0	-1	0	0	0	3	5	8
PRKRA	ENSG00000180228	1	-1	0	0	0	0	0	0	0	0	0	0	0	0	0	0	1	0	0	0	-1	0	-1	0	0	0	0	0	-1	-1	0	1	0	0	0	3	5	8
CHUK	ENSG00000213341	1	0	1	1	0	0	-1	0	0	0	0	0	-1	0	0	-1	0	0	0	0	0	0	0	0	0	-1	0	0	0	-1	0	0	0	0	0	3	5	8
BMP4	ENSG00000125378	1	0	0	0	0	0	0	0	0	0	0	0	-1	0	-1	-1	0	0	0	-1	0	1	0	0	0	0	0	-1	0	0	0	0	0	0	-1	2	6	8
ATMIN	ENSG00000166454	-1	0	0	0	0	0	0	1	0	0	0	-1	-1	0	0	0	-1	0	1	-1	0	0	0	0	0	0	0	0	0	0	0	0	-1	0	0	2	6	8
POU3F3	ENSG00000198914	0	0	0	-1	0	0	0	0	0	0	0	-1	-1	-1	0	0	0	0	0	0	0	-1	0	0	0	0	0	0	0	0	0	1	0	1	-1	2	6	8
PTCH1	ENSG00000185920	0	1	0	0	0	0	0	0	0	-1	0	-1	-1	0	-1	-1	0	-1	0	0	0	0	0	0	0	0	0	0	0	0	0	0	0	-1	0	1	7	8
ATPAF2	ENSG00000171953	-1	-1	0	-1	-1	0	0	0	0	0	0	0	0	-1	0	0	0	0	0	-1	0	-1	0	0	0	0	0	0	0	0	0	-1	0	0	0	0	8	8
TBX15	ENSG00000092607	0	0	0	0	0	0	0	0	0	0	0	0	1	1	0	1	0	0	0	1	0	1	0	0	0	0	0	1	0	0	0	1	0	0	0	7	0	7
HAND1	ENSG00000113196	0	0	0	1	1	0	0	1	1	0	0	0	0	1	0	0	0	0	1	0	0	0	0	0	0	0	0	1	0	0	0	0	0	0	0	7	0	7
HAND2	ENSG00000164107	1	0	1	0	0	0	0	0	0	0	0	0	0	1	0	1	0	0	0	0	0	1	0	0	1	0	0	1	0	0	0	0	0	0	0	7	0	7
FGF10	ENSG00000070193	0	0	1	0	0	0	0	0	0	0	0	0	1	1	0	0	0	-1	0	1	0	1	0	0	0	0	0	1	0	0	0	0	0	0	0	6	1	7
DKK1	ENSG00000107984	1	0	0	0	0	0	0	0	0	0	1	0	0	0	0	0	0	1	0	1	0	1	0	0	0	0	0	1	0	0	0	0	0	0	-1	6	1	7
PAX3	ENSG00000135903	0	0	0	0	0	0	0	0	-1	0	0	0	1	1	0	1	1	0	0	0	0	0	0	0	0	0	0	0	0	1	0	1	0	0	0	6	1	7
SLC27A4	ENSG00000167114	1	0	0	0	0	0	0	0	0	0	0	0	0	-1	1	0	0	1	1	1	0	0	0	0	0	0	1	0	0	0	0	0	0	0	0	6	1	7
GLDC	ENSG00000178445	0	1	-1	0	0	0	0	0	0	0	1	0	0	0	0	0	0	0	0	1	0	0	0	0	0	1	0	0	0	0	1	1	0	0	0	6	1	7
ALX1	ENSG00000180318	0	0	0	-1	0	0	0	0	0	0	0	0	1	1	0	0	1	1	0	0	0	0	0	0	0	0	1	0	0	0	0	1	0	0	0	6	1	7
HSPB11	ENSG00000081870	1	0	0	0	-1	0	0	0	0	0	0	1	1	0	0	1	1	0	-1	0	0	0	0	0	0	0	0	0	0	0	0	0	0	0	0	5	2	7
TGDS	ENSG00000088451	0	1	0	1	0	1	0	0	0	0	1	0	0	0	0	0	0	0	0	-1	0	0	0	1	0	0	0	-1	0	0	0	0	0	0	0	5	2	7
CHRD	ENSG00000090539	0	0	0	0	0	1	0	0	0	0	0	-1	1	1	0	0	0	0	0	0	0	0	0	0	0	-1	0	1	0	0	0	0	0	1	0	5	2	7
GLI3	ENSG00000106571	1	0	0	0	0	0	0	0	0	0	0	0	1	0	-1	1	0	0	0	0	0	0	0	-1	0	0	0	1	0	0	0	0	0	1	0	5	2	7
VEGFA	ENSG00000112715	0	0	-1	0	1	0	0	0	0	0	0	0	0	1	0	1	1	0	0	0	-1	0	0	0	0	1	0	0	0	0	0	0	0	0	0	5	2	7
WNT5A	ENSG00000114251	1	0	0	0	0	0	0	0	0	0	0	0	1	0	0	1	0	0	-1	0	0	1	0	0	0	1	0	0	0	0	0	0	-1	0	0	5	2	7
TNFSF11	ENSG00000120659	0	1	0	0	0	0	0	0	0	0	0	0	1	0	0	0	0	1	0	0	0	1	0	0	0	0	-1	0	0	0	0	-1	0	1	0	5	2	7
MYH10	ENSG00000133026	1	0	1	0	0	0	0	0	-1	0	1	0	-1	0	0	0	0	0	0	1	0	0	0	0	0	0	1	0	0	0	0	0	0	0	0	5	2	7
RYK	ENSG00000163785	0	0	0	0	0	0	0	0	0	0	0	0	0	0	-1	1	1	-1	0	0	0	1	0	0	0	1	0	0	0	0	0	1	0	0	0	5	2	7
KMT2D	ENSG00000167548	0	0	0	0	1	0	1	0	0	0	-1	0	0	0	0	1	0	0	0	0	0	0	1	0	0	0	0	0	0	1	-1	0	0	0	0	5	2	7
SETD5	ENSG00000168137	0	0	0	0	0	0	0	0	0	0	0	0	1	0	-1	0	0	0	0	1	0	0	1	0	0	1	0	0	0	1	0	0	0	-1	0	5	2	7
GKN1	ENSG00000169605	1	0	-1	0	0	0	0	0	0	1	0	0	0	0	0	0	0	0	0	1	0	1	0	0	0	-1	1	0	0	0	0	0	0	0	0	5	2	7
GRB2	ENSG00000177885	0	0	1	0	0	0	0	0	0	1	0	0	1	0	0	0	1	0	0	0	0	-1	0	1	0	-1	0	0	0	0	0	0	0	0	0	5	2	7
PKD1	ENSG00000008710	0	0	0	0	0	0	1	0	0	0	-1	0	1	0	0	0	0	0	0	0	1	-1	0	0	0	0	0	0	0	0	0	0	1	-1	0	4	3	7
BMP7	ENSG00000101144	1	0	0	0	0	0	0	0	0	0	0	0	1	0	1	-1	0	0	-1	0	0	0	-1	0	0	0	0	0	0	0	0	1	0	0	0	4	3	7
MEGF8	ENSG00000105429	0	0	0	0	1	0	0	0	0	0	0	0	0	-1	0	1	0	1	0	0	0	-1	0	-1	0	0	1	0	0	0	0	0	0	0	0	4	3	7
IDE	ENSG00000119912	0	1	0	0	0	0	-1	0	0	0	0	0	0	0	0	0	0	0	0	0	0	1	1	0	0	0	0	-1	0	0	0	-1	0	1	0	4	3	7
SMG1	ENSG00000157106	0	1	0	0	0	0	0	0	0	0	0	0	0	0	-1	1	0	0	0	0	0	1	0	0	0	0	-1	0	0	1	-1	0	0	0	0	4	3	7
JAG2	ENSG00000184916	0	1	0	0	0	0	1	0	-1	0	0	0	-1	0	0	0	0	0	0	0	0	-1	0	0	0	0	1	0	0	0	0	0	0	1	0	4	3	7
GAS2L2	ENSG00000270765	1	0	0	0	0	0	1	0	0	0	0	0	1	0	-1	1	0	0	0	0	0	0	0	0	0	0	0	0	0	0	-1	0	0	-1	0	4	3	7
PIGV	ENSG00000060642	0	0	1	0	0	0	0	0	0	1	0	0	-1	0	0	1	0	0	0	-1	0	0	0	-1	0	0	0	0	0	0	0	-1	0	0	0	3	4	7
DLG1	ENSG00000075711	0	0	0	1	0	0	0	0	0	0	0	1	0	-1	-1	0	0	0	-1	0	0	1	0	0	0	0	-1	0	0	0	0	0	0	0	0	3	4	7
MEF2C	ENSG00000081189	-1	0	0	0	0	0	0	0	0	0	0	0	-1	0	0	0	-1	-1	1	0	0	0	0	0	1	0	0	1	0	0	0	0	0	0	0	3	4	7
CRKL	ENSG00000099942	1	0	0	0	0	0	0	0	-1	0	0	0	0	1	0	-1	0	0	0	0	0	0	0	0	0	0	1	0	0	0	-1	0	0	-1	0	3	4	7
WDR11	ENSG00000120008	0	1	0	0	1	0	0	0	0	0	0	0	-1	-1	0	0	0	0	0	-1	0	0	0	1	0	0	-1	0	0	0	0	0	0	0	0	3	4	7

KCNJ2	ENSG00000123700	-1	1	0	0	0	1	0	0	0	0	0	0	0	0	0	1	0	0	0	0	0	-1	0	0	-1	0	-1	0	0	0	0	0	0	0	0	3	4	7
FGF9	ENSG00000102678	0	0	0	-1	0	0	0	0	0	0	0	-1	0	0	-1	-1	1	0	0	-1	0	0	0	0	0	0	0	0	0	0	0	0	0	1	0	2	5	7
SRF	ENSG00000112658	0	-1	0	-1	0	0	1	0	0	0	0	0	0	0	0	0	0	0	0	0	0	-1	0	0	0	0	0	0	0	1	-1	0	0	-1	0	2	5	7
PSIP1	ENSG00000164985	0	0	0	0	-1	0	0	0	0	0	0	1	-1	0	0	-1	0	0	0	0	0	-1	0	0	0	0	0	0	0	0	-1	1	0	0	0	2	5	7
DNASE1L2	ENSG00000167968	0	0	-1	0	-1	0	0	0	0	0	0	0	1	0	0	-1	0	0	0	1	0	-1	0	0	0	0	0	-1	0	0	0	0	0	0	0	2	5	7
CDK10	ENSG00000185324	0	0	-1	0	0	0	1	0	0	0	0	-1	1	-1	0	0	-1	0	0	0	0	-1	0	0	0	0	0	0	0	0	0	0	0	0	0	2	5	7
FUZ	ENSG00000010361	-1	-1	0	-1	0	0	0	0	0	0	-1	0	0	-1	0	0	0	0	0	0	0	-1	0	0	0	0	0	1	0	0	0	0	0	0	0	1	6	7
BRAF	ENSG00000157764	-1	0	0	0	0	0	0	0	0	0	-1	0	-1	0	-1	0	0	-1	0	0	0	0	0	0	0	0	-1	0	0	0	0	1	0	0	0	1	6	7
IHH	ENSG00000163501	0	1	0	0	0	0	0	0	0	0	0	-1	-1	-1	0	0	0	0	0	0	0	0	0	0	0	0	-1	0	0	0	0	-1	0	0	-1	1	6	7
PDPK1	ENSG00000140992	-1	0	0	0	0	0	-1	0	0	0	0	-1	-1	-1	0	-1	0	0	0	0	0	0	0	0	0	0	0	0	0	0	0	0	0	-1	0	0	7	7
CTSK	ENSG00000143387	1	1	0	0	0	0	0	0	0	0	0	0	1	0	0	0	0	0	0	0	0	1	0	0	0	0	0	1	0	0	0	0	0	1	0	6	0	6
APAF1	ENSG00000120868	0	1	0	0	1	0	-1	0	0	0	0	0	0	1	0	1	0	0	0	0	0	0	1	0	0	0	0	0	0	0	0	0	0	0	0	5	1	6
AMELX	ENSG00000125363	0	0	0	1	0	0	0	0	0	0	0	0	1	1	0	0	1	0	0	0	0	0	0	-1	1	0	0	0	0	0	0	0	0	0	0	5	1	6
FGFRL1	ENSG00000127418	0	0	0	0	0	1	0	0	0	0	0	0	0	0	0	1	-1	0	1	0	0	1	0	0	0	1	0	0	0	0	0	0	0	0	0	5	1	6
SKI	ENSG00000157933	0	0	0	0	1	0	0	0	0	0	0	0	0	0	0	1	0	0	1	0	0	0	0	0	0	1	0	0	0	1	0	0	0	-1	0	5	1	6
SLC39A13	ENSG00000165915	0	0	0	0	0	0	1	0	0	0	0	0	1	-1	0	0	0	0	1	1	0	0	1	0	0	0	0	0	0	0	0	0	0	0	0	5	1	6
DCTN5	ENSG00000166847	0	0	1	1	0	0	0	0	0	0	1	0	0	0	0	-1	1	0	0	0	0	0	0	0	0	0	1	0	0	0	0	0	0	0	0	5	1	6
DNAH5	ENSG00000039139	0	0	0	0	0	1	0	0	0	0	0	0	0	1	0	1	0	-1	0	0	0	0	-1	0	0	0	0	0	0	1	0	0	0	0	0	4	2	6
PAPPA2	ENSG00000116183	0	0	0	0	0	0	0	0	0	0	1	0	0	1	0	1	0	0	0	0	0	0	0	-1	0	0	0	1	0	0	0	0	0	0	-1	4	2	6
LPAR4	ENSG00000147145	0	-1	0	0	0	0	1	0	0	0	0	0	0	1	0	0	0	0	0	0	0	0	0	0	0	0	0	1	0	0	-1	1	0	0	0	4	2	6
NPR2	ENSG00000159899	0	0	0	0	0	0	1	0	0	0	0	0	1	-1	0	1	0	-1	0	0	0	0	0	0	0	0	0	1	0	0	0	0	0	0	0	4	2	6
LMNA	ENSG00000160789	0	-1	0	0	0	0	0	0	0	0	0	0	0	0	0	1	0	1	0	1	0	1	0	-1	0	0	0	0	0	0	0	0	0	0	0	4	2	6
ZNF366	ENSG00000178175	0	0	0	0	0	0	0	0	0	0	0	0	-1	1	0	0	0	-1	1	1	0	0	0	0	0	0	0	1	0	0	0	0	0	0	0	4	2	6
PFAS	ENSG00000178921	0	0	0	0	0	1	0	0	0	0	0	0	0	0	0	0	0	0	0	0	0	-1	0	1	0	1	1	0	0	0	-1	0	0	0	0	4	2	6
CEP290	ENSG00000198707	0	1	0	0	0	0	0	0	0	0	0	0	1	0	-1	0	0	0	0	0	0	0	0	0	0	1	-1	0	0	1	0	0	0	0	0	4	2	6
CTNNB1	ENSG00000168036	1	0	0	0	0	0	-1	0	0	0	0	0	-1	0	0	1	0	0	0	0	0	1	0	0	0	0	0	0	0	0	0	0	0	-1	0	3	3	6
FOXI3	ENSG00000214336	0	0	0	0	0	0	0	0	0	0	0	0	1	1	0	0	0	-1	0	0	-1	-1	0	0	0	0	0	0	0	0	0	1	0	0	0	3	3	6
CREBBP	ENSG00000005339	0	0	0	0	0	0	0	0	-1	0	0	-1	-1	1	0	-1	0	0	0	0	0	0	0	0	0	0	0	0	0	1	0	0	0	0	0	2	4	6
FGFR3	ENSG00000068078	0	0	0	0	-1	0	0	0	0	0	0	-1	-1	0	0	1	0	0	-1	0	0	0	0	0	0	0	0	0	0	0	0	0	0	1	0	2	4	6
WNT3A	ENSG00000154342	0	0	0	0	-1	0	0	0	0	1	0	0	0	0	-1	0	0	-1	0	0	0	0	0	0	0	0	1	0	0	0	0	-1	0	0	0	2	4	6
NIPBL	ENSG00000164190	-1	0	0	1	0	0	0	0	0	0	0	1	0	0	-1	0	0	0	0	0	0	0	0	0	0	0	-1	0	0	0	-1	0	0	0	0	2	4	6
PDSS2	ENSG00000164494	-1	0	0	1	0	0	-1	0	1	0	0	0	-1	-1	0	0	0	0	0	0	0	0	0	0	0	0	0	0	0	0	0	0	0	0	0	2	4	6
HHAT	ENSG00000054392	-1	0	0	0	0	0	0	1	-1	0	0	0	-1	-1	0	0	0	0	0	0	0	0	0	0	0	0	0	0	0	0	0	-1	0	0	0	1	5	6
KCNJ13	ENSG00000115474	0	0	0	0	0	0	0	0	1	0	0	-1	0	0	-1	0	0	0	0	-1	-1	0	0	0	0	0	0	0	0	0	0	0	0	0	-1	1	5	6
KAT6B	ENSG00000156650	0	0	0	0	0	0	1	0	0	0	0	0	-1	0	-1	-1	0	0	0	0	0	0	0	0	0	0	-1	0	0	0	-1	0	0	0	0	1	5	6
CYP26B1	ENSG00000003137	1	0	1	0	0	0	0	0	0	0	0	0	0	1	0	0	1	0	0	0	0	0	0	0	0	0	0	0	0	0	0	1	0	0	0	5	0	5
FOXH1	ENSG00000160973	1	0	0	0	0	0	1	0	0	0	0	0	1	0	0	0	0	0	0	0	0	0	0	0	0	0	0	0	0	0	1	0	1	0	0	5	0	5
CYP51A1	ENSG00000001630	0	0	1	0	1	0	0	0	0	0	0	0	-1	0	0	0	0	0	0	0	1	0	0	0	0	0	0	0	0	0	0	0	0	1	0	4	1	5
DLX6	ENSG00000006377	0	0	0	-1	0	0	0	0	0	0	0	0	1	1	0	0	0	0	0	1	0	0	0	0	0	0	0	0	0	0	0	0	0	1	0	4	1	5
BAZ1B	ENSG00000009954	0	0	0	0	0	0	0	0	0	0	0	1	0	0	0	1	0	0	0	0	0	0	0	0	0	0	0	0	0	0	-1	1	0	1	0	4	1	5
ACVR1	ENSG00000115170	0	0	1	0	0	0	0	0	0	0	0	0	0	1	0	0	0	0	0	0	0	1	0	0	1	0	0	0	0	0	0	-1	0	0	0	4	1	5
RDH10	ENSG00000121039	0	0	0	0	0	0	0	0	0	0	0	0	0	1	0	1	0	0	-1	0	0	1	0	0	0	0	0	0	0	1	0	0	0	0	0	4	1	5
GSC	ENSG00000133937	1	0	0	-1	0	0	0	0	0	0	0	0	1	1	0	0	1	0	0	0	0	0	0	0	0	0	0	0	0	0	0	0	0	0	0	4	1	5
TRAPPC10	ENSG00000160218	0	0	0	0	1	0	0	0	-1	0	0	0	0	0	0	1	0	0	0	0	1	0	1	0	0	0	0	0	0	0	0	0	0	0	0	4	1	5
PTPN11	ENSG00000179295	-1	0	0	1	1	0	0	0	0	0	0	0	0	0	0	0	0	0	0	1	0	0	0	0	0	0	0	0	0	0	0	0	0	1	0	4	1	5
GREM2	ENSG00000180875	1	0	1	0	0	0	0	0	0	0	0	1	1	0	0	0	0	0	0	0	0	0	0	0	0	-1	0	0	0	0	0	0	0	0	0	4	1	5
CACNA1S	ENSG00000081248	0	0	0	0	0	0	0	1	0	0	1	0	-1	1	-1	0	0	0	0	0	0	0	0	0	0	0	0	0	0	0	0	0	0	0	0	3	2	5
ARID1A	ENSG00000117713	0	0	0	0	0	0	0	0	0	0	-1	0	0	0	0	1	1	0	0	0	0	0	0	0	0	1	0	0	0	0	-1	0	0	0	0	3	2	5
FRAS1	ENSG00000138759	0	1	0	0	0	0	0	0	0	0	0	0	-1	0	0	0	0	0	-1	0	0	1	0	0	0	0	0	0	0	1	0	0	0	0	0	3	2	5
CNBP	ENSG00000169714	0	0	0	0	0	0	0	0	0	0	0	1	0	1	0	-1	0	0	0	0	0	1	0	0	0	0	0	0	0	0	0	0	0	-1	0	3	2	5
KAT6A	ENSG00000083168	-1	0	0	0	0	0	0	0	0	0	-1	0	-1	1	0	0	0	0	0	0	0	0	0	0	0	0	0	0	0	0	0	1	0	0	0	2	3	5
MMP21	ENSG00000154485	0	0	0	0	0	0	0	0	0	0	0	0	1	0	0	1	-1	0	0	0	0	0	0	0	0	0	-1	-1	0	0	0	0	0	0	0	2	3	5
CTNNBIP1	ENSG00000178585	0	0	0	0	0	0	0	0	0	0	0	0	0	-1	0	0	0	0	0	0	0	0	0	0	0	1	0	0	0	0	1	-1	0	-1	0	2	3	5
MNT	ENSG00000070444	0	0	0	-1	0	0	0	0	0	0	-1	-1	1	0	0	0	0	0	0	0	0	-1	0	0	0	0	0	0	0	0	0	0	0	0	0	1	4	5
DNAI1	ENSG00000122735	-1	0	0	0	0	0	1	0	0	0	0	0	0	-1	0	0	0	0	0	0	0	0	0	0	0	0	-1	0	0	0	0	0	0	-1	0	1	4	5
MAU2	ENSG00000129933	0	0	0	0	0	0	0	0	0	0	-1	0	1	0	0	0	0	-1	0	0	0	-1	0	0	0	0	0	-1	0	0	0	0	0	0	0	1	4	5
RFNG	ENSG00000169733	0	0	0	-1	-1	0	0	0	0	0	0	0	1	0	0	0	0	0	0	0	0	-1	0	0	0	0	0	0	0	0	0	0	0	-1	0	1	4	5
LTBP3	ENSG00000168056	-1	-1	0	0	0	0	0	0	0	0	0	0	0	0	-1	0	0	0	0	0	0	0	0	-1	0	0	0	0	0	0	0	0	0	0	-1	0	5	5
CCN2	ENSG00000118523	0	0	1	0	0	0	0	0	0	0	0	0	0	1	0	0	0	0	1	0	0	0	0	0	0	0	0	1	0	0	0	0	0	0	0	4	0	4
PAX6	ENSG00000007372	1	0	1	0	0	0	0	0	0	0	0	0	0	0	0	0	0	0	0	1	0	-1	0	0	0	0	0	0	0	0	0	0	0	0	0	3	1	4

PAX7	ENSG00000009709	0	1	0	1	0	0	0	0	0	0	0	0	0	1	0	0	0	0	0	0	0	0	0	0	0	0	0	0	-1	0	0	0	0	0	0	0	0	3	1	4
FGF18	ENSG00000156427	0	0	0	0	0	0	0	0	0	0	0	0	1	1	0	0	0	0	0	-1	0	0	0	0	0	0	0	0	0	0	0	0	0	1	0	3	1	4
XYLT1	ENSG00000103489	1	0	0	0	0	0	0	0	0	0	0	0	0	0	0	0	0	0	0	0	1	0	0	0	0	0	0	0	0	0	-1	0	0	-1	0	2	2	4
ANKH	ENSG00000154122	0	0	1	0	0	0	0	0	0	1	0	0	0	0	0	-1	0	0	0	0	0	-1	0	0	0	0	0	0	0	0	0	0	0	0	0	2	2	4
FOXN3	ENSG00000053254	0	-1	0	0	0	0	0	0	0	0	0	0	-1	0	0	0	0	-1	0	0	0	0	0	0	0	0	0	1	0	0	0	0	0	0	0	1	3	4
COL27A1	ENSG00000196739	0	0	0	0	0	0	0	0	0	0	0	0	0	0	0	1	0	0	0	0	0	0	0	0	0	1	0	0	0	1	0	0	0	0	0	3	0	3
SUFU	ENSG00000107882	0	0	0	0	0	0	0	0	0	0	0	0	0	0	1	-1	0	0	0	0	0	0	0	0	0	0	0	0	0	0	0	0	-1	0	0	1	2	3
SC5D	ENSG00000109929	0	0	0	0	0	0	0	0	0	0	1	0	-1	0	0	-1	0	0	0	0	0	0	0	0	0	0	0	0	0	0	0	0	0	0	0	1	2	3
ACVR2A	ENSG00000121989	0	0	0	0	0	0	0	0	0	0	0	0	-1	0	0	-1	0	0	-1	0	0	0	0	0	0	0	0	0	0	0	0	0	0	0	0	0	3	3
ARID5B	ENSG00000150347	0	0	0	0	0	0	0	0	0	0	-1	0	-1	0	0	0	0	-1	0	0	0	0	0	0	0	0	0	0	0	0	0	0	0	0	0	0	3	3
IFITM5	ENSG00000206013	0	1	0	0	0	0	0	0	0	0	0	0	0	-1	0	0	0	0	0	0	0	0	0	0	0	0	0	0	0	0	0	0	0	0	0	1	1	2
	#poor	115	130	112	89	75	77	88	89	75	72	54	46	60	42	18	40	55	34	34	39	26	19	31	41	33	15	24	21	10	7	24	14	17	7	1			
	#favorable	87	49	46	47	52	49	25	21	29	21	33	40	23	39	58	34	17	35	33	25	32	33	19	9	16	25	15	16	26	29	8	11	3	11	5			
	#total	202	179	158	136	127	126	113	110	104	93	87	86	83	81	76	74	72	69	67	64	58	52	50	50	49	40	39	37	36	36	32	25	20	18	6			

Supplementary Table 5: JIG orthologues across pre-gnathostome and gnathostome species.

pregnathostome orthologues

gnathostome-specific orthologues

network HUBS

GENES	*Homo sapiens*	*Mus musculus*	*Gallus gallus*	*Xenopus laevis*	*Danio rerio*	*Callorhinchus milli*	Petromyzon spp.	Ciona spp.	Brachiostoma spp.	Strongylocentrotus spp.	Hydra spp.
ABHD5	NP_057090.2	NP_080455.1	NP_001265074.1	NP_001086565.1	XP_003200181.1	XP_007899088.1					
ACVR1	NP_001104537.1	NP_031420.2	XP_015145613.1	NP_001083727.1	NP_571420.1	XP_007896316.1	XP_032822706.1				
ACVR2A	NP_001607.1	NP_031422.3	NP_990698.1	XP_018089695.1	NP_001103748.2	XP_007894547.1					
ALDH1A2	NP_003879.2	NP_033048.2	NP_990326.1	NP_001084244.1	NP_571925.1	XP_007904660.1	XP_032805864.1				
ALKBH1	NP_006011.2	NP_001096035.1	NP_001026723.1	NP_001087992.1	NP_001018527.1	XP_007904240.1	XP_032827708.1				
ALX1	NP_008913.2	NP_766141.1	XP_025010336.1	NP_001079116.1	NP_001038539.1	XP_007901967.1					
AMBN	NP_057603.1	NP_033794.1		NP_001083489.1	NP_001138709.1						
AMELX	NP_001133.1	NP_001075447.1									
ANKH	NP_473368.1	NP_065065.3	NP_001012580.1	NP_001083924.1	NP_919351.1	XP_007899922.1	XP_032807388.1		XP_002596000.1		
AP2B1	NP_001025177.1	NP_001030931.1	XP_415772	NP_001080473.1	NP_956213.2	XP_007894750.1	XP_032823633.1	XP_002122416.3	XP_002610773.1	XP_030848114	XP_012563108.1
APAF1	NP_037361.1	NP_001036023.1	XP_416167.3	NP_001085834.1	NP_571683.1	XP_007905933.1	XP_032812741.1		XP_002598365.1	XP_030844937.1	XP_012566478.1
APC	NP_000029.2	NP_031488.2	XP_004949340.1	XP_018099812.1	NP_001137312.1	XP_007895594.1	XP_032829901.1	XP_018668497.1	XP_002596549.1	XP_030829439.1	XP_012554796.1
ARHGAP29	NP_004806.3	NP_001343453.1	XP_015146118.1	XP_018115106.1	XP_005163371.1	XP_007885317.1					
ARID1A	NP_006006.3	NP_001074288.1	XP_015153042.1	XP_018104067.1	XP_021323975.1	XP_007893121.1					
ARID5B	NP_115575.1	NP_076087.2	XP_015152937.2	XP_018080600.1	NP_001073670.1	XP_007910615.1					
ARSB	NP_000037.2	NP_033842.3	XP_003642960.1	XP_018120981.1	XP_021324609.1	XP_007890415.1	XP_032812086.1	XP_002127641.1	XP_002592276.1	XP_030842602.1	
ASAP1	NP_060952.2	NP_034156.2	XP_425945.4	XP_018122422.1	XP_009295587.2	XP_007901636.1	XP_032817646.1	XP_026695401.1	XP_035657434.1	XP_011681564.1	XP_012556219.1
ASXL1	NP_056153.2	NP_001035028.1	XP_015152084.1	NP_001086579.1	XP_001341242.2	XP_007886567.1					
ATMIN	NP_056066.2	NP_808368.3	NP_001362358.1	XP_018113369.1	NP_001093512.1	XP_007887411.1	XP_032816122.1	XP_002126586.1			XP_012554135.1
ATP11A	NP_056020.2	NP_001280596.1	XP_015133381.1	XP_018105328.1	XP_021332775.1	XP_007889953.1					
ATPAF2	NP_663729.1	NP_663402.2	XP_414815.1	NP_001090060.1	NP_001076290.1	XP_007903962.1	XP_032816840.1	XP_002131815.1	XP_035695560.1	XP_030841010.1	XP_004209949.1
ATR	NP_001175.2	NP_063917.1	XP_015132446.1	XP_018117212.1	XP_696163.5	XP_007891182.1	XP_032809792.1	XP_018673125.1	XP_002594547.1	XP_030832385.1	XP_012559395.1
B9D2	NP_085055.2	NP_001349083.1	XP_025000288.1	NP_001085984.1	NP_001002394.1	XP_007894621.1	XP_032822864.1	XP_002131657.2	XP_035678285.1	XP_781811.1	XP_012564272.1
BAZ1B	NP_115784.1	NP_035844.2	XP_001233717.4	XP_018104542.1	NP_001334601.1	XP_007894624.1	XP_032803856.1	XP_018670952.1	XP_002591894.1	XP_030849368.1	XP_012565366.1
BCL11B	NP_612808.1	NP_067374.2	XP_003641457.1	XP_018085985.1	XP_009291236.1	XP_007903122.1					
BMP4	NP_001193.2	NP_031580.2	NP_990568.3	NP_001095263.1	NP_571417.1	XP_007886339.1					
BMP5	NP_066551.1	NP_031581.2	NP_990479.1	XP_018118958.1	NP_957345.1	XP_007892257.1					
BMP7	NP_001710.1	NP_031583.2	XP_417496.5	NP_001080866.1	NP_571396.1	XP_007901604.1					
BNC2	NP_060107.3	NP_766458.3	XP_015135686.1	XP_018112777.1	NP_001314681.1	XP_007890780.1	XP_032803547.1	XP_009861392.1			
BRAF	NP_001361187.1	NP_647455.3	NP_990633.1	NP_001087444.1	NP_001311445.1	XP_007896790.1	XP_032819210.1			XP_003725081.2	XP_002156903.3
BRD4	NP_490597.1	NP_065254.3	XP_015128409.1	XP_018106485.1	NP_001104751.1	XP_007885536.1					
CACNA1S	NP_000060.2	NP_055008.2	XP_024999440.1	OCT94594.1	NP_999891.1	XP_007892902.1					
CASK	NP_001119527.1	NP_033936.2	XP_005301824.1	NP_001087107.1	NP_694420.1	XP_007899830.1	XP_032835117.1	XP_026690102.1	XP_002607558.1	XP_030837409.1	XP_002163427.3
CBFB	NP_074036.1	NP_071704.3	NP_989901.2	NP_001087047.1	NP_954679.1	NP_001279053.1	XP_032816492.1	NP_001071672.1	XP_002602848.1	NP_001035104.1	XP_004207755.1
CCDC39	NP_852091.1	NP_080498.1	XP_422777.4	XP_018119161.1	XP_001344625.2	XP_007905952.1	XP_032830900.1	XP_009860998.2	XP_002593455.1	XP_030837980.1	
CCND1	NP_444284.1	NP_031657.1	NP_990712.1	NP_001080245.1	NP_571100.1	AFK11583.1					
CDC73	NP_078805.3	NP_666103.1	NP_001026436.1	NP_001079512.1	NP_001278325.1	XP_007893862.1	XP_032802599.1	XP_002120818.2	XP_035683352.1	XP_001176791.2	XP_002158414.3
CDK10	NP_443714.3	NP_919428.1	NP_001103409.1	NP_001091165.1	NP_001017622.2	NP_001279121.1	XP_032834830.1	XP_009861642.1	XP_035680341.1	XP_011681273.2	XP_012558761.1
CDON	NP_001230526.1	NP_067314.2	XP_417853.3	NP_001079158.1	NP_001075097.1	XP_007894240.1					
CEP290	NP_079390.3	NP_666121.2	XP_004937659.1	NP_001085087.1	NP_001161739.1	XP_007893519.1	XP_032834088.1	XP_018670980.1	XP_002590663.1	XP_030844423.1	XP_012566704.1
CHD1	NP_001261.2	NP_031716.2	XP_015155950.1	XP_018119975.1	NP_001121770.2	XP_007891302.1					
CHRD	NP_003732.2	NP_034023.1	NP_990311.2	NP_001081778.1	NP_571048.2	XP_007900570.1	XP_032827777.1	XP_026690585.1	ABG66525.1	XP_030843065.1	XP_002158106.2
CHST11	NP_060883.1	NP_067414.2	XP_025007655.1	XP_018107832.1	NP_997989.2	XP_007893480.1					
CHTOP	NP_056422.2	NP_001280705.1	XP_024999255.1	NP_001079615.1	NP_955840.1	NP_001279029.1	XP_032809833.1	XP_002127341.3	XP_035671217.1	XP_788869.4	XP_002156847.1
CHUK	NP_001269.3	NP_031726.2	NP_001012922.1	NP_001086127.1	NP_956611.1	XP_007896804.1					
CNBP	NP_003409.1	NP_038521.1	NP_990238.1	NP_001084082.1	NP_001313334.1	XP_007901526.1	XP_032828325.1				
COL11A1	NP_001845.3	NP_031755.2	XP_422303.3	XP_018114134.1	XP_005162870.1	XP_007885476.1					
COL1A1	NP_000079.2	NP_031768.2	XP_024999899.1	NP_001080821.1	NP_954684.1	XP_007908905.1					
COL27A1	NP_116277.2	NP_079961.3	XP_015146330.1	XP_018088698.1	NP_001074044.1	XP_007898565.1					
COL2A1	NP_001835.3	NP_112440.2	XP_025001042.1	NP_001081260.1	NP_571367.1	XP_007908719.1	XP_032813113.1	XP_026690723.1		NP_999674.1	
CPLANE1	NP_075561.3	NP_001156378.1	XP_015133013.1	XP_018121837.1	XP_021334904.1	XP_007909187.1	XP_032826472.1	XP_026692817.1	XP_002594230.1	XP_030852004.1	
CPLANE2	NP_112169.2	NP_001074643.1	XP_015152521.1	NP_001085897.1	NP_001004649.1	XP_007905417.1	XP_032800570.1	XP_009861064.1		XP_011675035.2	XP_012563355.1
CREBBP	NP_004371.2	NP_001020603.1	XP_015150110.2	NP_001088637.1	XP_021329192.1	XP_007891802.1					
CRIM1	NP_057525.1	NP_056615.1	NP_989756.1	NP_001163917.1	NP_997986.1	XP_007896627.1	XP_032803737.1	XP_026689622.1	XP_035672184.1	XP_030854614.1	
CRKL	NP_005198.1	NP_031790.2	XP_021268197.1	NP_001087595.1	NP_998703.1	XP_007908281.1	XP_032802890.1	XP_002130707.1	XP_035691933.1	XP_011683040.1	XP_012561143.1
CTGF	NP_001892.1	NP_034347.2	NP_989605.1	NP_001081697.1	NP_001015041.1	XP_007897423.1	XP_032829951.1				
CTNNB1	NP_001895.1	NP_033948.1	NP_990412.2	NP_001084045.1	NP_571134.2	XP_007899861.1					
CTNNBIP1	NP_064633.1	NP_075954.1	NP_001289087.1	NP_001086620.1	NP_571669.1	XP_007900324.1					
CTSK	NP_000387.1	NP_031828.2	NP_990302.2	XP_018088930.1	NP_001017778.1	XP_007899280.1					
CYB561	NP_001906.3	NP_031831.2	XP_003642826.1	NP_001089047.1	NP_001122216.2	XP_007905533.1	XP_032800906.1		XP_035688206.1	XP_030846361.1	
CYP26B1	NP_063938.1	NP_780684.1	XP_015141554.1	XP_018082655.1	NP_997831.1	XP_007904867.1	XP_032808017.1				
CYP51A1	NP_000777.1	NP_064394.2	XP_015136878.1	NP_001091285.1		XP_007895734.1	XP_032816590.1		XP_002606163.1	XP_030851345.1	
DAAM2	NP_001188356.1	NP_001008232.2	XP_025004642.1	XP_018118186.1	XP_001338908.5	XP_007909292.1	XP_032803809.1	XP_026692086.1	XP_002604562.1	XP_030855636.1	XP_012558320.1
DCTN5	NP_115875.1	NP_067621.3	NP_001185568.1	NP_001086487.1	NP_001002497.1	XP_007904011.1	XP_032832114.1	XP_002131615.1	XP_002605052.1	XP_030840147.1	XP_002155568.3
DHRS3	NP_004744.2	NP_035433.1	NP_001264839.1	XP_018083410.1	NP_001003477.1	XP_007885177.1	XP_032824210.1		XP_002586689.1		
DISP1	NP_001364158.1	NP_081142.3	XP_004935360.1	XP_018120452.1	NP_997965.1	XP_007896461.1					
DKK1	NP_036374.1	NP_034181.2	XP_015134195.1	NP_001079061.1	NP_571078.1	XP_007895204.1					
DLG1	NP_001353136.1	NP_031888.2	XP_015147091.1	XP_018119117.1	NP_955820.1	XP_007896055.1					
DLX2	NP_004396.1	NP_034184.1	XP_025008373.1	NP_001084032.1	NP_571386.2	XP_007888184.1					
DLX5	NP_005212.1	NP_034186.2	NP_989490.1	XP_018124683.1	NP_571381.2	XP_007902942.1					
DLX6	NP_005213.3	NP_034187.1	XP_015137078.1	NP_001090284.1	NP_571398.1	XP_007902943.1					
DNAH11	NP_001264044.1	NP_034190.3	XP_415585.5	XP_018122854.1	XP_009290539.1	XP_007901515.1					
DNAH5	NP_001360.1	NP_579943.3	XP_025003476.1	XP_018123134.1	XP_021322524.1	XP_007898716.1	XP_032816356.1	XP_018671051.1		XP_030831333.1	
DNAI1	NP_036276.1	NP_780347.2	XP_003643013.1	XP_018100839.1	XP_021324725.1	XP_007895318.1	XP_032809556.1	XP_002129294.1	XP_002609708.1	XP_030833320.1	XP_012559387.1
DNASE1L2	NP_001365.1	NP_079994.2	XP_025011158.1	XP_018092033.1	NP_001002674.1	XP_007891950.1	XP_032823148.1		XP_035669754.1	XP_798848.3	XP_012565167.1
DOCK1	NP_001277152.2	NP_001028592.1	XP_025007693.1	XP_018080660.1	NP_001103481.1	XP_007906144.1					
DPH1	NP_001374.4	NP_652762.2	NP_001025887.2	XP_012812029.1	NP_001017861.1	XP_007904382.1	XP_032823710.1	XP_026691241.1	XP_002605608.1	XP_030848155.1	XP_002161454.2

DRC1	NP_659475.2	NP_001028632.1	XP_420016.3	NP_001079840.1	NP_001120940.1	XP_007898606.1	XP_032817535.1	XP_004226120.4		XP_030830868.1	
DVL3	NP_004414.3	NP_031915.2	XP_015147052.1	NP_001086098.1	XP_001920242.3	XP_007905969.1					
DYNC2H1	NP_001368.2	NP_084127.2	XP_417173.2	XP_018101055.1	XP_021322179.1	XP_007902643.1	XP_032832089.1	XP_026693267.1	XP_019625235.1	XP_030836572.1	XP_012558919.1
ECE1	NP_001388.1	NP_955011.1	XP_015152737.1	NP_001080378.1	NP_001071260.1	XP_007896015.1					
EDN1	NP_001946.3	NP_034234.1	XP_418943.2	NP_001090567.1	NP_571594.1	XP_007904565.1	XP_032817360.1				
EDNRA	NP_001948.1	NP_034462.1	NP_989450.1	NP_001080650.1	NP_001092915.2	XP_007897347.1	XP_032811286.1				
EDNRB	NP_001116131.1	NP_031930.1	NP_001001127.1	NP_001079707.1	NP_571272.1	XP_007900764.1	XP_032812242.1				
EGFR	NP_005219.2	NP_997538	NP_990828.2	XP_018123025.1	NP_919405.1	XP_007895818.1					
EGR2	NP_000390.2	NP_034248.2	XP_025007817.1	NP_001079248.1	NP_571072.2	XP_007895410.1					
ELP1	NP_003631.2	NP_080355.2	XP_001231708.2	XP_018095285.1	XP_689534.5	XP_007904787.1	XP_032824390.1	XP_009859423.1	XP_035669084.1	XP_030832738.1	XP_012557166.1
EPS8L3	NP_573444.2	NP_598628.1	XP_024999597.1	XP_018096619.1	NP_001315008.1	XP_007897795.1	XP_032833964.1	XP_026694557.1	XP_002586560.1	XP_030849025.1	XP_012557569.1
EYA1	NP_000494.2	NP_034294.2	XP_015138368.1	XP_018097003.1	NP_571268.1	XP_007885157.1					
FAM20C	NP_064608.2	NP_085042.2	XP_015149804.2	XP_018091900.1	XP_688892.5	XP_007891748.1	XP_032822771.1	XP_002122861.4	XP_002606109.1	XP_030839755.1	
FBLN1	NP_006477.3	NP_034310.2	NP_989496.1	NP_001089430.1	NP_571117.2	XP_007892884.1					
FBN1	NP_000129.3	NP_032019.2	XP_015147420.1	XP_018108582.1	XP_017207479.2	XP_007909428.1					
FBN2	NP_001990.2	NP_034311.2	XP_424715.3	XP_018119620.1	XP_021325049.1	XP_007890722.1	XP_032824596.1				
FGF10	NP_004456.1	NP_032028.1	NP_990027.1	NP_001131038.1	NP_878290.1	XP_007890403.1	XP_032806446.1				
FGF18	NP_003853.1	NP_032031.1	NP_990045.1	NP_001008163.1	NP_001013282.1	XP_007889620.1					
FGF8	NP_149355.1	NP_034335.1	NP_001012785.1	NP_001083904.1	NP_571356.2	XP_007904873.1					
FGF9	NP_002001.1	NP_038546.2	NP_989730.1	NP_001079276.1		XP_007898849.1					
FGFR1	NP_075598.2	NP_034336.2	NP_990841.1	NP_001081157.1	NP_694494.2	XP_007909759.1					
FGFR2	NP_000132.3	NP_034337.2	NP_990650.1	NP_001084132.1	NP_001229933.1	XP_007902087.1					
FGFR3	NP_000133.1	NP_032036.2	NP_990840.2	NP_001084170.1	NP_571681.2	XP_007896238.1					
FGFRL1	NP_001004356.1	NP_473412.1	XP_025005238.1	NP_001086420.1	NP_956670.1	XP_007896240.1	XP_032806318.1	XP_026690297.1	CAI61931.1	NP_001165523.1	
FIGN	NP_060556.2	NP_068362.1	XP_004942874.1	XP_018091343.1	NP_001018411.1	XP_007888032.1					
FLVCR1	NP_054772.1	NP_932142.2	XP_419425.2	XP_018120489.1	NP_001020693.1	XP_007908741.1					
FOXC1	NP_001444.2	NP_032618.2	NP_990337.1	NP_001089846.1		XP_007887802.1					
FOXC2	NP_005242.1	NP_038547.2	NP_990469.1	NP_001083920.1		XP_007887427.1					
FOXD3	NP_036315.1	NP_034555.3	NP_990282.2	NP_001079026.1	NP_571365.2	XP_007907140.1	XP_032822101.1	XP_009859457.3	XP_002602560.1	XP_003724544.1	
FOXF2	NP_001443.1	NP_034355.2	XP_015137672.2	XP_018123225.1	XP_003200754.1	XP_007887803.1					
FOXH1	NP_003914.1	NP_032015.1	XP_015137671.2	NP_001081820.1	NP_571577.1	XP_007910257.1	XP_032834948.1	NP_001071992.1	ACE79158.1		
FOXI3	NP_001129121.1	NP_001094934.1	XP_425185.1	XP_018111695.1	NP_944599.2	XP_007889609.1					
FOXN3	NP_005188.2	NP_899009.2	XP_015143229.1	NP_001154856.1	NP_001038728.2	XP_007904191.1					
FRAS1	NP_079350.5	NP_780682.3	XP_025006059.1	XP_018124333.1	NP_001124312.1	XP_007895210.1	XP_032805666.1	XP_026692906.1	XP_002601902.1	XP_030835000.1	XP_012562678.1
FREM1	NP_001366010.1	NP_001185740.1	XP_004949159.2	XP_018113257.1	NP_001177237.1	XP_007890245.1	XP_032822130.1	XP_002124581.3		XP_030851816.1	
FUZ	NP_079405.2	NP_081652.2	XP_015151679.1	XP_018081840.1	XP_001337543.1	XP_007910217.1	XP_032833903.1	XP_002130176.1	XP_002604807.1	XP_003723986.2	XP_002154880.2
GAD1	NP_000808.2	NP_032103.2	NP_990244.1	XP_018089468.1	NP_919400.1	XP_007888154.1	XP_032821372.1	XP_026696077.1	XP_002592141.1	XP_784856.2	
GAS1	NP_002039.2	NP_032112.1	XP_004949280.1	XP_018099363.1	NP_001071269.1	XP_007906242.1	XP_032824729.1	XP_002123239.1			
GAS2L2	NP_644814.1	NP_001013781.1	XP_004946562.1	XP_018105119.1	XP_002662207.2	XP_007900483.1					
GJA1	NP_000156.1	NP_034418	NP_989917.1	NP_001079129.1	NP_571113.1	XP_007897861.1					
GJB2	NP_003995.2	NP_032151	NP_001257745.1	NP_001080478.1	NP_997990.2	XP_007903773.1	XP_032828672.1				
GKN1	NP_062563.4	NP_079742.1	XP_004947629.1	XP_018107223.1							
GLDC	NP_000161.2	NP_613061.1	XP_015135548.1	NP_001080141.1	NP_955848.1	XP_007902171.1	XP_032826442.1	XP_002120534.1		XP_003724415.1	XP_002160817.2
GLG1	NP_001139139.1	NP_033175.1	NP_990827.1	XP_018116137.1	XP_001332484.4	XP_007902868.1	XP_032830405.1	XP_026689671.1	XP_002612434.1	XP_030831332.1	XP_012560055.1
GLI2	NP_005261.2	NP_001074594.1	NP_001258830.1	NP_001081894.1	NP_571042.1	XP_007894561.1					
GLI3	NP_000159.3	NP_032156.2	NP_001258832.1	NP_001081440.1	NP_991291.1	XP_007896289.1					
GPC3	NP_001158089.1	NP_057906.2	XP_025006018.1	XP_002932665.2	NP_001292547.1	XP_007890697.1					
GRB2	NP_002077.1	NP_032189.1	NP_989742.1	NP_001084357.1	NP_998200.1	XP_007886673.1	XP_032826167.1	XP_002125384.4	XP_019642516.1	XP_001193089.2	XP_002166949.2
GREM2	NP_071914.3	NP_035955.1	XP_419552.3	NP_001083746.1	NP_001017704.1	XP_007892101.1					
GSC	NP_776248.1	NP_034481.1	NP_990662.1	NP_001081278.1	NP_571092.1	XP_007910192.1	XP_032820078.1	NP_001071728.1	AAF97935.1	NP_999663.1	NP_001296713.1
HAND1	NP_004812.1	NP_032239.1	NP_990296.1	NP_001079128.1		XP_007906292.1					
HAND2	NP_068808.1	NP_034532.3	NP_990297.2	NP_001079108.1	NP_571701.3	XP_007894139.1					
HAPLN1	NP_001875.1	NP_038528.3	NP_990813.1	XP_018099885.1	NP_001007791.2	XP_007897935.1					
HAT1	NP_003633.2	NP_080391.2	NP_989538.1	XP_018094344.1	NP_001004572.1	XP_007908137.1	XP_032821290.1	XP_002124657.1	XP_002602992.1	XP_030842939.1	XP_012564660.1
HHAT	NP_060664.2	NP_659130.2	XP_001234561.1	XP_018118052.1	NP_001108362.2	XP_007890669.1	XP_032818807.1	XP_002130477.1	XP_002605788.1	XP_030854460.1	XP_012559282.1
HOXA2	NP_006726.1	NP_034581.1	NP_990481.1	NP_001079219.1	NP_571181.1	XP_007898687.1					
HOXA3	NP_705895.1	NP_034582.1	XP_025002790.1	NP_001080293.1	NP_571609.1	XP_007898681.1					
HRAS	NP_005334.1	NP_032310.2	NP_990623	NP_001084278.1	NP_001017623	XP_007907536.1					
HSPB11	NP_057210.2	NP_082670.1	NP_001264542.1	NP_001087826.1	NP_001092897.1	XP_007907160.1			XP_002603858.1	XP_030852059.1	
HSPG2	NP_005520.4	XP_030109088.1	XP_015152746	XP_018081990.1	XP_009295216.1	XP_007896099	XP_032824291.1	XP_018666843.1	XP_019637175.1	XP_030842937.1	XP_004211405.2
IBSP	NP_004958.2	NP_032344.2	NP_990493.1	XP_018098254.1							
IDE	NP_004960.2	NP_112419.4	XP_004942214.3	XP_018082897.1	XP_005169584.1	XP_007894712.1	XP_032806215.1	XP_009861694.1	XP_035672073.1	XP_030848810.1	XP_012559703.1
IDS	NP_000193.1	NP_034628.2	XP_015133790.1	XP_018085386.1	NP_001073537.1	XP_007891097.1	XP_032800625.1	XP_009861788.1	XP_035682477.1	XP_785170.4	
IDUA	NP_000194.2	NP_032351.2	NP_001026604.1	NP_001087031.1	XP_001923689.3	XP_007905890.1					
IFITM5	NP_001020466.1	NP_444318.1	NP_001186427.1	XP_018112954.1	NP_001170783.1	XP_007902982.1					
IFT140	NP_055529.2	NP_598887.3	NP_001012810.2	XP_018091986.1	XP_695732.4	XP_007891874.1	XP_032824144.1	XP_002125448.2	XP_002607214.1	XP_030840366.1	XP_012555320.1
IFT27	NP_001171172.1	NP_080207.1	NP_001264310.1	NP_001090381.1	NP_001008588.1	NP_001279585.1	XP_032817047.1	XP_009858222.1	XP_002588733.1	XP_030847703.1	XP_004212778.2
IFT57	NP_060480.1	NP_082956.2	XP_416624.4	NP_001108268.1	NP_001001832.1	XP_007895758.1	XP_032822969.1	XP_002121351.1	XP_002604500.1	XP_030833864.1	XP_002154458.3
IHH	NP_002172.2	NP_034674.2	NP_990288.1	NP_001079262.1	NP_001030165.2	XP_007909552.1					
IRF6	NP_006138.1	NP_058547.2	XP_024999581.1	NP_001085345.1	NP_956892.1	XP_007897748.1					
ITGB1	NP_002202	NP_034708.1	NP_001034343.2	NP_001081286.1	NP_001030143.1	XP_007907125.1					
JAG1	NP_000205.1	NP_038850.1	XP_415035.4	NP_001083776.1	NP_571936.1	XP_007903695.1	XP_032803601.1				
JAG2	NP_002217.3	NP_034718.2	XP_001235689.2	XP_018087787.1	NP_571937.1	XP_007909754.1					
KAT14	NP_065397.3	NP_852082.2	NP_001186370.1	XP_018118382.1	NP_001002699.2	XP_007892627.1	XP_032804461.1	XP_002121916.1	XP_002602499.1	XP_030828197.1	
KAT6A	NP_006757.2	NP_001074618.1	XP_424402.4	XP_018106960.1	NP_001116784.4	XP_007908442.1					
KAT6B	NP_036462.2	NP_059507.2	XP_004942127.3	XP_018110516.1	XP_697383.4	XP_007896926.1					
KCNJ13	NP_002233.2	NP_001103697.1	XP_015132697.1	XP_018119383.1	NP_001039014.1	XP_007887389.1	XP_032818336.1				
KCNJ2	NP_000882.1	NP_032451.1	NP_001310133.1	XP_018096315.1	NP_001318145.1	XP_007886827.1					
KDF1	NP_689578.2	NP_598468.2	XP_015153075.1	NP_001108297.1	XP_001338378.3	XP_007893132.1					
KDR	NP_002244.1	NP_034742.2	NP_001004368.1	XP_018085457.1	NP_001019824.2	XP_007890947.1					
KIF3A	NP_001287720.1	NP_032469.2	NP_001025793.1	NP_001084268	NP_001017604.2	XP_007900170.1					
KLF2	NP_057354.1	NP_032478.2	NP_001305352.1	NP_001080430.1	NP_571931.2	XP_007899255.1					
KMT2D	NP_003473.3	NP_001028448.3	XP_025001253.1	XP_018102859.1	XP_009295228.1	XP_007907792.1					
LIMK2	NP_005560.1	NP_034848.1	NP_990446.1	NP_001082219.1	NP_001002651.1	XP_007909768.1					
LMNA	NP_733821.1	NP_062263.1	NP_990618.1	NP_001095210.1	NP_694503.1	XP_007906490.1	XP_032806857.1				

**LOXL3**	NP_115992.1	NP_038614.2	XP_015156777.1	XP_018098412.1	NP_001139156.1	XP_007908441.1				
**LPAR4**	NP_001264929.1	NP_780480.2	NP_001264978.1	NP_001090494.1	XP_001334713.1	XP_007890382.1				
**LRP2**	NP_004516.2	NP_001074557.1	XP_004942820.1	XP_018091370.1	NP_001181916.1	XP_007888391.1	XP_032801941.1	XP_026691732.1	XP_002598410.1	XP_030830689.1	
**LRRK1**	NP_078928.3	NP_666303.3	NP_001274122.2	XP_018108760.1	XP_021333791.1	XP_007905552.1	XP_032806825.1	XP_026690738.1	XP_002609807.1	XP_030832414.1	XP_012555867.1
**LTBP1**	NP_996826.2	NP_064303.2	XP_015139206.1	XP_018118508.1	XP_017207300.1	XP_007896642.1	XP_032803602.1				
**LTBP3**	NP_001123616.1	NP_032546.2	XP_025004462.1	XP_002935409.2	NP_001244070.1	XP_007884094.1					
**LYN**	NP_002341.1	NP_001104566.1	NP_001006390.1	XP_018121674.1	NP_001004543.1	XP_007889189.1					
**MAP3K7**	NP_663304.1	NP_033342.1	XP_015140169.1	NP_001084359.1	NP_001018586.1	XP_007891110.1	XP_032829518.1	NP_001071829.1	XP_002589769.1	XP_030850465.1	XP_002162138.2
**MAPK1**	NP_002736.3	NP_036079.1	NP_989481.1	NP_001081344.1	NP_878308.2	XP_007906800					
**MAU2**	NP_056144.3	NP_083269.4	XP_425908.2	NP_001124425.1	XP_021322113.1	XP_007897013.1	XP_032829637.1		XP_002609698.1	XP_030833209.1	XP_012560519.1
**MBTPS1**	NP_003782.1	NP_062683.3	XP_003641945.1	NP_001087381.2	NP_954683.2	XP_007902889.1	XP_032813570.1	XP_002122807.1	XP_002594247.1	XP_030831757.1	XP_002164301.2
**MECP2**	NP_001104262.1	NP_034918.1	XP_025001604.1	NP_001081854.1	NP_997901.1	XP_007909886.1	XP_032821181.1				
**MEF2C**	NP_002388.2	NP_001334493.1	XP_004949467.1	XP_018099860.1	XP_017211668.1	XP_007891245.1					
**MEGF8**	NP_001258867.1	NP_001153872.1	XP_015144416.1	XP_018081796.1	XP_005158088.1	XP_007909276.1	XP_032835707.1	XP_026689375.1	XP_002613359.1	XP_030834386.1	XP_012556758.1
**MFSD12**	NP_778148.2	NP_082933.2	XP_418193.3	XP_018099000.1	NP_001038437.1	XP_007908362.1	XP_032829644.1	XP_002124684.1	XP_035667596.1	XP_030832400.1	XP_012554302.1
**MIGA2**	NP_001316919.1	NP_001229336.1	XP_425337.2	NP_001085347.1	NP_001013575.1	XP_007901035.1					
**MKS1**	NP_060247.2	NP_001034773.2	XP_415705.5	NP_001090590.1	NP_001070841.2	XP_007894621.1	XP_032823388.1	XP_018669336.1	XP_002605664.1	XP_011678596.1	XP_002157754.2
**MMP14**	NP_004986.1	NP_032634.3	XP_015147798.1	NP_001084478.1	NP_919397.1	XP_007901389.1					
**MMP2**	NP_004521.1	NP_032636.1	NP_989751.1	NP_001080697.1	NP_932333.1	XP_007887678.1					
**MMP21**	NP_671724.1	NP_694423.1	XP_015131123.1	NP_001079285.1	NP_001304682.1	XP_007902157.1					
**MN1**	NP_002421.3	NP_001074704.1	XP_003642254	XP_018099537.1	XP_001919775.2	XP_007906045.1					
**MNT**	NP_064706.1	NP_034943.3	XP_015151357.1	XP_018104601.1	NP_001096581.1	XP_007898900.1	XP_032823523.1	XP_009861486.1	XP_002595964.1	XP_003728085.2	XP_012561962.1
**MORC2**	NP_001290185.1	NP_937805.1	XP_015130866.1	XP_018099473.1	NP_001003994.1	XP_007900084.1	XP_032830025.1	XP_002131683.1		XP_030847919.1	XP_012559975.1
**MSX1**	NP_002439.2	NP_034965.2	NP_990819.1	NP_001084367.1	NP_571348.1	XP_007895507.1					
**MTHFD1L**	NP_056255.2	NP_758512.3	XP_004935674.1	XP_018118472.1	NP_001229925.1	XP_007892608.1	XP_032830313.1	XP_026693815.1	XP_035678197.1	XP_030855572.1	XP_004212461.2
**MYH10**	NP_001242941.1	NP_780469.1	XP_015150613.1	XP_018092877.1	XP_683046.6	XP_007886649.1	XP_032817139.1				
**NABP2**	NP_076973.1	NP_081533.1	XP_025001296.1	NP_001084896.1	XP_005172861.1	XP_007888361.1					
**NDST1**	NP_001534.1	NP_032332.2	XP_004945002.1	XP_018107482.1	NP_001311384.1	XP_007906348.1					
**NFATC1**	NP_765975.1	NP_058071.2	XP_015131424.1	XP_018121962.1	NP_001038624.1	XP_007898768.1					
**NFIC**	NP_001231931.1	NP_032714.1	NP_990602.1	NP_001154859.1	XP_005166996.1	XP_007896879.1					
**NIPBL**	NP_597677.2	NP_081983.2	XP_015133005.1	NP_001086437.1	NP_001154919.2	XP_007909505.1	XP_032801038.1	XP_018671410.1	XP_002587265.1	XP_030852099.1	XP_004213193.2
**NISCH**	NP_009115.3	NP_073147.2	XP_015148236.1	XP_018096399.1	XP_002666276.2	XP_007907065.1	XP_032828205.1	XP_002123643.3	XP_035677692.1	XP_030827981.1	XP_004211419.2
**NOG**	NP_005441.1	NP_032737.1	NP_989454.1	XP_018096035.1	NP_571057.1	XP_007891794.1	XP_032828422.1	NP_001071777.1	XP_002595406.1	XP_030855638.1	NP_001296696.1
**NPR2**	NP_003986.2	NP_776149.1	XP_003642967.1	NP_001084176.1	XP_009300100.1	XP_007895384.1					
**OSTM1**	NP_054747.2	NP_766004.1	NP_001026248.1	XP_018118820.1	XP_002665788.2	NP_001279106.1	XP_032832500.1	XP_002128956.1			XP_002169949.2
**OTULIN**	NP_612357.4	NP_001013814.2	XP_004935161.3	XP_018124896.1	XP_001341618.4	XP_007899898.1	XP_032823311.1	XP_018667571.1	XP_002598365.1		
**OTX2**	NP_068374.1	NP_659090.1	NP_989851.2	NP_001084955.1	NP_571326.1	XP_007891617.1					
**PAK1IP1**	NP_060376.2	NP_080826.2	NP_001026170.1	NP_001087421.1	NP_957239.2	XP_007899046.1	XP_032808637.1	XP_026691645.1	XP_002609652.1	XP_795222.3	XP_012559538.1
**PAPPA2**	NP_064714.2	NP_001078845.1	XP_025008932.1	XP_018088392.1	XP_695417.4	XP_007894444.1					
**PAX1**	NP_001244025.1	NP_032806.2	XP_015138914.2	NP_001090451.2	NP_001074061.1	XP_007896543.1					
**PAX3**	NP_852123.1	NP_032807.3	NP_989600.1	NP_001088993.1	NP_571352.1	XP_007887991.1					
**PAX6**	NP_000271.1	NP_038655.1	NP_990397.1	XP_018111894.1	NP_571379.1	XP_007885968.1	XP_032805179.1	NP_001027641.1	CAA11364.1	XP_030845154.1	
**PAX7**	NP_001128726.1	NP_035169.1	XP_015152318.1	XP_018080096.1	XP_009304561.1	XP_007896063.1					
**PAX9**	NP_001359005.1	NP_035171.1	NP_990243.2	NP_001167485.1	NP_571373.1	XP_007891546.1					
**PCSK6**	NP_002561.1	NP_035178.1	XP_025009761.1	NP_001086467.1	XP_021333790.1	XP_007905602.1					
**PDGFRB**	NP_002600.1	NP_032835.2	XP_025010809.1	XP_018110448.1	NP_001177862.1	XP_007906332.1					
**PDPK1**	NP_002604.1	NP_035192.2	NP_001012547.1	XP_018094630.1	NP_991262.1	XP_007904001.1	XP_032822965.1	XP_002124476.1	XP_002606093.1	XP_786576.2	NP_001267831.1
**PDS5B**	NP_055847.1	NP_780519.3	NP_001012845.2	NP_001089658.1	XP_693953.2	XP_007889466.1	XP_032811251.1				
**PDSS2**	NP_065114.3	NP_082048.2	NP_001305370.1	XP_018120896.1	NP_001002351.1	NP_001279445.1	XP_032815024.1	XP_026695230.1	XP_002589797.1	XP_011670674.2	XP_002156194.1
**PFAS**	NP_036525.1	NP_001152991.1	XP_024999398.1	XP_018094887.1	NP_001038667.1	XP_007893212.1	XP_032832973.1	XP_018671183.1	XP_002611349.1	XP_030836182.1	XP_004212171.2
**PGAP1**	NP_079265.2	NP_001156786.1	XP_015144976.1	NP_001087710.1	XP_001922432.2	XP_007888374.1	XP_032820600.1	XP_002131111.1	XP_002600834.1	XP_030840100.1	XP_012555278.1
**PHEX**	NP_000435.3	NP_035207.1	XP_015157829.1	XP_018101754.1	NP_001082818.2	XP_007908778.1	XP_032820142.1				
**PIGV**	NP_060307.2	NP_848813.3	XP_024998857.1	XP_018104933.1	NP_001186989.1	XP_007893122.1	XP_032829362.1	XP_002122985.2	XP_002612582.1	XP_030848488.1	XP_012559454.1
**PITX1**	NP_002644.4	NP_035227.1	XP_015149472.2	NP_001080981.1	NP_001035436.3	XP_007899502.1					
**PITX2**	NP_000316.2	NP_035228.2	NP_990341.1	NP_001017227.1	NP_571050.1	XP_007895188.1					
**PKD1**	NP_001009944.3	NP_038658.2	XP_414854.4	XP_018092070.1	XP_021333081.1	XP_007908643.1	XP_032832098.1	XP_018669270.1	XP_002595676.1		
**PKDCC**	NP_612379.2	NP_598878.2	XP_025004831.1	NP_001091231.2	NP_001155070.1	XP_007890699.1	XP_032811876.1	XP_002128638.1	XP_002590129.1	XP_030854909.1	
**PLXND1**	NP_055918.3	NP_080652.2	XP_414455.4	XP_018114545.1	NP_991260.2	XP_007888683.1	XP_032822371.1				
**POLE4**	NP_063949.2	NP_080158.1	XP_015155279.1	NP_001090193.1	NP_001338727.1	XP_007900747.1	XP_032826578.1	XP_002129269.1		XP_030852301.1	
**POSTN**	NP_006466.2	NP_056599.1	XP_015133099.1	XP_018105718.1	NP_981966.1	XP_007889427.1					
**POU3F3**	NP_006227.1	NP_032926.2	XP_015133414.1	XP_018102574.1	NP_571177.2	XP_007883344.1					
**PRDM16**	NP_071397.3	NP_081780.3	XP_417551.3	XP_018083496.1	XP_001922927.6	XP_007900361.1					
**PRICKLE1**	NP_001138353.1	NP_001028389.1	XP_015137914.1	NP_001082157.1	NP_899185.2	XP_007893374.1					
**PRKRA**	NP_003681.1	NP_036001.1	XP_015155835.1	NP_001079500.1	NP_001082830.1	XP_007888271.1					
**PRRX1**	NP_073207.1	NP_035257.1	NP_001264653.1	XP_018114269.1	NP_999899.1	XP_007893876.1					
**PSIP1**	NP_150091.2	NP_598709.1	NP_001026781.2	NP_001082534.1	XP_005159951.1	XP_007890244.1	XP_032809073.1				
**PSPH**	NP_004568.2	NP_598661.1	NP_001239201.1	NP_001086494.1	NP_001028271.1	XP_007906646.1	XP_032823695.1	XP_002119347.1		XP_030847788.1	XP_002167419.1
**PTCH1**	NP_000255.2	NP_032983.1	NP_990291.2	NP_001082082.1	NP_001292471.1	XP_007898170.1					
**PTH1R**	NP_000307.1	NP_035329.2	NP_001171046.1	XP_018122519.1	NP_571432.1	XP_007908388.1					
**PTHLH**	NP_945316.1	NP_032996.2	XP_025002654.1	XP_018107394.1	NP_001019798.2	XP_007903315.1	XP_032804916.1				
**PTK7**	NP_002812.2	NP_780377.1	NP_001026206.1	NP_001083315.1	XP_021336598.1	XP_007896436.1	XP_032811886.1	XP_018669704.1		XP_030855357.1	
**PTPN11**	NP_002825.3	NP_035332.1	NP_990299.1	XP_018119081.1	NP_956140.1	XP_007900075					
**QSOX1**	NP_002817.2	NP_001020116.1	NP_989456.1	NP_001090556.1	NP_001121836.1	XP_007893719.1					
**RBBP7**	NP_002884.1	NP_033057.3	NP_990001.1	XP_018102361.1	NP_997775.1	XP_007889875.1					
**RDH10**	NP_742034.1	NP_598593.1	NP_001186388.1	NP_001087025.1	NP_001074052.1	XP_007885177.1	XP_032815400.1	XP_002120364.1		XP_011678752.1	
**RFNG**	NP_002908.1	NP_033079.1	NP_990278.1	XP_018089606.1	NP_001001830.2	XP_007886742.1					
**ROBO1**	NP_002932.1	NP_062286.2	XP_015153967.1	NP_001084250.1	NP_001296753.1	XP_007901766.1					
**ROR2**	NP_004551.2	NP_038874.3	NP_001074185.1	XP_018099378.1	XP_689681.6	XP_007898225.1					
**RPGRIP1L**	NP_056087.2	NP_775607.2	XP_025010081.1	XP_018113254.1	NP_001233589.2	XP_007887654.1	XP_032834838.1	XP_018666898.1	XP_002612308.1	XP_030832105.1	XP_012559558.1
**RSPO2**	NP_848660.3	NP_766403.1	NP_001305953.1	NP_001088999.1	NP_001268919.1	XP_007900878.1					
**RYK**	NP_002949.2	NP_038677.3	XP_422674.6	NP_001089445.1	NP_001077295.1	XP_007908285.1	XP_032836167.1	XP_002129769.1		XP_011665108.1	XP_012558645.1
**SATB2**	NP_001165980.1	NP_631885.1	NP_001186039.1	NP_001267548.1	NP_001122004.1	XP_007898262.1					
**SC5D**	NP_008849.2	NP_766357.1	XP_015153662.1	NP_001087137.1	NP_001004630.1	XP_007909349.1	XP_032820533.1	XP_002131054.1	XP_002590254.1	XP_030831120.1	
**SCHIP1**	NP_055390.1	NP_038956.2	XP_004943577.2	XP_018119243.1	NP_001017906.1	XP_007886672.1	XP_032830867.1		XP_002599883.1	XP_030838173.1	
**SETD5**	NP_001073986.1	NP_082661.1	NP_001264946.2	XP_018114635.1	XP_698834.4	XP_007888440.1					

SFN	NP_006133.1	NP_061224.2	NP_001280105.1								
SH3BP2	NP_001116153.1	NP_036023.3	XP_015141267.1	XP_018096046.1	XP_005155638.1	XP_007903445.1	XP_032800705.1	XP_009861676.2	XP_002608882.1	XP_030848580.1	
SH3PXD2A	NP_055446.2	NP_032044.2	XP_421741.4	XP_018080713.1	XP_009292184.1	XP_007908750.1					
SH3PXD2B	NP_001017995.1	NP_796338.2	XP_425197.3	XP_018107654.1	XP_003200955.2	XP_007908750.1					
SHH	NP_000184.1	NP_033196.1	NP_990152.1	NP_001081782.1	NP_571138.1	XP_007906520.1					
SHOX2	NP_001157150.1	NP_001289286.1	XP_015147272.2	XP_018119245.1	XP_021337040.1	XP_007886592.1	XP_032827815.1				
SIX1	NP_005973.1	NP_033215.2	NP_001038150.1	NP_001089925.1	NP_996978.1	XP_007902022.1	XP_032803935.1				
SKI	NP_003027.1	NP_035515.2	XP_015152425.1	NP_001084112.1	NP_571010.3	XP_007901663.1					
SLC10A7	NP_001025169.1	NP_084012.1	XP_015131664.1	NP_001080729.1	NP_001003420.1	XP_007897350.1	XP_032812255.1	XP_002131347.1	XP_035682414.1	XP_786622.3	XP_002168639.1
SLC25A21	NP_085134.1	NP_766165.2	XP_015143145.1	NP_001090527.1	NP_001070100.1	XP_007891544.1	XP_032804225.1	XP_002130378.1		XP_030854904.1	
SLC26A2	NP_000103.2	NP_031911.1	XP_025010793.1	XP_018111616.1	XP_685114.1	XP_007910486.1	XP_032806612.1				
SLC27A4	NP_005085.2	NP_036119.1	XP_015135039.1	XP_018096544.1	NP_001017737.1	XP_007898514.1					
SLC38A10	NP_001033073.1	NP_001158271.1	XP_004946313.1	XP_018092704.1	XP_017214041.1	XP_007886752.1	XP_032823122.1	XP_002122579.1	XP_035694467.1	XP_030840103.1	XP_012558598.1
SLC39A13	NP_001121697.2	NP_080997.1	NP_001008471.1	XP_018112898.1	NP_001005306.3	NP_001279227.1	XP_032812053.1	XP_002120593.1	XP_002609106.1	XP_030841333.1	XP_012554328.1
SMAD2	NP_005892.1	NP_034884.2	NP_989892.1	NP_001084964.1	NP_571441.3	XP_007904996.1					
SMARCA4	NP_001122321.1	NP_001344693.1	NP_990390.1	NP_001165424.1	NP_853634.1	XP_007905293.1					
SMG1	NP_055907.3	NP_001026984.1	XP_414907.4	XP_018094605.1	NP_001073513.2	XP_007892012.1	XP_032817005.1	XP_018672013.2	XP_002607176.1	XP_030839843.1	XP_012553895.1
SMO	NP_005622.1	NP_795970.3	XP_015143674.1	NP_001128704.1	NP_571102.2	XP_007889054.1	XP_032809666.1	NP_001071819.1	XP_019641686.1	XP_030830155.1	
SOSTDC1	NP_056279.1	NP_079588.1	NP_989704.1	NP_001080917.1	NP_001076314.1	XP_007897897.1					
SOX11	NP_003099.1	NP_033260.4	NP_990518.1	NP_001135834.1	NP_571411.1	XP_007902941.1					
SOX9	NP_000337.1	NP_035578.3	NP_989612.1	NP_001084276.1	NP_571718.1	XP_007886826.1					
SPRY2	NP_001305465.1	NP_036027.1	NP_990131.1	NP_001006932.2	NP_001003842.2	XP_007900755.1					
SPRY4	NP_112226.2	NP_036028.2	NP_001073203.1	XP_018107617.1	NP_571901.2	XP_007884890.1					
SRF	NP_003122.1	NP_065239.1	NP_001239070.1	XP_018120351.1	NP_001103996.1	XP_007896558.1	XP_032820927.1	NP_001071826.1	XP_002610547.1	XP_030854625.1	XP_002156545.1
SUFU	NP_057253.2	NP_056567.2	NP_989595.1	XP_018082933.1	NP_958466.1	XP_007909051.1	XP_032808399.1	XP_002129739.1	XP_019625825.1	XP_796446.2	XP_012560454.1
TBX1	NP_542378.1	NP_035662.1	XP_025011481.1	NP_001083914.1	NP_899182.1	XP_007899995.1					
TBX15	NP_001317606.1	NP_033349.2	XP_416537.3	XP_018101281.1	NP_001315362.1	XP_007894068.1					
TCOF1	NP_001358552.1	NP_035682.1	XP_015149272.1	XP_018109911.1	XP_002664303.1						
TENT5A	NP_060103.2	NP_001153851.1	XP_015140113.2	XP_018120961.1	XP_005157917.1	XP_007903243.1					
TFAP2A	NP_001358995.1	NP_035677.3	NP_990425.1	NP_001089041.1	NP_789829.2	XP_007899085.1	XP_032810894.1	NP_001071986.1	XP_002593633.1	XP_030839379.1	
TGDS	NP_055120.1	NP_083854.3	XP_416988.1	NP_001088301.1	NP_956111.1	XP_007888979.1					
TGFB2	NP_001129071.1	NP_033393.2	NP_001026216.2	NP_001079195.1	NP_919366.1	XP_007902706.1	XP_032818531.1				
TGFBR2	NP_003233.4	NP_033397.3	NP_990759.1	XP_018122960.1	NP_878275.2	XP_007887981.1	XP_032812992.1	NP_001071836.1	XP_002612985.1	XP_030828751.1	
TIPARP	NP_056323.2	NP_849223.2	XP_015147299.1	XP_018119254.1	NP_001038484.1	XP_007886976.1	XP_032824333.1				
TMEM67	NP_714915.3	NP_808529.3	XP_418334.3	XP_018079349.1	XP_700974.4	XP_007900910.1	XP_032813422.1	XP_009862218.1	XP_002593941.1	XP_030831148.1	XP_012554486.1
TNFRSF11B	NP_002537.3	NP_032790.3	NP_001028813.1	XP_018123714.1	NP_001036153.2	XP_007905722.1	XP_032810850.1		XP_002610826.1		
TNFSF11	NP_003692.1	NP_035743.2	NP_001076830.1	XP_018105429.1	XP_002663397.2	XP_007892566.1					
TP53	NP_000537	NP_035770	NP_990595	NP_001081567	NP_001258749	AEW46988					
TP53BP2	NP_001026855.2	NP_775554.2	XP_419394.4	XP_018117469.1	NP_001191186.1	XP_007895021.1					
TP63	NP_003713.3	NP_001120731.1	NP_989682.1	XP_018121105.1	NP_694518.1	AEW46989.1					
TRAPPC10	NP_003265.3	NP_001074524.1	XP_003640553.2	XP_018104503.1	XP_002663888.2	XP_007901780.1	XP_032828638.1	XP_018667831.1	XP_035685725.1	XP_030850473.1	XP_012556503.1
TRPS1	NP_054831.2	NP_114389.2	XP_025003908.1	XP_018124131.1	NP_001171405.1	XP_007895696.1	XP_032817392.1				
TTC28	NP_001138890.1	NP_001254551.1	XP_015130973.1	XP_018099538.1	XP_017213483.1	XP_007908725.1	XP_032803281.1	XP_026690437.1	XP_002596602.1	XP_030847701.1	XP_012559564.1
TWSG1	NP_065699.1	NP_075540.1	XP_025002764.1	NP_001079870.1	NP_571872.2	XP_007895440.1	XP_032813556.1	XP_018672191.1	XP_002593913.1		
VEGFA	NP_001020537.2	NP_001020421.2	NP_990373.1	NP_001079058.1	NP_571483.1	XP_007892525.1					
VPS25	NP_115729.1	NP_081052.2	XP_003642862.1	NP_001087424.1	NP_001082831.2	XP_007895107.1	XP_032831290.1	XP_002126561.1	XP_002598439.1	XP_030830088.1	XP_002156482.1
WDR11	NP_060587.8	NP_758459.2	XP_004942386.1	XP_018080623.1	XP_687231.3	XP_007902084.1	XP_032807825.1	XP_018673256.1	XP_035673019.1	XP_030841145.1	XP_012556157.1
WDR19	NP_079408.3	NP_700440.2	XP_015141146.2	XP_018084648.1	XP_021331990.1	XP_007887304.1	XP_032811146.1	XP_002124215.1	XP_002597830.1	XP_030836953.1	XP_012557446.1
WNT3A	NP_149122.1	NP_033548.1	NP_001165072.1	NP_001079343.1	NP_001007186.1	XP_007905574.1	XP_032802062.1	XP_009859675.1	XP_019618001.1	XP_030830597.1	
WNT5A	NP_003383.3	NP_033550.2	NP_990218.1	NP_001079345.1	NP_001073303.1	XP_007888715.1					
WWP2	NP_001257383.1	NP_080106.1	XP_015148083.1	XP_018113415.1	XP_017209576.1	XP_007887479.1					
XYLT1	NP_071449.1	NP_783576.2	XP_015150019.1	NP_001085934.1	NP_001165868.1	XP_007892011.1	XP_032816793.1				
YBX1	NP_004550.2	NP_035862.2	XP_015152295.1	NP_001079367.1	NP_571695.1	NP_001279438.1					
ZEB1	NP_001121600.1	NP_035676.1	NP_990462.1	NP_001085962.1	NP_571784.1	XP_007903615.1					
ZIC3	NP_003404.1	NP_033601.2	XP_420237.5	NP_001081088.1	NP_001001950.1	XP_007890960.1					
ZIC5	NP_149123.2	NP_075363.1	XP_025009408.1	NP_001079126.1	NP_991290.1	XP_007904496.1					
ZMPSTE24	NP_005848.2	NP_766288.1	XP_417720.1	NP_001087923.1	NP_956186.1	XP_007892978.1	XP_032800885.1	XP_004226161.1	XP_002599069.1	XP_030850470.1	XP_002154940.2
ZNF366	NP_689838.1	NP_001004149.1	XP_429153.4	XP_018120574.1	XP_698186.3	XP_007897984.1	XP_032824509.1		XP_002592273.1		

Supplementary Table 6: Comprehensive overview of the preclinical and clinical research studies on the hub JIGs that are deregulated in highly-invasive cell lines of CCLE. The drivers of tumor progression predicted using the key innovation-driven model are in agreement with existing experimental data

GENE$	PRECLINICAL STUDIES			TRANSLATIONAL RESEARCH		REFs
	Cancer type(s)	Effect on cell invasion and/or metastasis	Study system(s)	Clinical trials&	Approved/marketed drugs	
**BMP4**	breast, colon, esophageal squamous cell carcinoma, glioma, hepatocellular, melanoma	promoting or inhibiting	cancer cell lines, mouse xenografts, patient samples	phase 1-2	ND*	[1-8]
**CDON**	prostate, endometrial, colorectal	promoting	cancer cell lines, mouse xenografts, patient samples	ND	ND	[9-11]
**EGFR**	breast, lung, melanoma, prostate	promoting	cancer cell lines, mouse xenografts, patient samples	phase 1-4	cetuximab, dacomitinib, erlotinib, gefitinib, lapatinib, necitumumab, neratinib, osimertinib, panitumumab, vandetanib	[12-20]
**FBN1**	ovarian	promoting	cancer cell lines, mouse xenografts	ND	ND	[21]
**FGF18**	breast, lung, ovarian, gastric	promoting	cancer cell lines, mouse xenografts, patient samples	observational	ND	[22-26]
**FGF9**	gastric, prostate, ovarian	promoting	cancer cell lines, mouse xenografts, patients samples	ND	ND	[27-32]
**FGFR2**	breast, colorectal, gastric	promoting	cancer cell lines, mouse xenografts, patient samples	phase 1-4	erdafitinib: FDA-approved for metastatic bladder cancer; pemigatinib: FDA approved for locally advanced or metastatic cholangiocarcinoma	[33-39]

**FGFR3**	bladder, hepatocellular	promoting	cancer cell lines, mouse xenografts	phase 1-3	erdafitinib: FDA-approved for metastatic bladder cancer; pemigatinib: FDA approved for locally advanced or metastatic cholangiocarcinoma	[38-41]
**GLI2**	bladder, gastric, glioblastoma, hepatocellular, melanoma, osteosarcoma, prostate	promoting	cancer cell lines, mouse xenografts	ND	ND	[42-49]
**GLI3**	colorectal, lung, oral squamous cell carcinoma	promoting	cancer cell lines, mouse xenografts	ND	ND	[50-52]
**GPC3**	breast, gastric, hepatocellular, ovarian	promoting or inhibiting	cancer cell lines, mouse xenografts, patient samples	phase 1-2	ND	[53-58]
**ITGB1**	colorectal, esophageal, ovarian	promoting	cancer cell lines, mouse xenografts, patient samples	ND	ND	[59-63]
**KDR**	breast, carcinoid, gastric, pancreas, prostate	promoting	cancer cell lines, mouse xenografts, patient samples	phase 1-4	axitinib, bevacizumab, cabozantinib, lenvatinib, pazopanib, ponatinib, ramucirumab, regorafenib, sorafenib, sunitinib, vandetanib, ziv-aflibercept	[64],[65],[66],[67]
**LYN**	bladder, breast, colorectal, gastric, sarcoma	promoting	cancer cell lines, mouse xenografts, patient samples	phase 1-2	ND	[68],[69],[70],[71], [72],[73]
**MEF2C**	breast, hepatocellular	promoting	cancer cell lines, mouse xenografts	ND	ND	[74], [75]
**MMP2**	breast, colorectal, lung	promoting	cancer cell lines, mouse xenografts, knockout mice	phase 1-2	ND	[76-79]
**PDGFRB**	breast, colorectal, gastric, sarcoma	promoting	cancer cell lines, mouse xenografts, patient samples	phase 1-2	ND	[80,81],[82],[83]

**PITX2**	breast, ovarian, prostate	promoting	cancer cell lines, mouse xenografts, patient samples	ND	ND	[84],[85],[86]
**QSOX1**	breast, lung, pancreas, prostate	promoting	cancer cell lines, mouse xenografts	diagnostic	ND	[87],[88],[89],[90]
**RBBP7**	Esophageal, squamous lung	promoting	cancer cell lines	ND	ND	[91],[92]
**SFN**	lung, pancreatic	promoting	cancer cell lines, mouse xenografts, patient samples	ND	ND	[93-98]
**SHH**	bladder, breast, gastric, lung, oral squamous cell carcinoma, osteosarcoma, prostate	promoting	cancer cell lines, mouse xenografts, patient samples	early phase 1, phase 1-3	glasdegib: FDA-approved for AML<br>sonidegib: FDA-approved for locally advanced basal cell carcinoma<br>vismodegib: FDA-approved for metastatic basal cell carcinoma	[99-111]
**SPRY2**	colon, glioblastoma, lung, ovarian, renal cell carcinoma, rhabdomyosarcoma, prostate	promoting or inhibiting	cancer cell lines, mouse xenografts, patient samples	ND	ND	[112-120]
**VEGFA**	breast, colorectal, glioblastoma, lung, renal carcinoma, ovarian, sarcoma	promoting	cancer cell lines, mouse xenografts, patient samples	early phase 1, phase 1-4	axitinib, bevacizumab, cabozantinib, lenvatinib, pazopanib, ponatinib, ramucirumab, regorafenib, sorafenib, sunitinib, vandetanib, ziv-aflibercept	[121-124]
**WNT5A**	breast, cervical, colon, lung, melanoma, ovarian	promoting or inhibiting	cancer cell lines, mouse xenografts, patient samples	phase 1-2	ND	[125],[126],[127],[128],[129],[130],[131]
**DYNC2H1**	gastric	frequently mutated in gastric tumors with N3 stage lymph node metastasis	patient samples	ND	ND	[132]

**EDNRA**	bladder, breast, colon, head and neck, gastric, melanoma, nasopharynx, ovarian, prostate	promoting	cancer cell lines, mouse xenografts	phase 1-3	ND	[133-138]
**FBN2**	lung	frequently methylated in nodal metastases and advanced stages	patient samples	ND	ND	[139]
**GAS1**	breast, colorectal, gastric, hepatocellular, melanoma	inhibiting	cancer cell lines, mouse xenografts, patient samples	ND	ND	[140-146]
**IFT57**	breast, prostate	associated with disease recurrence	patient samples	ND	ND	[147,148]
**LRP2**	melanoma, renal cell carcinoma, prostate	associated with disease recurrence	patient samples	ND	ND	[149-151]
**SMO**	breast, head and neck squamous cell carcinoma	promoting	cancer cell lines, mouse xenografts, patient samples	early phase 1, phase 1-2	vismodegib: FDA-approved for metastatic basal cell carcinoma	[111] [152,153]
**WNT3A**	colon, lung	promoting	cancer cell lines, mouse xenografts, patient samples	ND	ND	[154,155]

$ Grey-colored lines highlight gnathostome-specific genes, and white lines genes of pre-gnathostome origin
& under clinical tests as targets of monotherapies/combination therapies or as biomarkers
*ND - *not determined*

SUPPLEMENTARY REFERENCES

1. Zeng S, Zhang Y, Ma J, Deng G, Qu Y, et al. (2017) BMP4 promotes metastasis of hepatocellular carcinoma by an induction of epithelial–mesenchymal transition via upregulating ID2. Cancer letters 390: 67-76.
2. Guo D, Huang J, Gong J (2012) Bone morphogenetic protein 4 (BMP4) is required for migration and invasion of breast cancer. Molecular and cellular biochemistry 363: 179-190.
3. Ampuja M, Alarmo E, Owens P, Havunen R, Gorska A, et al. (2016) The impact of bone morphogenetic protein 4 (BMP4) on breast cancer metastasis in a mouse xenograft model. Cancer letters 375: 238-244.
4. Rothhammer T, Poser I, Soncin F, Bataille F, Moser M, et al. (2005) Bone morphogenic proteins are overexpressed in malignant melanoma and promote cell invasion and migration. Cancer research 65: 448-456.
5. Deng H, Makizumi R, Ravikumar T, Dong H, Yang W, et al. (2007) Bone morphogenetic protein-4 is overexpressed in colonic adenocarcinomas and promotes migration and invasion of HCT116 cells. Experimental cell research 313: 1033-1044.
6. Zhou Y, Liu Y, Zhang J, Yu D, Li A, et al. (2020) Autocrine BMP4 Signaling Enhances Tumor Aggressiveness via Promoting Wnt/β-Catenin Signaling in IDH1-mutant Gliomas. Translational Oncology 13: 125-134.
7. Sachdeva R, Wu M, Johnson K, Kim H, Celebre A, et al. (2019) BMP signaling mediates glioma stem cell quiescence and confers treatment resistance in glioblastoma. Scientific reports 9: 1-14.
8. Eckhardt BL, Cao Y, Redfern AD, Chi LH, Burrows AD, et al. (2020) Activation of canonical BMP4-SMAD7 signaling suppresses breast cancer metastasis. Cancer Research.
9. Hayashi T, Oue N, Sakamoto N, Anami K, Oo HZ, et al. (2011) Identification of transmembrane protein in prostate cancer by the Escherichia coli ampicillin secretion trap: expression of CDON is involved in tumor cell growth and invasion. Pathobiology 78: 277-284.
10. He X, Lei S, Zhang Q, Ma L, Li N, et al. (2020) Deregulation of cell adhesion molecules is associated with progression and poor outcomes in endometrial cancer: Analysis of The Cancer Genome Atlas data. Oncology letters 19: 1906-1914.
11. Delloye-Bourgeois C, Gibert B, Rama N, Delcros J-G, Gadot N, et al. (2013) Sonic Hedgehog promotes tumor cell survival by inhibiting CDON pro-apoptotic activity. PLoS biology 11.
12. Brand TM, Iida M, Stein AP, Corrigan KL, Braverman CM, et al. (2014) AXL mediates resistance to cetuximab therapy. Cancer research 74: 5152-5164.
13. Ciardiello F, De Vita F, Orditura M, Tortora G (2004) The role of EGFR inhibitors in nonsmall cell lung cancer. Current opinion in oncology 16: 130-135.
14. Hu S, Fu W, Li T, Yuan Q, Wang F, et al. (2017) Antagonism of EGFR and Notch limits resistance to EGFR inhibitors and radiation by decreasing tumor-initiating cell frequency. Science translational medicine 9: eaag0339.
15. Lim S-O, Li C-W, Xia W, Lee H-H, Chang S-S, et al. (2016) EGFR signaling enhances aerobic glycolysis in triple-negative breast cancer cells to promote tumor growth and immune escape. Cancer research 76: 1284-1296.
16. Boone B, Jacobs K, Ferdinande L, Taildeman J, Lambert J, et al. (2011) EGFR in melanoma: clinical significance and potential therapeutic target. Journal of cutaneous pathology 38: 492-502.
17. Ueno Y, Sakurai H, Tsunoda S, Choo MK, Matsuo M, et al. (2008) Heregulin-induced activation of ErbB3 by EGFR tyrosine kinase activity promotes tumor growth and metastasis in melanoma cells. International journal of cancer 123: 340-347.
18. Gan Y, Shi C, Inge L, Hibner M, Balducci J, et al. (2010) Differential roles of ERK and Akt pathways in regulation of EGFR-mediated signaling and motility in prostate cancer cells. Oncogene 29: 4947-4958.
19. Kelly MP, Lee ST, Lee FT, Smyth FE, Davis ID, et al. (2009) Therapeutic efficacy of 177Lu-CHX-A ″-DTPA-hu3S193 radioimmunotherapy in prostate cancer is enhanced by EGFR inhibition or docetaxel chemotherapy. The Prostate 69: 92-104.
20. The drugs.com. https://www.drugs.com/drug-class/egfr-inhibitors.html. Accessed 1 May 2020
21. Block KI, Gyllenhaal C, Lowe L, Amedei A, Amin A, et al. (2015) Designing a broad-spectrum integrative approach for cancer prevention and treatment. Semin Cancer Biol 35 Suppl: S276-S304.

22. Wei W, Mok SC, Oliva E, Kim S-h, Mohapatra G, et al. (2013) FGF18 as a prognostic and therapeutic biomarker in ovarian cancer. The Journal of clinical investigation 123: 4435-4448.
23. Wei W, Mohapatra G, Birrer MJ (2012) The FGF18/FGFR4 amplicon: Novel therapeutic biomarkers for ovarian cancer. AACR.
24. Yu Z, Lou L, Zhao Y (2018) Fibroblast growth factor 18 promotes the growth, migration and invasion of MDA-MB-231 cells. Oncology reports 40: 704-714.
25. Chen T, Gong W, Tian H, Wang H, Chu S, et al. (2017) Fibroblast growth factor 18 promotes proliferation and migration of H460 cells via the ERK and p38 signaling pathways. Oncology reports 37: 1235-1242.
26. Zhang J, Zhou Y, Huang T, Wu F, Pan Y, et al. (2019) FGF18, a prominent player in FGF signaling, promotes gastric tumorigenesis through autocrine manner and is negatively regulated by miR-590-5p. Oncogene 38: 33-46.
27. Teishima J, Yano S, Shoji K, Hayashi T, Goto K, et al. (2014) Accumulation of FGF9 in prostate cancer correlates with epithelial-to-mesenchymal transition and induction of VEGF-A expression. Anticancer research 34: 695-700.
28. Cui G, Shao M, Gu X, Guo H, Zhang S, et al. (2019) The value of FGF9 as a novel biomarker in the diagnosis of prostate cancer. Artificial cells, nanomedicine, and biotechnology 47: 2241-2245.
29. Sun C, Fukui H, Hara K, Zhang X, Kitayama Y, et al. (2015) FGF9 from cancer-associated fibroblasts is a possible mediator of invasion and anti-apoptosis of gastric cancer cells. BMC cancer 15: 333.
30. Wang J, Tan X, Guo Q, Lin X, Huang Y, et al. (2020) FGF9 inhibition by a novel binding peptide has efficacy in gastric and bladder cancer per se and reverses resistance to cisplatin. Pharmacological Research 152: 104575.
31. Bhattacharya R, Ray Chaudhuri S, Roy SS (2018) FGF9-induced ovarian cancer cell invasion involves VEGF-A/VEGFR2 augmentation by virtue of ETS1 upregulation and metabolic reprogramming. Journal of cellular biochemistry 119: 8174-8189.
32. Huang Y, Jin C, Hamana T, Liu J, Wang C, et al. (2015) Overexpression of FGF9 in prostate epithelial cells augments reactive stroma formation and promotes prostate cancer progression. International journal of biological sciences 11: 948.
33. Matsuda Y, Ishiwata T, Yamahatsu K, Kawahara K, Hagio M, et al. (2011) Overexpressed fibroblast growth factor receptor 2 in the invasive front of colorectal cancer: a potential therapeutic target in colorectal cancer. Cancer letters 309: 209-219.
34. Matsuda Y, Hagio M, Seya T, Ishiwata T (2012) Fibroblast growth factor receptor 2 IIIc as a therapeutic target for colorectal cancer cells. Molecular cancer therapeutics 11: 2010-2020.
35. Wang J, Liu X-t, Huang H, Xiao G, Zhou Z-y, et al. (2011) Antitumor activity of a recombinant soluble ectodomain of mutant human fibroblast growth factor receptor-2 IIIc. Molecular cancer therapeutics 10: 1656-1666.
36. Kim HS, Kim JH, Jang HJ, Han B, Zang DY (2019) Pathological and Prognostic Impacts of FGFR2 Overexpression in Gastric Cancer: A Meta-Analysis. Journal of Cancer 10: 20.
37. Wein L, Savas P, Van Geelen C, Caramia F, Moodie K, et al. (2017) FGFR2 amplification in metastatic hormone-positive breast cancer and response to an mTOR inhibitor. Annals of Oncology 28: 2025-2027.
38. The U.S. Food and Drug Administration. https://www.accessdata.fda.gov/drugsatfda_docs/label/2020/213736s000lbl.pdf. Accessed 1 May 2020.
39. Montazeri K, Bellmunt J (2020) Erdafitinib for the treatment of metastatic bladder cancer. Expert Review of Clinical Pharmacology.
40. Qing J, Du X, Chen Y, Chan P, Li H, et al. (2009) Antibody-based targeting of FGFR3 in bladder carcinoma and t (4; 14)-positive multiple myeloma in mice. The Journal of clinical investigation 119: 1216-1229.
41. Liu X, Jing X, Cheng X, Ma D, Jin Z, et al. (2016) FGFR3 promotes angiogenesis-dependent metastasis of hepatocellular carcinoma via facilitating MCP-1-mediated vascular formation. Medical Oncology 33: 46.
42. Thiyagarajan S, Bhatia N, Reagan-Shaw S, Cozma D, Thomas-Tikhonenko A, et al. (2007) Role of GLI2 transcription factor in growth and tumorigenicity of prostate cells. Cancer research 67: 10642-10646.

43. Huang D, Wang Y, Xu L, Chen L, Cheng M, et al. (2018) GLI2 promotes cell proliferation and migration through transcriptional activation of ARHGEF16 in human glioma cells. Journal of Experimental & Clinical Cancer Research 37: 1-17.
44. Alexaki V-I, Javelaud D, Van Kempen LC, Mohammad KS, Dennler S, et al. (2010) GLI2-mediated melanoma invasion and metastasis. Journal of the National Cancer Institute. pp. 1148-1159.
45. Faiao-Flores F, Alves-Fernandes D, Pennacchi PC, Sandri S, Vicente ALSA, et al. (2017) Targeting the hedgehog transcription factors GLI1 and GLI2 restores sensitivity to vemurafenib-resistant human melanoma cells. Oncogene 36: 1849-1861.
46. Zhang D, Cao L, Li Y, Lu H, Yang X, et al. (2013) Expression of glioma-associated oncogene 2 (Gli 2) is correlated with poor prognosis in patients with hepatocellular carcinoma undergoing hepatectomy. World journal of surgical oncology 11: 25.
47. Ruan Q, Fang Z-y, Cui S-z, Zhang X-l, Wu Y-b, et al. (2015) Thermo-chemotherapy Induced miR-218 upregulation inhibits the invasion of gastric cancer via targeting Gli2 and E-cadherin. Tumor Biology 36: 5807-5814.
48. Raven PA, Lysakowski S, Tan Z, D'Costa NM, Moskalev I, et al. (2019) Inhibition of GLI2 with antisense-oligonucleotides: A potential therapy for the treatment of bladder cancer. Journal of cellular physiology 234: 20634-20647.
49. Nagao-Kitamoto H, Nagata M, Nagano S, Kitamoto S, Ishidou Y, et al. (2015) GLI2 is a novel therapeutic target for metastasis of osteosarcoma. International journal of cancer 136: 1276-1284.
50. Rodrigues MFSD, Miguita L, De Andrade NP, Heguedusch D, Rodini CO, et al. (2018) GLI3 knockdown decreases stemness, cell proliferation and invasion in oral squamous cell carcinoma. International journal of oncology 53: 2458-2472.
51. Fu L, Wu H, Cheng SY, Gao D, Zhang L, et al. (2016) Set7 mediated Gli3 methylation plays a positive role in the activation of Sonic Hedgehog pathway in mammals. Elife 5: e15690.
52. Trnski D, Sabol M, Gojević A, Martinić M, Ozretić P, et al. (2015) GSK3β and Gli3 play a role in activation of Hedgehog-Gli pathway in human colon cancer—Targeting GSK3β downregulates the signaling pathway and reduces cell proliferation. Biochimica et Biophysica Acta (BBA)-Molecular Basis of Disease 1852: 2574-2584.
53. Capurro MI, Xiang Y-Y, Lobe C, Filmus J (2005) Glypican-3 promotes the growth of hepatocellular carcinoma by stimulating canonical Wnt signaling. Cancer research 65: 6245-6254.
54. Peters M, Farias E, Colombo L, Filmus J, Puricelli L, et al. (2003) Inhibition of invasion and metastasis by glypican-3 in a syngeneic breast cancer model. Breast cancer research and treatment 80: 221-232.
55. Castillo LF, Tascón R, Huvelle MAL, Novack G, Llorens MC, et al. (2016) Glypican-3 induces a mesenchymal to epithelial transition in human breast cancer cells. Oncotarget 7: 60133.
56. Liu Y, Zheng D, Liu M, Bai J, Zhou X, et al. (2015) Downregulation of glypican-3 expression increases migration, invasion, and tumorigenicity of human ovarian cancer cells. Tumor Biology 36: 7997-8006.
57. Han S, Ma X, Zhao Y, Zhao H, Batista A, et al. (2016) Identification of Glypican-3 as a potential metastasis suppressor gene in gastric cancer. Oncotarget 7: 44406.
58. Guo M, Zhang H, Zheng J, Liu Y (2020) Glypican-3: A New Target for Diagnosis and Treatment of Hepatocellular Carcinoma. Journal of Cancer 11: 2008.
59. Zhang L, Zou W (2015) Inhibition of integrin β1 decreases the malignancy of ovarian cancer cells and potentiates anticancer therapy via the FAK/STAT1 signaling pathway. Molecular medicine reports 12: 7869-7876.
60. Mori R, Ishiguro H, Kuwabara Y, Kimura M, Mitsui A, et al. (2008) Targeting β1 integrin restores sensitivity to docetaxel of esophageal squamous cell carcinoma. Oncology reports 20: 1345-1351.
61. Xu Z, Zou L, Ma G, Wu X, Huang F, et al. (2017) Integrin β1 is a critical effector in promoting metastasis and chemo-resistance of esophageal squamous cell carcinoma. American journal of cancer research 7: 531.
62. Song J, Zhang J, Wang J, Wang J, Guo X, et al. (2015) β1 integrin mediates colorectal cancer cell proliferation and migration through regulation of the Hedgehog pathway. Tumor Biology 36: 2013-2021.
63. Barnawi R, Al-Khaldi S, Colak D, Tulbah A, Al-Tweigeri T, et al. (2019) β1 Integrin is essential for fascin-mediated breast cancer stem cell function and disease progression. International journal of cancer 145: 830-841.

64. Doi Y, Yashiro M, Yamada N, Amano R, Noda S, et al. (2012) VEGF-A/VEGFR-2 signaling plays an important role for the motility of pancreas cancer cells. Annals of surgical oncology 19: 2733-2743.
65. Yan J-D, Liu Y, Zhang Z-Y, Liu G-Y, Xu J-H, et al. (2015) Expression and prognostic significance of VEGFR-2 in breast cancer. Pathology-Research and Practice 211: 539-543.
66. Silva SR, Bowen KA, Rychahou PG, Jackson LN, Weiss HL, et al. (2011) VEGFR-2 expression in carcinoid cancer cells and its role in tumor growth and metastasis. International journal of cancer 128: 1045-1056.
67. Lian L, Li X-L, Xu M-D, Li X-M, Wu M-Y, et al. (2019) VEGFR2 promotes tumorigenesis and metastasis in a pro-angiogenic-independent way in gastric cancer. BMC cancer 19: 1-15.
68. Guan H, Zhou Z, Gallick GE, Jia S-F, Morales J, et al. (2008) Targeting Lyn inhibits tumor growth and metastasis in Ewing's sarcoma. Molecular cancer therapeutics 7: 1807-1816.
69. Tabariès S, Annis MG, Hsu BE, Tam CE, Savage P, et al. (2015) Lyn modulates Claudin-2 expression and is a therapeutic target for breast cancer liver metastasis. Oncotarget 6: 9476.
70. Choi Y-L, Bocanegra M, Kwon MJ, Shin YK, Nam SJ, et al. (2010) LYN is a mediator of epithelial-mesenchymal transition and a target of dasatinib in breast cancer. Cancer research 70: 2296-2306.
71. Su R, Zhang J (2020) Oncogenic role of LYN in human gastric cancer via the Wnt/β-catenin and AKT/mTOR pathways. Experimental and Therapeutic Medicine 20: 646-654.
72. Liu S, Hao X, Ouyang X, Dong X, Yang Y, et al. (2016) Tyrosine kinase LYN is an oncotarget in human cervical cancer: a quantitative proteomic based study. Oncotarget 7: 75468.
73. Hao JM, Chen JZ, Sui HM, Si-Ma XQ, Li GQ, et al. (2010) A five-gene signature as a potential predictor of metastasis and survival in colorectal cancer. The Journal of Pathology: A Journal of the Pathological Society of Great Britain and Ireland 220: 475-489.
74. Yu W, Huang C, Wang Q, Huang T, Ding Y, et al. (2014) MEF2 transcription factors promotes EMT and invasiveness of hepatocellular carcinoma through TGF-β1 autoregulation circuitry. Tumor Biology 35: 10943-10951.
75. Sereno M, Haskó J, Molnár K, Medina SJ, Reisz Z, et al. (2020) Downregulation of circulating miR 802-5p and miR 194-5p and upregulation of brain MEF2C along breast cancer brain metastasization. Molecular oncology 14: 520-538.
76. Das K, Prasad R, Ansari SA, Roy A, Mukherjee A, et al. (2018) Matrix metalloproteinase-2: A key regulator in coagulation proteases mediated human breast cancer progression through autocrine signaling. Biomedicine & Pharmacotherapy 105: 395-406.
77. Mendes O, Kim H-T, Lungu G, Stoica G (2007) MMP2 role in breast cancer brain metastasis development and its regulation by TIMP2 and ERK1/2. Clinical & experimental metastasis 24: 341-351.
78. Dong W, Li H, Zhang Y, Yang H, Guo M, et al. (2011) Matrix metalloproteinase 2 promotes cell growth and invasion in colorectal cancer. Acta Biochim Biophys Sin 43: 840-848.
79. Bates AL, Pickup MW, Hallett MA, Dozier EA, Thomas S, et al. (2015) Stromal matrix metalloproteinase 2 regulates collagen expression and promotes the outgrowth of experimental metastases. The Journal of pathology 235: 773-783.
80. Wang YX, Mandal D, Wang S, Hughes D, Pollock RE, et al. (2009) Inhibiting platelet-derived growth factor β reduces Ewing's sarcoma growth and metastasis in a novel orthotopic human xenograft model. In vivo 23: 903-909.
81. Thies KA, Hammer AM, Hildreth BE, Steck SA, Spehar JM, et al. (2020) Stromal platelet-derived growth factor receptor-β signaling promotes breast cancer metastasis in the brain. Cancer research.
82. Guo Y, Yin J, Zha L, Wang Z (2013) Clinicopathological significance of platelet-derived growth factor B, platelet-derived growth factor receptor-β, and E-cadherin expression in gastric carcinoma. Contemporary oncology 17: 150.
83. Fujino S, Miyoshi N, Ohue M, Takahashi Y, Yasui M, et al. (2018) Platelet-derived growth factor receptor-β gene expression relates to recurrence in colorectal cancer. Oncology reports 39: 2178-2184.
84. Fung FK, Chan DW, Liu VW, Leung TH, Cheung AN, et al. (2012) Increased expression of PITX2 transcription factor contributes to ovarian cancer progression. PloS one 7: e37076.
85. Pillai SG, Dasgupta N, Siddappa CM, Watson MA, Fleming T, et al. (2015) Paired-like Homeodomain Transcription factor 2 expression by breast cancer bone marrow disseminated tumor cells is associated with early recurrent disease development. Breast cancer research and treatment 153: 507-517.

86. Vela I, Morrissey C, Zhang X, Chen S, Corey E, et al. (2014) PITX2 and non-canonical Wnt pathway interaction in metastatic prostate cancer. Clinical & experimental metastasis 31: 199-211.
87. Katchman BA, Ocal IT, Cunliffe HE, Chang Y-H, Hostetter G, et al. (2013) Expression of quiescin sulfhydryl oxidase 1 is associated with a highly invasive phenotype and correlates with a poor prognosis in Luminal B breast cancer. Breast Cancer Research 15: R28.
88. Sung H-J, Ahn J-M, Yoon Y-H, Na S-S, Choi Y-J, et al. (2018) Quiescin sulfhydryl oxidase 1 (QSOX1) secreted by lung cancer cells promotes cancer metastasis. International journal of molecular sciences 19: 3213.
89. Katchman BA, Antwi K, Hostetter G, Demeure MJ, Watanabe A, et al. (2011) Quiescin sulfhydryl oxidase 1 promotes invasion of pancreatic tumor cells mediated by matrix metalloproteinases. Molecular Cancer Research 9: 1621-1631.
90. Baek JA, Song PH, Ko Y, Gu MJ (2018) High expression of QSOX1 is associated with tumor invasiveness and high grades groups in prostate cancer. Pathology-Research and Practice 214: 964-967.
91. Yu N, Zhang P, Wang L, He X, Yang S, et al. (2018) RBBP7 is a prognostic biomarker in patients with esophageal squamous cell carcinoma. Oncology letters 16: 7204-7211.
92. Wang C-L, Wang C-I, Liao P-C, Chen C-D, Liang Y, et al. (2009) Discovery of retinoblastoma-associated binding protein 46 as a novel prognostic marker for distant metastasis in nonsmall cell lung cancer by combined analysis of cancer cell secretome and pleural effusion proteome. Journal of proteome research 8: 4428-4440.
93. Kim Y, Shiba-Ishii A, Nakagawa T, Iemura S-i, Natsume T, et al. (2018) Stratifin regulates stabilization of receptor tyrosine kinases via interaction with ubiquitin-specific protease 8 in lung adenocarcinoma. Oncogene 37: 5387-5402.
94. Shiba-Ishii A, Kim Y, Shiozawa T, Iyama S, Satomi K, et al. (2015) Stratifin accelerates progression of lung adenocarcinoma at an early stage. Molecular cancer 14: 142.
95. Cetintas VB, Tetik A, Cok G, Kucukaslan AS, Kosova B, et al. (2013) Role of 14-3-3σ in resistance to cisplatin in non-small cell lung cancer cells. Cell biology international 37: 78-86.
96. Guweidhi A, Kleeff J, Giese N, Fitori JE, Ketterer K, et al. (2004) Enhanced expression of 14-3-3sigma in pancreatic cancer and its role in cell cycle regulation and apoptosis. Carcinogenesis 25: 1575-1585.
97. Li Z, Dong Z, Myer D, Yip-Schneider M, Liu J, et al. (2010) Role of 14-3-3σ in poor prognosis and in radiation and drug resistance of human pancreatic cancers. BMC cancer 10: 598.
98. Shiba-Ishii A, Kano J, Morishita Y, Sato Y, Minami Y, et al. (2011) High expression of stratifin is a universal abnormality during the course of malignant progression of early-stage lung adenocarcinoma. International journal of cancer 129: 2445-2453.
99. Nishimaki H, Kasai K, Kozaki K-i, Takeo T, Ikeda H, et al. (2004) A role of activated Sonic hedgehog signaling for the cellular proliferation of oral squamous cell carcinoma cell line. Biochemical and biophysical research communications 314: 313-320.
100. Yan R, Peng X, Yuan X, Huang D, Chen J, et al. (2013) Suppression of growth and migration by blocking the Hedgehog signaling pathway in gastric cancer cells. Cellular oncology 36: 421-435.
101. Islam S, Mokhtari R, Noman A, Uddin M, Rahman M, et al. (2016) Sonic hedgehog (Shh) signaling promotes tumorigenicity and stemness via activation of epithelial-to-mesenchymal transition (EMT) in bladder cancer. Molecular carcinogenesis 55: 537-551.
102. Jiang WG, Ye L, Ruge F, Sun P-H, Sanders AJ, et al. (2015) Expression of Sonic Hedgehog (SHH) in human lung cancer and the impact of YangZheng XiaoJi on SHH-mediated biological function of lung cancer cells and tumor growth. Anticancer research 35: 1321-1331.
103. Szczepny A, Rogers S, Jayasekara WSN, Park K, McCloy RA, et al. (2017) The role of canonical and non-canonical Hedgehog signaling in tumor progression in a mouse model of small cell lung cancer. Oncogene 36: 5544-5550.
104. Statkiewicz M, Maryan N, Lipiec A, Grecka E, Grygorowicz MA, et al. (2014) The role of the SHH gene in prostate cancer cell resistance to paclitaxel. The Prostate 74: 1142-1152.
105. Li X, Wang Z, Ma Q, Xu Q, Liu H, et al. (2014) Sonic hedgehog paracrine signaling activates stromal cells to promote perineural invasion in pancreatic cancer. Clinical Cancer Research 20: 4326-4338.

106. Yao Z, Han L, Chen Y, He F, Sun B, et al. (2018) Hedgehog signalling in the tumourigenesis and metastasis of osteosarcoma, and its potential value in the clinical therapy of osteosarcoma. Cell death & disease 9: 1-12.
107. Riaz SK, Ke Y, Wang F, Kayani MA, Malik MFA (2019) Influence of SHH/GLI1 axis on EMT mediated migration and invasion of breast cancer cells. Scientific reports 9: 1-13.
108. Song L, Liu D, Zhao Y, He J, Kang H, et al. (2018) Sinomenine reduces growth and metastasis of breast cancer cells and improves the survival of tumor-bearing mice through suppressing the SHh pathway. Biomedicine & Pharmacotherapy 98: 687-693.
109. The U.S. Food and Drug Administration. https://www.accessdata.fda.gov/drugsatfda_docs/nda/2018/210656Orig1s000TOC.cfm. Accessed 1 May 2020.
110. The U.S. Food and Drug Administration. https://www.accessdata.fda.gov/drugsatfda_docs/label/2017/205266s004lbl.pdf. Accessed 1 May 2020.
111. The U.S. Food and Drug Administration. https://www.accessdata.fda.gov/drugsatfda_docs/label/2012/203388lbl.pdf. Accessed 1 May 2020.
112. Walsh AM, Kapoor GS, Buonato JM, Mathew LK, Bi Y, et al. (2015) Sprouty2 drives drug resistance and proliferation in glioblastoma. Molecular Cancer Research 13: 1227-1237.
113. Park J-W, Wollmann G, Urbiola C, Fogli B, Florio T, et al. (2018) Sprouty2 enhances the tumorigenic potential of glioblastoma cells. Neuro-oncology 20: 1044-1054.
114. Li P, Tao L, Yang J, Cai H, Ju X, et al. (2013) Sprouty2 is associated with prognosis and suppresses cell proliferation and invasion in renal cell carcinoma. Urology 82: 253. e251-253. e257.
115. Sutterlüty H, Mayer C-E, Setinek U, Attems J, Ovtcharov S, et al. (2007) Down-Regulation of Sprouty2 in Non–Small Cell Lung Cancer Contributes to Tumor Malignancy via Extracellular Signal-Regulated Kinase Pathway-Dependent and-Independent Mechanisms. Molecular Cancer Research 5: 509-520.
116. So W-K, Cheng J-C, Fan Q, Wong AS, Huntsman DG, et al. (2015) Loss of Sprouty2 in human high-grade serous ovarian carcinomas promotes EGF-induced E-cadherin down-regulation and cell invasion. FEBS letters 589: 302-309.
117. Cheng J-C, Chang H-M, Xiong S, So W-K, Leung PC (2016) Sprouty2 inhibits amphiregulin-induced down-regulation of E-cadherin and cell invasion in human ovarian cancer cells. Oncotarget 7: 81645.
118. Saini M, Verma A, Mathew SJ (2018) SPRY2 is a novel MET interactor that regulates metastatic potential and differentiation in rhabdomyosarcoma. Cell death & disease 9: 1-15.
119. Holgren C, Dougherty U, Edwin F, Cerasi D, Taylor I, et al. (2010) Sprouty-2 controls c-Met expression and metastatic potential of colon cancer cells: sprouty/c-Met upregulation in human colonic adenocarcinomas. Oncogene 29: 5241-5253.
120. Patel R, Gao M, Ahmad I, Fleming J, Singh LB, et al. (2013) Sprouty2, PTEN, and PP2A interact to regulate prostate cancer progression. The Journal of clinical investigation 123: 1157-1175.
121. Kim M, Jang K, Miller P, Picon-Ruiz M, Yeasky T, et al. (2017) VEGFA links self-renewal and metastasis by inducing Sox2 to repress miR-452, driving Slug. Oncogene 36: 5199-5211.
122. English WR, Lunt SJ, Fisher M, Lefley DV, Dhingra M, et al. (2017) Differential expression of VEGFA isoforms regulates metastasis and response to anti-VEGFA therapy in sarcoma. Cancer research 77: 2633-2646.
123. Kong D-H, Kim MR, Jang JH, Na H-J, Lee S (2017) A review of anti-angiogenic targets for monoclonal antibody cancer therapy. International journal of molecular sciences 18: 1786.
124. The drugs.com. https://www.drugs.com/drug-class/vegf-vegfr-inhibitors.htm. Accessed 1 May 2020.
125. Weeraratna AT, Jiang Y, Hostetter G, Rosenblatt K, Duray P, et al. (2002) Wnt5a signaling directly affects cell motility and invasion of metastatic melanoma. Cancer cell 1: 279-288.
126. Asem M, Young AM, Oyama C, De La Zerda AC, Liu Y, et al. (2020) Host Wnt5a Potentiates Microenvironmental Regulation of Ovarian Cancer Metastasis. Cancer research 80: 1156-1170.
127. Bo H, Zhang S, Gao L, Chen Y, Zhang J, et al. (2013) Upregulation of Wnt5a promotes epithelial-to-mesenchymal transition and metastasis of pancreatic cancer cells. BMC cancer 13: 496.
128. Jiang W, Crossman DK, Mitchell EH, Sohn P, Crowley MR, et al. (2013) WNT5A inhibits metastasis and alters splicing of Cd44 in breast cancer cells. PloS one 8: e58329.

129. Lin L, Liu Y, Zhao W, Sun B, Chen Q (2014) Wnt5A expression is associated with the tumor metastasis and clinical survival in cervical cancer. International journal of clinical and experimental pathology 7: 6072.
130. Wang B, Tang Z, Gong H, Zhu L, Liu X (2017) Wnt5a promotes epithelial-to-mesenchymal transition and metastasis in non-small-cell lung cancer. Bioscience reports 37.
131. Bakker ER, Das AM, Helvensteijn W, Franken PF, Swagemakers S, et al. (2013) Wnt5a promotes human colon cancer cell migration and invasion but does not augment intestinal tumorigenesis in Apc 1638N mice. Carcinogenesis 34: 2629-2638.
132. Choi J-H, Kim Y-B, Ahn JM, Kim MJ, Bae WJ, et al. (2018) Identification of genomic aberrations associated with lymph node metastasis in diffuse-type gastric cancer. Experimental & molecular medicine 50: 1-11.
133. Nie S, Zhou J, Bai F, Jiang B, Chen J, et al. (2014) Role of endothelin A receptor in colon cancer metastasis: in vitro and in vivo evidence. Molecular carcinogenesis 53: E85-E91.
134. Fukui R, Nishimori H, Hata F, Yasoshima T, Ohno K, et al. (2007) Inhibitory effect of endothelin A receptor blockade on tumor growth and liver metastasis of a human gastric cancer cell line. Gastric Cancer 10: 123-128.
135. Rosano L, Spinella F, Salani D, Di Castro V, Venuti A, et al. (2003) Therapeutic targeting of the endothelin a receptor in human ovarian carcinoma. Cancer Research 63: 2447-2453.
136. Mai HQ, Zeng ZY, Feng KT, Ye YL, Zhang CQ, et al. (2006) Therapeutic targeting of the endothelin a receptor in human nasopharyngeal carcinoma. Cancer science 97: 1388-1395.
137. Laurberg JR, Jensen JB, Schepeler T, Borre M, Ørntoft TF, et al. (2014) High expression of GEM and EDNRA is associated with metastasis and poor outcome in patients with advanced bladder cancer. BMC cancer 14: 638.
138. Said N, Theodorescu D (2012) Permissive role of endothelin receptors in tumor metastasis. Life sciences 91: 522-527.
139. Chen H, Suzuki M, Nakamura Y, Ohira M, Ando S, et al. (2005) Aberrant methylation of FBN2 in human non-small cell lung cancer. Lung Cancer 50: 43-49.
140. Wang H, Zhou X, Zhang Y, Zhu H, Zhao L, et al. (2012) Growth arrest-specific gene 1 is downregulated and inhibits tumor growth in gastric cancer. The FEBS journal 279: 3652-3664.
141. Zhao L, Pan Y, Gang Y, Wang H, Jin H, et al. (2009) Identification of GAS1 as an epirubicin resistance-related gene in human gastric cancer cells with a partially randomized small interfering RNA library. Journal of Biological Chemistry 284: 26273-26285.
142. Li Q, Qin Y, Wei P, Lian P, Li Y, et al. (2016) Gas1 inhibits metastatic and metabolic phenotypes in colorectal carcinoma. Molecular Cancer Research 14: 830-840.
143. Jiang Z, Xu Y, Cai S (2011) Down-regulated GAS1 expression correlates with recurrence in stage II and III colorectal cancer. Human pathology 42: 361-368.
144. Gobeil S, Zhu X, Doillon CJ, Green MR (2008) A genome-wide shRNA screen identifies GAS1 as a novel melanoma metastasis suppressor gene. Genes & development 22: 2932-2940.
145. Sacilotto N, Castillo J, Riffo-Campos ÁL, Flores JM, Hibbitt O, et al. (2015) Growth arrest specific 1 (Gas1) gene overexpression in liver reduces the in vivo progression of murine hepatocellular carcinoma and partially restores gene expression levels. PloS one 10.
146. Jiménez A, López-Ornelas A, Estudillo E, González-Mariscal L, González RO, et al. (2014) A soluble form of GAS1 inhibits tumor growth and angiogenesis in a triple negative breast cancer model. Experimental cell research 327: 307-317.
147. Long Q, Xu J, Osunkoya AO, Sannigrahi S, Johnson BA, et al. (2014) Global transcriptome analysis of formalin-fixed prostate cancer specimens identifies biomarkers of disease recurrence. Cancer research 74: 3228-3237.
148. Soady KJ, Kendrick H, Gao Q, Tutt A, Zvelebil M, et al. (2015) Mouse mammary stem cells express prognostic markers for triple-negative breast cancer. Breast Cancer Research 17: 31.
149. Andersen RK, Hammer K, Hager H, Christensen JN, Ludvigsen M, et al. (2015) Melanoma tumors frequently acquire LRP 2/megalin expression, which modulates melanoma cell proliferation and survival rates. Pigment cell & melanoma research 28: 267-280.
150. Gonias SL, Karimi-Mostowfi N, Murray SS, Mantuano E, Gilder AS (2017) Expression of LDL receptor-related proteins (LRPs) in common solid malignancies correlates with patient survival. PloS one 12.

151. Holt SK, Karyadi DM, Kwon EM, Stanford JL, Nelson PS, et al. (2008) Association of megalin genetic polymorphisms with prostate cancer risk and prognosis. Clinical cancer research 14: 3823-3831.
152. Wang L, Duan W, Kang L, Mao J, Yu X, et al. (2014) Smoothened activates breast cancer stem-like cell and promotes tumorigenesis and metastasis of breast cancer. Biomedicine & Pharmacotherapy 68: 1099-1104.
153. Richtig G, Aigelsreiter AM, Asslaber M, Weiland T, Pichler M, et al. (2019) Hedgehog pathway proteins SMO and GLI expression as prognostic markers in head and neck squamous cell carcinoma. Histopathology 75: 118-127.
154. Li C, Song G, Zhang S, Wang E, Cui Z (2015) Wnt3a increases the metastatic potential of non-small cell lung cancer cells in vitro in part via its upregulation of Notch3. Oncology reports 33: 1207-1214.
155. Qi L, Sun B, Liu Z, Cheng R, Li Y, et al. (2014) Wnt3a expression is associated with epithelial-mesenchymal transition and promotes colon cancer progression. Journal of Experimental & Clinical Cancer Research 33: 107.
